# Design, Synthesis, and Application of Fluorescent Ligands Targeting the Intracellular Allosteric Binding Site of the CXC Chemokine Receptor 2

**DOI:** 10.1021/acs.jmedchem.3c00849

**Published:** 2023-07-31

**Authors:** Bianca Maria Casella, James P. Farmer, Desislava N. Nesheva, Huw E. L. Williams, Steven J. Charlton, Nicholas D. Holliday, Charles A. Laughton, Shailesh N. Mistry

**Affiliations:** † Division of Biomolecular Sciences and Medicinal Chemistry, School of Pharmacy, University of Nottingham Biodiscovery Institute, Nottingham NG7 2RD, UK; ‡ Division of Physiology, Pharmacology & Neuroscience, Medical School, School of Life Sciences, University of Nottingham, Nottingham NG7 2UH, UK; § School of Chemistry, University of Nottingham Biodiscovery Institute, Nottingham NG7 2RD, UK; ∥ Excellerate Bioscience Ltd., Biocity, University of Nottingham, Nottingham NG1 1GF, UK; ⊥ OMass Therapeutics Ltd., Oxford OX4 2GX, UK

## Abstract

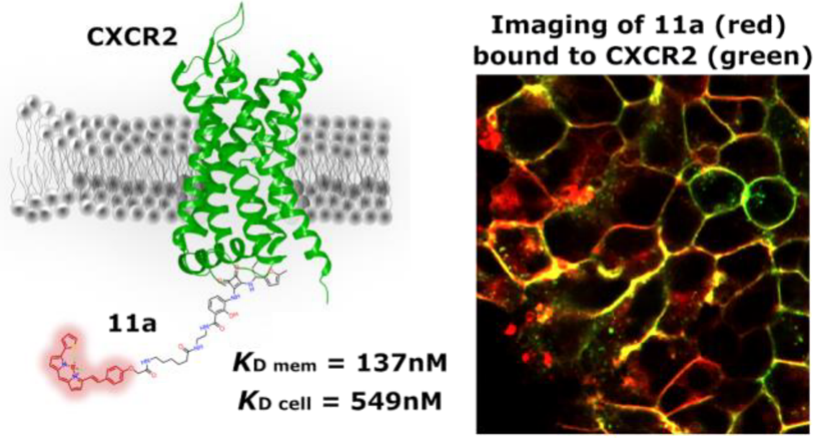

The inhibition of CXC chemokine receptor 2 (CXCR2), a key inflammatory mediator, is a potential strategy in the treatment of several pulmonary diseases and cancers. The complexity of endogenous chemokine interaction with the orthosteric binding site has led to the development of CXCR2 negative allosteric modulators (NAMs) targeting an intracellular pocket near the G protein binding site. Our understanding of NAM binding and mode of action has been limited by the availability of suitable tracer ligands for competition studies, allowing direct ligand binding measurements. Here, we report the rational design, synthesis, and pharmacological evaluation of a series of fluorescent NAMs, based on navarixin (**2**), which display high affinity and preferential binding for CXCR2 over CXCR1. We demonstrate their application in fluorescence imaging and NanoBRET binding assays, in whole cells or membranes, capable of kinetic and equilibrium analysis of NAM binding, providing a platform to screen for alternative chemophores targeting these receptors.

## Introduction

Chemokines are small proteins of around 8–12 kDa^1−3^ involved in inflammatory response regulation, lymphoid organ development, tumor cell growth, and metastasis.^4−6^ They are grouped into three families (CC, CXC, and CX_3_C) according to the position of the first two highly conserved cysteine residues,^1−3,5,7^ with the C-X-C family characterized by the presence of one amino acid between the cysteine residues.^6,8^ Chemokine receptors belong to the Class A G protein-coupled receptor (GPCR) family and are similarly subdivided into CC, CXC, or CX_3_C categories based on the ligand type.^6^ Most are coupled to the G_i_ class of G-proteins,^6^ resulting in a decrease in intracellular cAMP levels following receptor activation.^6,9,10^ Chemokine binding may also induce the recruitment of β-arrestins to mediate receptor desensitization and internalization, alongside other signaling pathways.^11,12^

The CXCR2 receptor subtype, responsive to chemokines such as CXCL8/interleukin8 (IL8) and CXCL1/Gro-alpha, is expressed on a wide range of immune cells, particularly neutrophils, and other cell types including endothelial and epithelial cells.^6,13,14^ In addition, it is present in lung, colon and ovarian cancer cells.^15,16^ CXCR2 is primarily involved in driving chemotaxis and associated processes such as cell motility, integrin expression, and activation but may also be involved in other processes such as phagocytosis and apoptosis.^6,14^ Moreover, CXCR2 regulates neutrophil homeostasis and extravasation,^6,17^ facilitating worsening of acute and chronic inflammation^3,6,18,19^ as well as being involved in angiogenesis and driving tumor metastasis.^20−22^ Given its involvement in a series of diseases,^19^ CXCR2 is a tractable pharmaceutical target, especially in regard to inhibition of inflammatory cell recruitment.^6^ On this basis, CXCR2 antagonists have been developed and clinically evaluated for the treatment of chronic obstructive pulmonary disease (COPD),^6,23−26^ asthma,^3,6,21,24,27^ melanoma,^28,29^ breast cancer,^30^ and colorectal cancer.^31^

**Figure 1:**
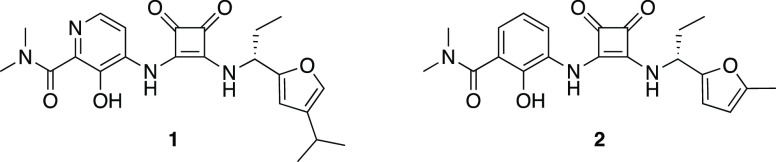
Structures of CXCR2 NAMs 00767013 (**1**) and Sch527123 (*R*-navarixin, **2**).

CXCL8-CXCR2 interaction plays a key role in neutrophil chemotaxis and angiogenesis.^32−34^ The binding of CXCL8 to CXCR2 involves a complex two-step process that has been elucidated through the publication of two cryo-electron microscopy structures (PDB ID: 6LFO and 6LFM).^15^ Competitive inhibition of this complex through small molecule approaches has proven notoriously difficult, which has led to the discovery and development of CXCR2 intracellular negative allosteric modulators (NAMs) as an antagonist strategy.^35−38^ These bind in an intracellular binding pocket located in the cytoplasmic domain, formed by the ends of transmembrane (TM) 1, TM2, TM3, TM6, the loop between TM7 and helix 8, and the *C*-terminus.^15,39,40^ Similar intracellular allosteric binding sites have also been identified in CCR2, CCR7, CCR9, β_2_-adrenoceptor,^15,41−44^ and EP_2_.^45^ The 3,4-diamino-cyclobutenedione class of CXCR2 intracellular NAMs was originally developed by Schering–Plough in the early 2000s.^13,46^ Their proposed binding pose and mechanism of action have been exemplified with the publication of the X-ray crystal structure of CXCR2 in complex with the antagonist 00767013 (**1**, Figure 1) (PDB ID: 6LFL).^15^ The binding of **1** is proposed to sterically interfere with G protein binding due to their overlapping binding sites and stabilizes the inactive state of the receptor.^15^

Sch527123 (*R*-navarixin, **2**, Figure 1)^13,46^ exhibits high potency and affinity at CXCR2 (*K*_D_ = 0.049 ± 0.004 nM) in radioligand binding studies over alternative intracellular modulators such as SB265610.^47^ This high affinity may be the result of a slow dissociation rate from the receptor.^14,40^ However, to directly determine NAM affinity and binding kinetics, a molecular tool that binds competitively with unlabeled ligands to the intracellular allosteric binding site is needed, which can then be used in real-time analysis of ligand binding. High-affinity fluorescent probes are suitable for this purpose and can be employed in several GPCR binding techniques, including bioluminescence resonance energy transfer (BRET) assays.^48−53^ These assays present considerable advantages over radioligand binding assays, traditionally used to characterize ligand binding. A better safety profile allows an easier performance of the assay and disposal of waste materials.^54,55^ Furthermore, fluorescent ligand-based resonance energy transfer assays have the key capability to monitor specific binding in a homogeneous format, without the need to separate bound from the free tracer, and the binding from a single sample can be monitored continuously in real time, enabling kinetic analysis to be performed in a straightforward manner. Recently, new fluorescent probes targeting the intracellular allosteric binding site of chemokine receptors, such as CCR9^56^ and CCR2,^57^ have been reported.^58,59^ Herein, we present the design, synthesis, and pharmacological characterization of a focused library of fluorescently labeled CXCR2 intracellular allosteric ligands, demonstrate their high affinity and a degree of selectivity for the CXCR2 receptor by both imaging and kinetic NanoBRET measurements, and show their application to the direct measurement of unlabeled NAM binding affinities for the CXCR2 intracellular allosteric modulator site.

## Results and Discussion

### Fluorescent Probe Design

*R*-Navarixin (**2**) represents a prototypical CXCR2 intracellular NAM possessing high affinity and potency in functional chemotaxis assays^60^ and was deemed a suitable starting point for the development of fluorescent NAMs. Initially, using computational approaches, we sought to understand the relevant ligand–target interactions between **2** and CXCR2, to enable selection of an appropriate attachment point to incorporate a fluorophore via a suitable linker.

**Figure 2:**
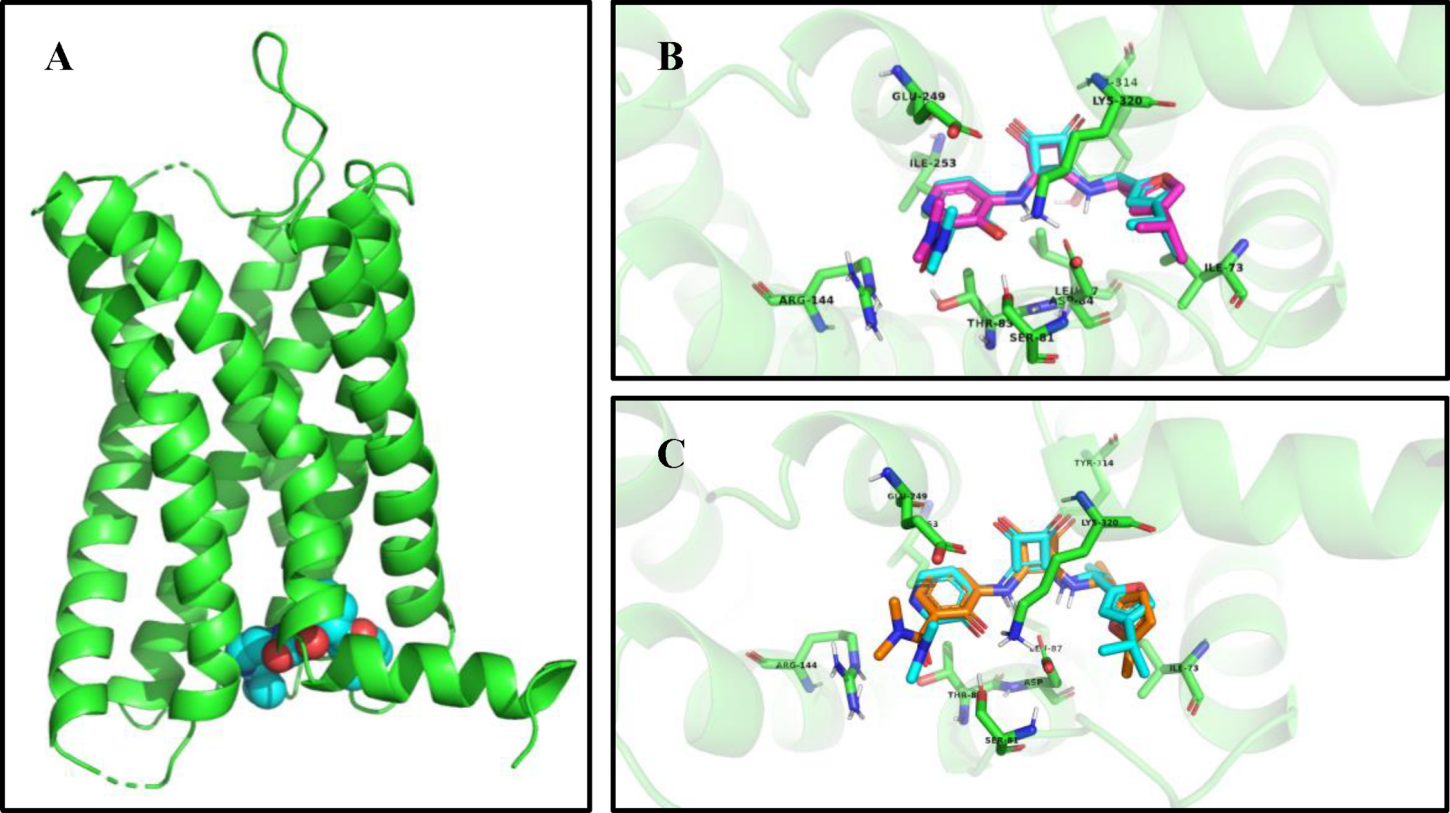
(A) Side view of CXCR2 crystal structure with 00767013 (**1**) (cyan) (PDB ID: 6LFL). (B) View from the bottom of the receptor of crystallographically determined (cyan) and redocked (magenta) poses of 00767013 (**1**) in the crystal structure of CXCR2 (PDB ID: 6LFL). (C) View from the bottom of the receptor of crystallographically determined (cyan) pose of 00767013 (**1**) and docked pose of *R*-navarixin (**2**) (orange) in the crystal structure of CXCR2 (PDB ID: 6LFL). Crucial receptor residues lining the binding pocket are shown in green.

To create a molecular model for *R*-navarixin (**2**) bound to CXCR2, we first established that using GLIDE (Schrödinger software suite release 2023-1), we could accurately redock **1** into the original CXCR2 X-ray crystal structure (PDB: 6LFL)^15^ (Figure 2B). This validated method was then used to dock *R*-navarixin (**2**) into the same ligand binding pocket.

**Figure 3:**
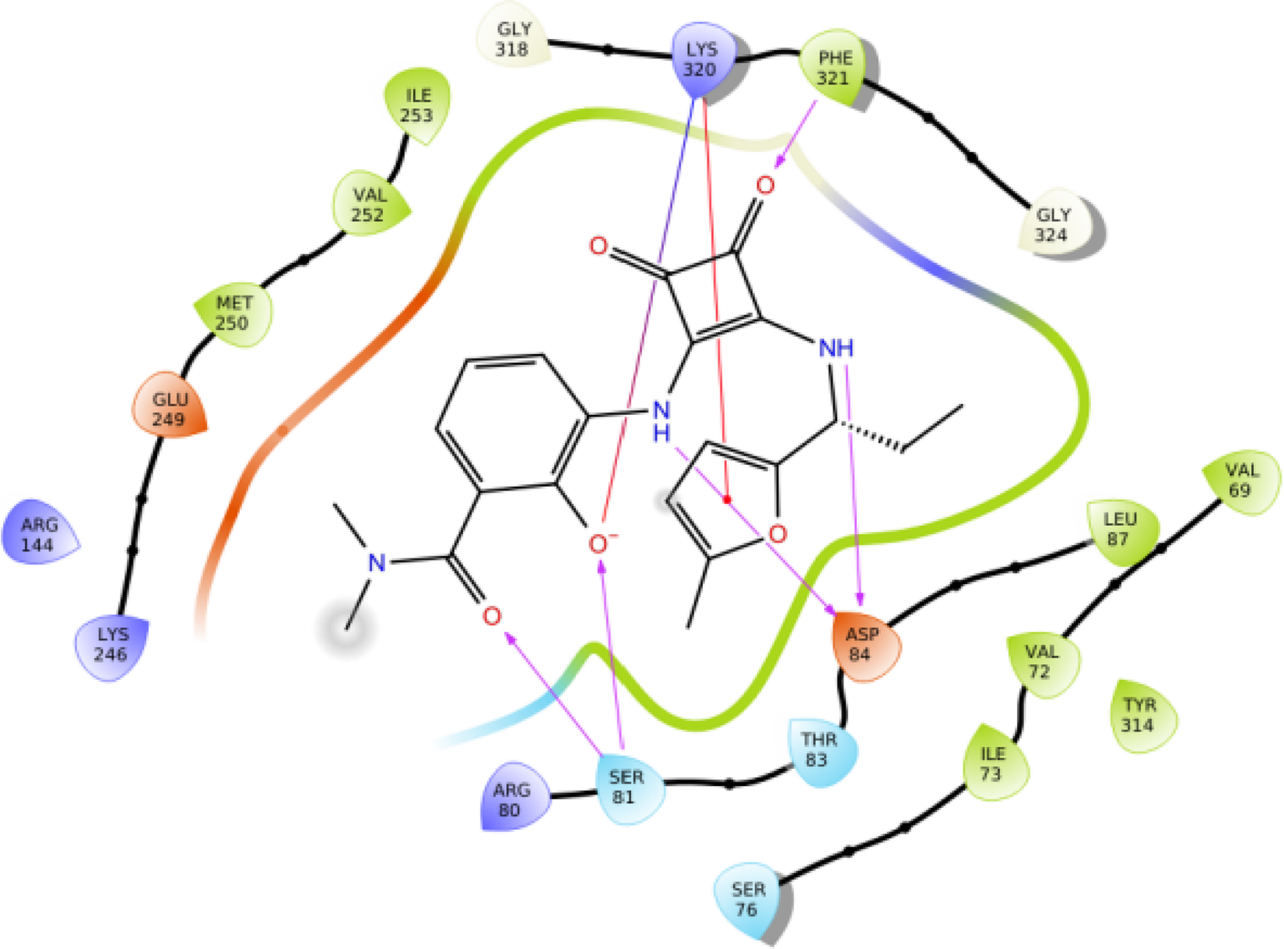
Predicted key interactions between *R*-navarixin (**2**) and CXCR2.

Given their structural similarity, it is unsurprising that the predicted binding pose of *R*-navarixin is very similar to that of 00767013 (Figures 2C and 3). The furan ring, squaramide moiety, phenol, and carbonyl oxygen of the *N*,*N*-dimethyl-amide each interact with different amino acid residues in the binding pocket including Phe321^8.50^, Lys320^8.49^, Gln319^8.48^, Arg144^3.50^, and Asp84^2.40^. The importance of these residues for NAM binding at CXCR2 has in part been supported by mutagenesis studies highlighting, for example, the relevance of Asp84^2.40^, Thr83^2.39^, and Lys320^8.49 40^. Conversely, the *N*,*N*-dimethylamide moiety does not appear to be critical for receptor binding as it is not predicted to make any direct contact with the receptor and to protrude from the pocket toward the cytosol, without establishing any crucial interactions with CXCR2.

It is necessary to note that the CXCR2 protein used to determine the CXCR2 X-ray crystal structure (PDB ID: 6LF) included mutations to facilitate crystallization,^15^ in particular, A249^6.33^E, which is in close proximity to the proposed intracellular allosteric binding pocket. Therefore, to further evaluate *R*-navarixin (**2**) binding to CXCR2, a more humanized sequence was used to generate a representative model, with Ala249^6.33^ present. The binding pose of *R*-navarixin (**2**) and its interactions with CXCR2 did not change with Ala249^6.33^ present. This additional docking confirmed that the *N*,*N*-dimethylamide moiety is not predicted to make direct interactions with the receptor and therefore was a suitable site for attachment of a linker moiety and associated fluorophore (we will show later though that in fact there is significant SAR associated with this region of the molecule).

### Fluorescent Ligand Synthesis

**Scheme 1:**
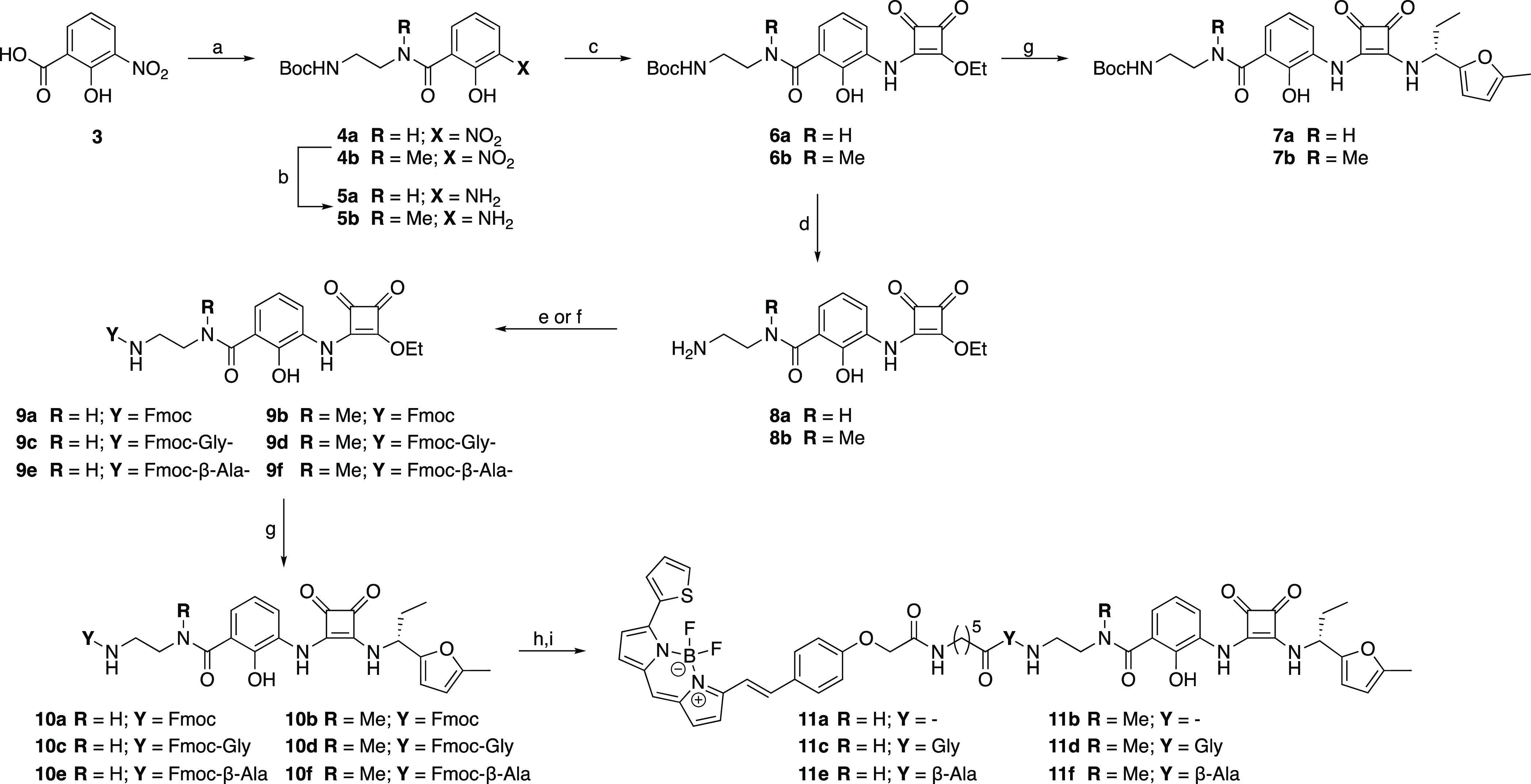
Synthesis of CXCR2 Fluorescent Probes Reagents and conditions: (a)(i) (COCl)_2_, DMF, CH_2_Cl_2_, rt; (ii) BocNH-(CH_2_)_2_-NHR, CH_2_Cl_2_, 0 °C, 50–70%; (b) 10% Pd/C, H_2_, EtOH, rt, 90%; (c) diethyl squarate, EtOH, rt, 40–70%; (d) 4 M HCl/1,4-dioxane or TFA/CH_2_Cl_2_, rt, 100%; (e) (9*H*-fluoren-9-yl)methyl (2,5-dioxopyrrolidin-1-yl) carbonate (Fmoc-OSu), *N*,*N*-diisopropylethylamine (DIPEA), CH_2_Cl_2_, 0 °C, 39–58%; (f) Fmoc-Gly-OH or Fmoc-β-Ala-OH, 1-ethyl-3-(3-dimethylaminopropyl) carbodiimide (EDCI), 1-hydroxybenzotriazole (HOBt), CH_2_Cl_2_, 0 °C, 11–42%; (g) (*R*)-1-(5-methylfuran-2-yl)propan-1-amine hydrochloride, Et_3_N EtOH, rt, 50–60%; (h) 20% piperidine/DMF; (i) BODIPY 630/650-X NHS ester, DMF, 34–69%.

*R*-Navarixin (**2**), for which the synthesis has been previously reported,^60^ was chosen as the receptor binding motif for the library of fluorescent ligands. The linkers were designed to explore both the influence of distance between the receptor binding motif and fluorophore and also the importance of the *N*,*N*-dialkylamide moiety present on **2**. As such, two focused series of linker-coupled compounds were generated through the addition of an initial *N*-(2-aminoethyl)amide or *N*-methyl-*N*-(2-aminoethyl)amide spacer (Scheme 1). The terminal primary amine functionality present on this spacer allowed either direct reaction with a suitable *N*-reactive fluorescent dye or further elongation of the linker through incorporation of a glycyl or β-alanyl moiety.

3-Nitrosalicilic acid (**3**) was converted to the amides **4a**,**b**. This was achieved first by the formation of the corresponding acyl chloride via a modified Vilsmeier–Haack reaction, which was then reacted with *tert*-butyl-*N*-(2-aminoethyl)carbamate or *tert*-butyl-(2-(methylamino)ethyl)carbamate at 0 °C. The nitro moiety of compounds **4a**,**b** was reduced, through catalytic hydrogenation over 10% Pd/C, to afford the corresponding anilines **5a**,**b**. These were then reacted with diethyl squarate in ethanol to afford the corresponding squaric acid monoamide monoesters **6a**,**b**. Compounds **6a**,**b** were treated with (*R*)-1-(5-methylfuran-2-yl)propan-1-amine to afford compounds **7a**,**b**. *N*-Boc deprotection of **6a**,**b** was achieved by treatment with either 4 M HCl/1,4-dioxane or TFA/CH_2_Cl_2_. The deprotected compounds **8a** and **8b** were either Fmoc-protected using Fmoc-OSu and DIPEA in DMF to give **9a**,**b** or coupled to Fmoc-Gly-OH or Fmoc-β-Ala-OH using EDCI, HOBt, DIPEA in CH_2_Cl_2_ to afford **9c**–**f**, respectively. Compounds **9a**–**f** were subsequently stirred at room temperature in the presence of (*R*)-1-(5-methylfuran-2-yl)propan-1-amine to afford protected congeners **10a**–**f**. Base-mediated *N*-Fmoc cleavage of **10a**,**c**–**f** in the presence of piperidine/DMF afforded the corresponding free amines, which were not isolated, but directly acylated with BODIPY 630/650-X NHS ester to afford the desired fluorescent probes **11a**,**c**–**f**.

The BODIPY 630/650 fluorescent dye was selected due to its red-emission profile, which is beneficial when imaging in living cells as it is clearly distinguishable from background cell autofluorescence emission. The BODIPY fluorophore also offers distinct advantages in terms of high quantum yield and photostability.^61^ Furthermore, the lipophilic nature of BODIPY 630/650 is beneficial in supporting membrane permeability, which is critical to targeting the cytosolic face of the receptor in a whole cell context.

Unfortunately, compound **10b** was found to be chemically unstable. Despite multiple attempts to purify and isolate **10b** using a range of approaches (normal and reverse-phase chromatography), subsequent analysis indicated that the compound lacked sufficient purity to progress. Consequently, the corresponding fluorescent probe **11b** was not synthesized.

### Pharmacological Characterization: NanoBiT Complementation Assay

**Figure 4:**
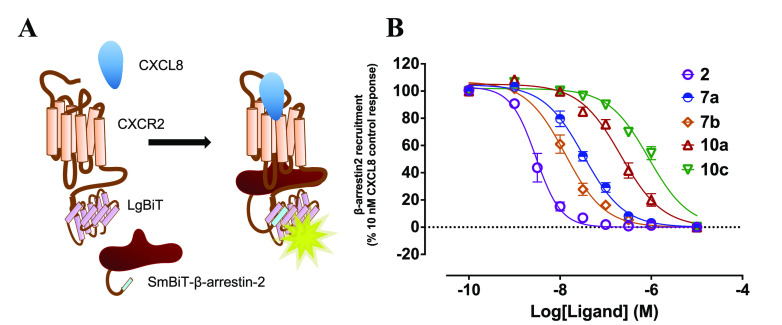
(A) NanoBit complementation assay. CXCR2 tagged with LgBiT and β-arrestin2 tagged with SmBiT of NanoLuc luciferase. Stimulation of the receptor with CXCL8 results in β-arrestin2 recruitment, enzyme complementation and luminescence generation in the presence of furimazine as enzyme substrate. (B) Concentration inhibition curves for compounds **2**, **7a**,**b**, **10a**, and **10c**, demonstrating the effect on 10 nM CXCL8 responses in the CXCR2-β-arrestin2 NanoBiT assay. Cells were pretreated for 30 min with test compounds followed by 60 min CXCL8 stimulation. The data shown are pooled from three individual experiments (mean ± SEM, *n* = 3), with each experiment performed in technical duplicate.

In order to determine any functional effect as a result of linker addition, the activity and suitability of protected congeners **7a**,**b**, **10a**, and **10c** underwent evaluation using a NanoBiT complementation assay, measuring CXCL8 stimulated recruitment of β-arrestin2 to the human CXCR2 receptor.^62^ In this assay, receptor-arrestin interaction is detected by the proximity complementation of Large BiT (LgBiT) and Small BiT (SmBiT) tags, which regenerate functional nanoluciferase in a reversible manner. This complementation is detected by real-time luminescence measurements following addition of the substrate furimazine (Figure 4A).^62^ To assess the initial pharmacological activity of the synthesized protected congeners, HEK293 CXCR2/β-arrestin2 NanoBiT cell lines were pre-treated with **2**, **7a**,**b**, **10a**, or **10c** in a concentration-dependent manner (10 μM–0.1 nM) for 30 min prior to furimazine addition and CXCL8 stimulation. Luminescence representing arrestin recruitment by the CXCR2 receptor was recorded for up to 60 min after agonist activation, with data at 60 min being presented in Figure 4B and pIC_50_ inhibitory potencies summarized in Table 1.

**Table 1:** Functional Inhibitory Potencies in the CXCR2 NanoBiT Assay (pIC_50_) for Protected Congeners (**7a**,**b**, **10a**, **10c**) Compared to *R*-Navarixin (**2**)

compound	pIC_50_ ± SEM^a^
**2**	8.54 ± 0.04
**7a**	7.54 ± 0.10
**7b**	7.93 ± 0.11
**10a**	6.67 ± 0.09
**10c**	5.95 ± 0.15

a All data are mean ± SEM of individual estimates from *n* = 3 separate experiments.

*R-*Navarixin (**2**), used as the assay reference, showed the highest potency overall, in line with previously published data,^63^ with the following order of inhibitor potency for the linker congeners **7b** > **7a** > **10a** > **10c**. Compounds **7a** and **7b** differ only through the *N*-methyl status of the salicylamide moiety. Notably, the N*-*methylated analogue (**7b**) had 2.5-fold higher potency compared to the corresponding unmethylated analogue (**7a**). The influence of linker length and nature of the protecting group present on the congener can be observed when comparing **7a**, **10a,** and **10b**. In the case of **7a** (*N-*Boc protected) and **10a** (*N*-Fmoc protected), these comprise only the initial ethylenediamine spacer, whereas **10c** (*N*-Fmoc protected) additionally incorporated a glycyl linker. These analogs demonstrated an association between decreased functional potency and the larger Fmoc protecting group. Overall, linker addition to **2** via the amide moiety identified in our docking studies appeared tolerated, with potency reductions of between 3- and 300-fold in the functional NanoBiT assay. Furthermore, assessment using a whole cell assay also requires the designed NAMs to cross the plasma membrane and bind at the intracellular CXCR2 binding site. Therefore, potency differences in this assay may reflect not only an influence on CXCR2 binding affinity, but different physicochemical properties of the linker-coupled compounds that influence their cellular permeabilities.

### NanoBRET Binding Assays

The binding affinities of the fluorescent probes **11a**, **11c**–**f** at CXCR1/2 were evaluated by generating a bioluminescence resonance energy transfer (NanoBRET) assay performed both in membranes and whole cells.

**Figure 5:**
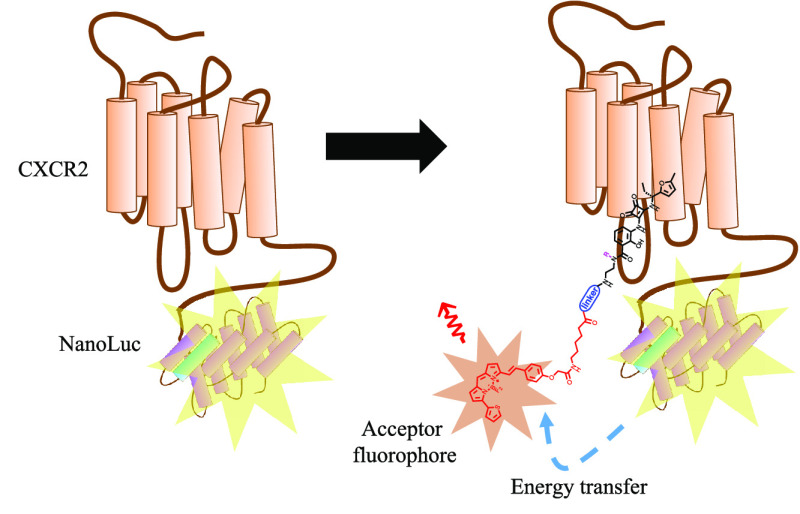
Representation of the NanoBRET binding assay. BRET occurs upon co-localization of the Nanoluciferase and fluorescent ligand, allowing dual readout of luminescence and fluorescence to generate a BRET ratio.

As shown in Figure 5, in our assay, HEK 293 cells expressed CXCR2 fused with full length NanoLuc at the intracellular *C*-terminus. The NanoLuc is the donor bioluminescent enzyme from which the energy transfer occurs in the presence of membrane permeant furimazine,^64^ and the fluorescent ligand is the acceptor fluorophore. Since the energy transfer only occurs when there is close proximity (<10 nm) between the donor and the acceptor,^64^ it is possible to monitor specific fluorescent ligand binding to the receptor of interest through the increase in BRET ratio (acceptor emission 630 nm/donor emission 460 nm) in real time, without removal of the free ligand. The BRET ratio is the ratio between the energy emitted by the acceptor fluorophore over the energy emitted by the enzyme donor.^64^

**Figure 6:**
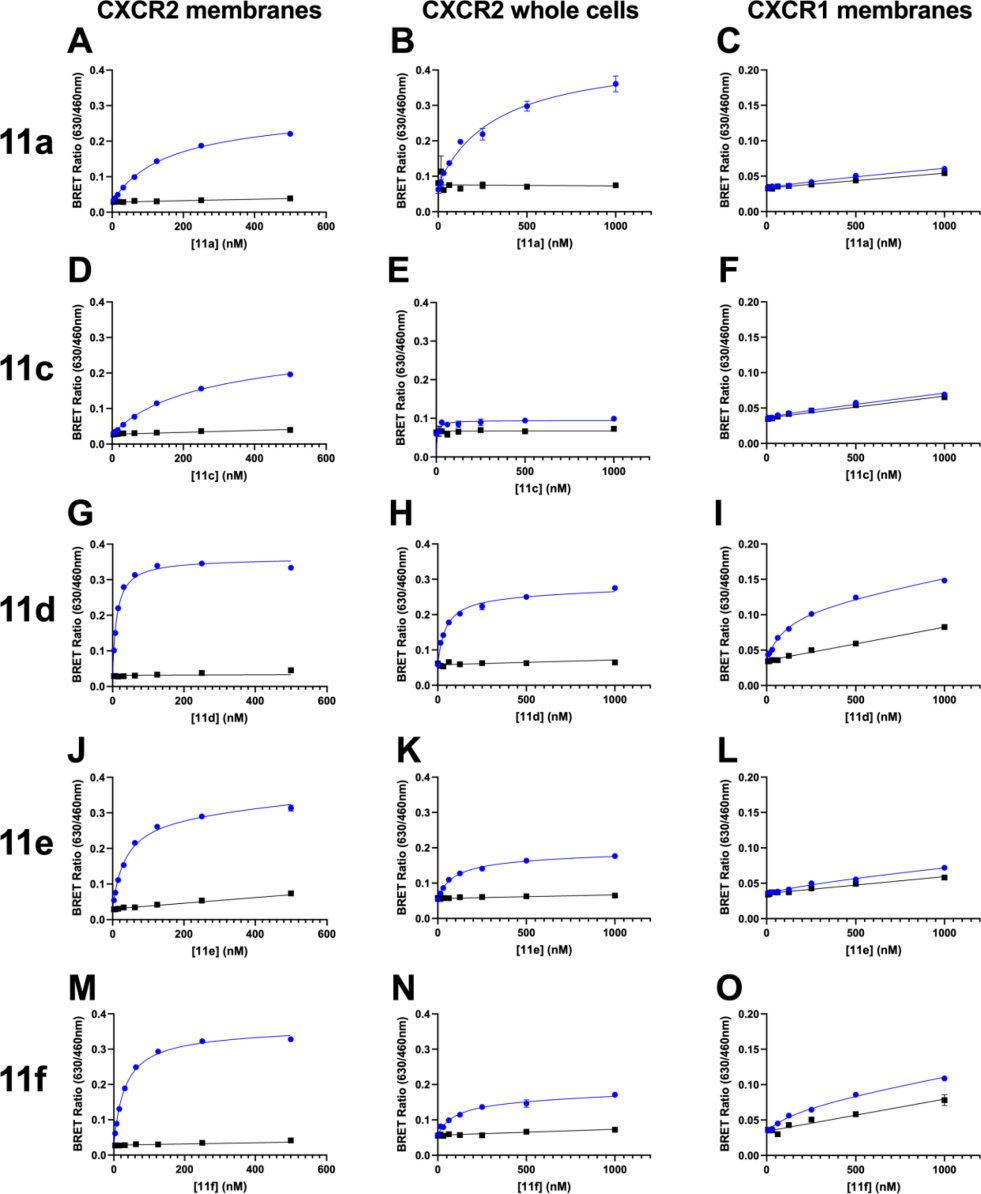
Fluorescent ligands (**11a**, **11c**–**f**) NanoBRET saturation binding studies in CXCR2 membranes (left column, A, D, G, J, M), CXCR2 whole cells (central column, B, E, H, K, N), and CXCR1 membranes (right column, C, F, I, L, O) in the absence (blue) or presence (black) of 100 nM or 10 μM *R*-Navarixin (**2**) used to measure non-specific binding (NSB). Data are representative experiments from *n* = 5 individual experiments performed in duplicate. The equivalent graphs showing specific binding curves can be found in Figure S24.

Fluorescent ligand binding (**11a**, **11c**–**f**) was initially tested in a cell free NanoBRET assay using HEK-CXCR2-NanoLuc membranes (Figure 6A,D,G,J,M). All the ligands showed saturable binding and low non-specific binding (NSB, determined with the addition of a high concentration of unlabeled ligand, **2**), as determined by the competition of **2** for the intracellular allosteric binding site. The highest CXCR2 binding affinities were obtained with the N-methylated ligands **11d** and **11f** (*K*_D_ of 10–27 nM; Table 2). The influence of linker composition was also evident in the comparison of non-methylated analogue affinities, with the order of *K*_D_ for **11e > 11a** > **11c** (Table 2). Additionally, the use of fluorescent ligands within a homogeneous real-time NanoBRET system facilitated characterization of ligand binding kinetics, exemplified through use of tracer **11a**, allowing estimation of the association rate constant *k*_on_ (1.9 ± 0.2 × 10^5^ M^–1^ min^–1^), dissociation rate constant *k*_off_ (0.013 ± 0.005 min^–1^), and a kinetically derived p*K*_D_ of 7.3 ± 0.2 (*n* = 4) of compound **11a** in line with endpoint derived parameters.

**Table 2:** Binding Affinities of the Fluorescent Ligands in CXCR2 Membranes, CXCR2 Whole Cells, and CXCR1 Membranes Determined by Saturation Binding Assays

	*K*_D_ ± SEM^a^ (nM)		
compound	CXCR2 membranes	CXCR2 whole cells	CXCR1 membranes
**11a**	137 ± 12	549 ± 83	
**11c**	301 ± 31		
**11d**	9.0 ± 4.9	52 ± 7	101 ± 21
**11e**	45 ± 4	82 ± 14	
**11f**	27 ± 3	88 ± 23	372 ± 107

a All values represent mean ± SEM of *n* = 5 separate experiments.

**Figure 7:**
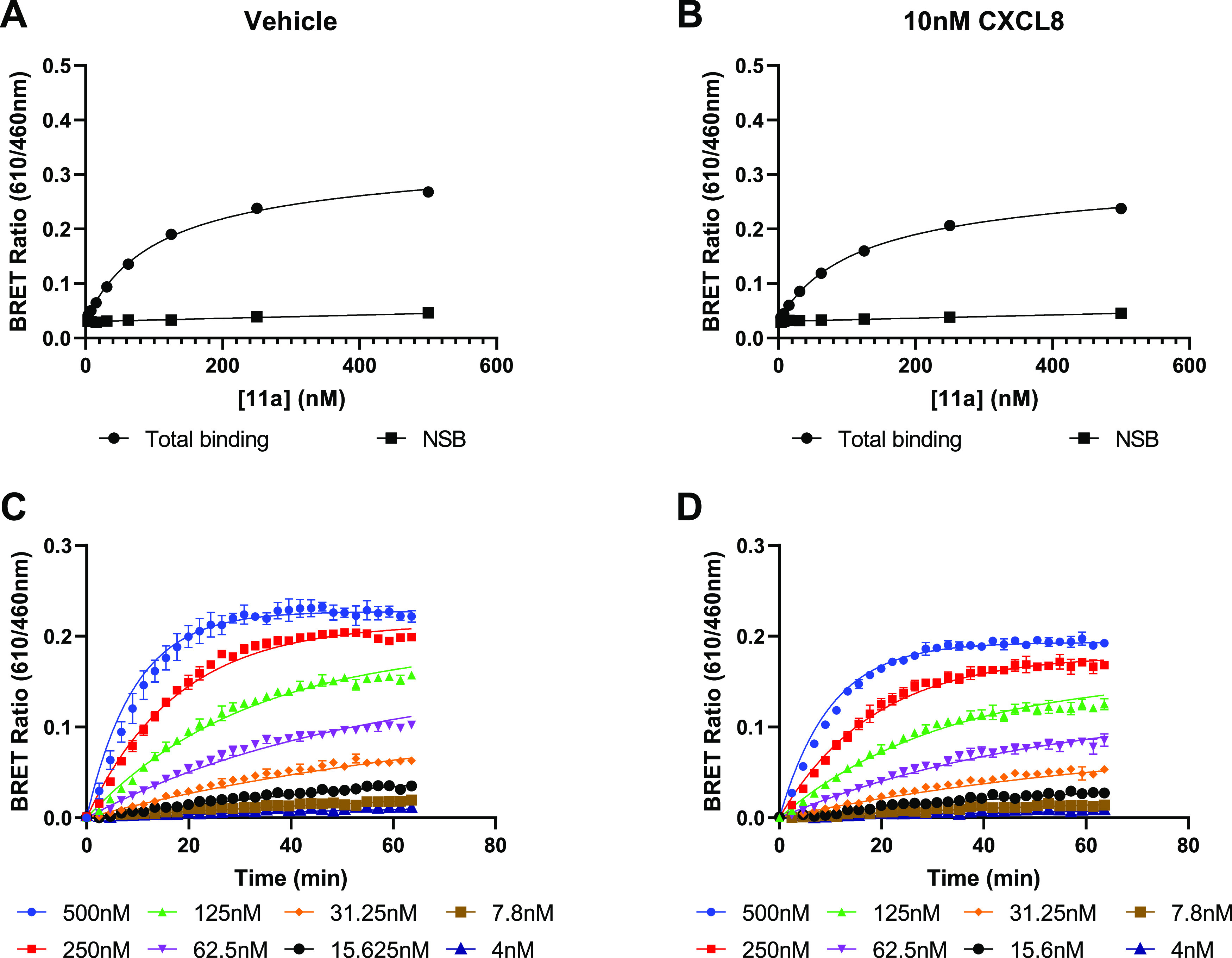
Saturation binding experiments employing endpoint and kinetic analysis for derivation of ligand affinity. **11a** NanoBRET saturation binding and association kinetic studies in CXCR2 membranes in the absence of CXCL8 (A, C) and in the presence of 10 nM CXCL8 (B, D). Data are representative experiments from 4 performed.

Furthermore, saturation binding experiments were carried out in the presence of a saturating concentration of agonist CXCL8 (IL8) (Figure 7). Under agonist conditions the affinity and kinetic parameters of **11a** displayed no significant difference to that determined in the absence of CXCL8 (**11a** + 10 nM CXCL8, *K*_D_ = 149.2 ± 16 nM, p = 0.45, n = 4, Student’s paired *t* test; *k*_on_ = 1.7 ± 0.2 × 10^5^ M^–1^ min^–1^, *k*_off_ = 0.014 ± 0.005 min^–1^, *n* = 4).

**Figure 8:**
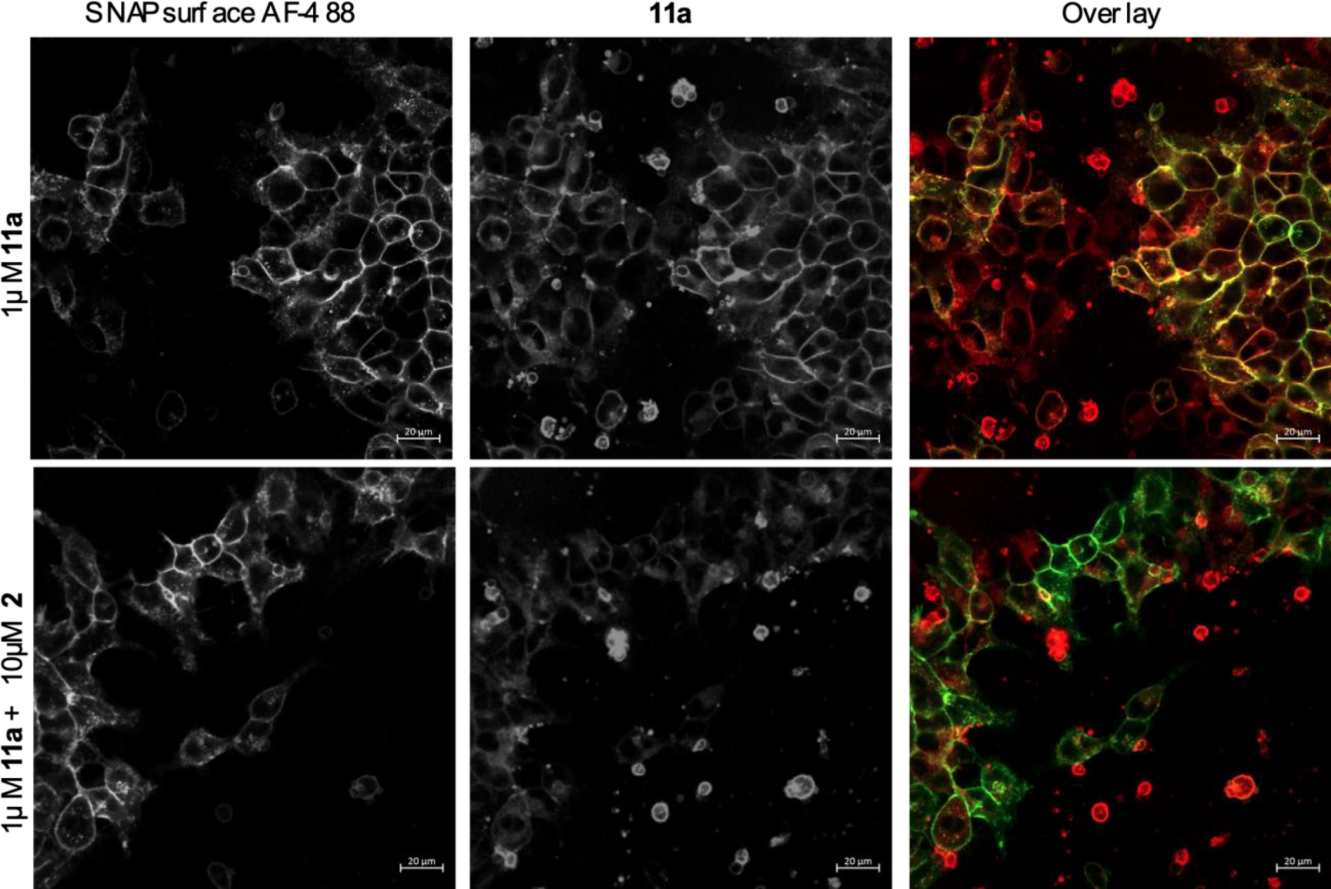
Live cell imaging of **11a** binding to SNAP-tagged CXCR2-tsNanoLuc HEK293. Cells were pre-labeled with SNAPsurface-AF488 to identify the SNAP-tagged receptors (green), prior to incubation of **11a** (red) in the absence or presence of 10 μM **2** to define non-specific binding (10 min at 37 °C), prior to fluorescence imaging. Scale indicates 20 μm.

Having determined fluorescent ligand binding affinities in membranes, we sought to employ these ligands within a cellular setting. To assess the influence of cellular permeability on compound binding, **11a**, **11c**–**f** were tested in the whole cell format of the CXCR2 NanoBRET assay (Figure 6B,E,H,K,N). We were able to measure saturable binding and determine *K*_D_ values for all compounds except for **11c**, with the same rank order of affinity and a drop in apparent *K*_D_ of 1.8–5.8-fold. The N*-*methylated fluorescent ligands **11d**–**f** and compound **11e** were again the highest affinity in the whole cell system, with *K*_D_ values of 52–88 nM. These experiments demonstrate the applicability of these fluorescent probes in both membrane- and cell-based CXCR2 binding assays. Moreover, we have confirmed cell accessibility of the probes by direct fluorescent imaging of the CXCR2 cell line, co-labeled with the SNAP-tag receptor fluorophore and **11a**. These data demonstrate both cell surface and intracellular endosomal fluorescent labelling of the cells by **11a**, in a specific manner with competitive displacement by a high concentration of unlabeled **2** (Figure 8).

We also assessed the selectivity of probes **11a**, **11c**–**f** for the related chemokine receptor CXCR1 compared to CXCR2, given the reported 100-fold CXCR2:CXCR1 functional selectivity for the parent NAM *R*-navarixin (**2**).^13^ In the CXCR1-NanoLuc NanoBRET binding assay in membranes, **11a**, **11c**, and **11e** showed no CXCR1 specific binding (Figure 6C,F,I, L,O; Table 2). However, saturable CXCR1 binding was observed for **11d** and **11f**, with, respectively, 11-fold and 14-fold reductions in affinity at CXCR1 compared to CXCR2 (Table 2). These data demonstrate that the selectivity of the fluorescent probes for CXCR2 over CXCR1 is retained, albeit to a lesser extent than the parent compound. However, **11d** and **11f** possess sufficient affinity to be used in a CXCR1 NanoBRET assay probing for the corresponding intracellular binding site.

**Figure 9:**
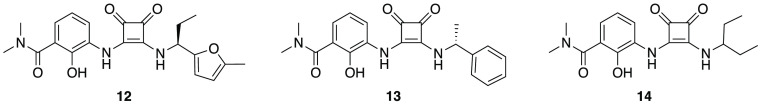
Structures of known CXCR2 NAMs tested.

**Figure 10:**
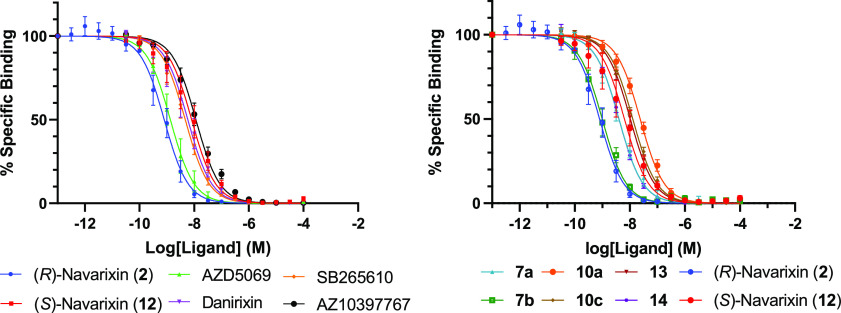
NanoBRET competition binding studies CXCR2 allosteric modulators in CXCR2 membranes. Membranes were incubated with 100 nM **11a** and increasing concentrations of unlabeled ligands for 3 h at 37 °C. The data shown represent the combined mean ± SEM of *n* = 5 experiments where each experiment was performed in duplicate.

We performed NanoBRET competition binding experiments in membranes from HEK293 CXCR2-NanoLuc cells, employing **11a** as the probe and a variety of competing CXCR2 NAMs, including both known literature compounds and previously presented protected congeners (Figure 10).^13,35,47,60,65,66^ Unlabeled NAMs fully displaced the fluorescent ligand in a competitive manner, enabling the calculation of p*K*_i_ values (see Table 3) through application of Cheng–Prusoff correction. These estimates were in good agreement with previously reported CXCR2 affinity measurements for literature compounds^13,35,47,60,65,66^ as well as the NanoBiT assay potencies established for navarixin (**2**) and its protected congeners in Table 1.

**Table 3:** Measured Binding Affinities of CXCR2 NAMs Using the **11a** NanoBRET Assay and Literature Values^13,35,60,65,66^

compound	p*K*_i_ ± SEM^a^	literature p*K*_D_
*R*-Navarixin (**2**)	9.19 ± 0.17	10.3^13,60^
**7a**	8.16 ± 0.16	
**7b**	8.87 ± 0.06	
**10a**	7.62 ± 0.05	
**10c**	7.97 ± 0.04	
*S*-navarixin (**12**, Figure 9)	8.31 ± 0.28	
**13** (Figure 9)	8.06 ± 0.08	
**14** (Figure 9)	8.48 ± 0.06	
SB265610	8.68 ± 0.21	8.5^35^
AZD5069	9.61 ± 0.33	9.4^65^
danirixin	8.71 ± 0.12	8.2^66^
AZ10397767	8.32 ± 0.12	8.7^65^^b^

a All values represent mean ± SEM of *n* = 5 separate experiments.

b pA_2_ parameters derived from functional data.

Furthermore, we performed NanoBRET competition binding experiments in membranes derived from a HEK293 CXCR1-NanoLuc cell line, employing **11d** and two competing CXCR1/2 NAMs, *R-* and *S-*navarixin (**2** and **12**, Figure S1). Similarly, NAMs displayed full displacement of the fluorescent ligand in a competitive manner at CXCR1 and therefore we were able to calculate p*K*_i_ values through application of the Cheng–Prusoff correction as previously described. Our data corroborate previous findings, indicating that both enantiomers display reduced affinity for CXCR1 (*R*-navarixin (**2**), p*K*_i_ = 7.66 ± 0.15; *S*-navarixin (**12**), p*K*_i_ = 5.59 ± 0.18).

### Conformational Analysis of Methylated and Unmethylated Ligands

**Figure 11:**
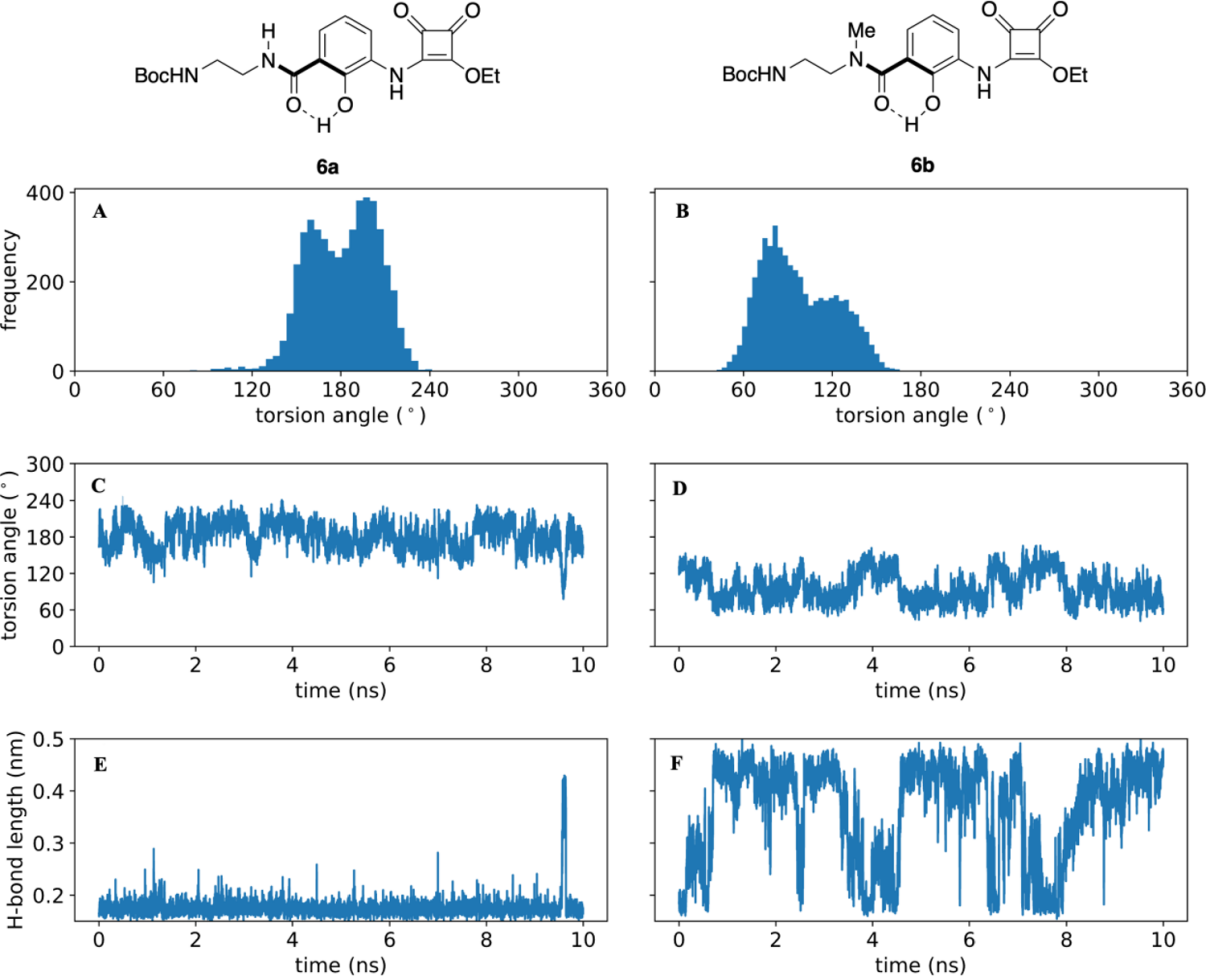
Conformational analysis of molecular dynamics simulations of **6a** and **6b** in DMSO. (A, B) Torsion angle distributions for the highlighted bond in **6a** and **6b**, respectively. (C, D) Time courses for the selected torsion angle. In **6a**, the angle rapidly and repeatedly passes through the planar (180°) conformation, while for **6b**, it is restricted (atropisomerism, at least on this time scale). (E, F) Time courses for the distance between the carbonyl oxygen and phenolic hydrogen atoms in **6a** and **6b**, respectively). In **6a**, a strong H-bond is maintained, while in **6b**, it is present or absent depending on whether the high or low twist conformation of the torsion angle is adopted.

A recurrent observation in the pharmacological data is the enhanced potency of N-methylated ligands over their unmethylated congeners (e.g., **11d** vs **11c** and **11f** vs **11e**). This finding could not be predicted from the original docking studies on which the ligand designs were based as they suggested that the *N*,*N*-dimethylamide portion of *R*-navarixin (**2**) is not involved in significant interactions with the receptor. Therefore, to rationalize the pharmacological data, we performed some additional studies with a particular focus on the *N*-methylamide portion of the congeners analyzing some intermediates made during the synthesis of the fluorescent probes. We initially chose compounds **7a** and **7b** as they include a short *N*-Boc protected ethylenediamine linker, more convenient for docking purposes being not excessively long and flexible, with or without N-methylation of the amide. Moreover, these two analogues showed a notable difference in potency when tested in the previously described NanoBiT assay (**7b** > **7a**). We docked them in CXCR2 crystal structure (PDB:6LFL) humanized with Ala249 following the methodology previously described. The binding poses of both compounds mimic that for *R*-navarixin and no significant difference was shown in the interaction with the receptor or in docking score between the N-methylated (**7b**) and unmethylated (**7a**) analogues. Since docking studies did not provide sufficient data, which could explain the difference in potency between the analogues, we decided to focus on their potential difference in structural conformation. Initial evidence for this arose from the observation of broadened signals in the ^1^H-NMR spectra of the methylated compounds with respect to the corresponding unmethylated analogues, suggesting that methylation affects the conformational preference of the ligands. To perform more detailed structural NMR studies, we selected two previous intermediates in the synthesis, compounds **6a** and **6b**. These can be considered as “minimal ligands” as they retain either a methylated or unmethylated amide moiety, the phenol and a mixed squarate moiety, with the chiral furanylalkylamino moiety, replaced with an ethoxy group. The lack of chirality of analogues **6a** and **6b** facilitated more straightforward acquisition and analysis of ^1^H-NMR spectra with a focus on the conformational nature of the benzamide region. First, ^1^H-NMR spectra for each compound in DMSO were fully assigned using two-dimensional NMR spectroscopy experiments (correlation spectroscopy (COSY), heteronuclear single-quantum correlation spectroscopy (HSQC), and heteronuclear multiple-bond correlation spectroscopy (HMBC)). Subsequently, to obtain structural information, we employed rotating-frame nuclear Overhauser effect spectroscopy (ROESY) (Figures S20 and S21). This technique, used to establish through-space correlation between nuclei physically near to each other,^67,68^ allowed us to visualize the interactions of the protons in the molecule that are close in space even if they are not bonded or coupling to each other. Interestingly, compounds **6a** and **6b** differ particularly in their phenolic OH signal. Notably, the phenolic OH peak is shifted downfield in the unmethylated compound **6a** with respect to the N-methylated analogue **6b** (13.5 and 9.4 ppm, respectively). Moreover, for the phenolic OH of compound **6a**, several through-space interactions with other protons of the molecule, such as the benzamide NH and adjacent CH_2_ protons of the ethylene region and the aromatic protons, were detected. Conversely, no through-space interactions were detected for the phenolic OH in the N-methylated compound **6b**. These data suggest that the *N*-methyl group could introduce a conformational restriction in compound **6b** (and thus other *N*,*N*-dialkylamide analogues), preventing intramolecular hydrogen bonding between the phenol and benzamide moieties, whereas the absence of the *N*-methyl group allows the above intramolecular hydrogen bond to form making phenol less available for receptor interaction. Furthermore, the need to disrupt the intramolecular hydrogen bond would confer an energetic penalty. This could explain the difference in potency between unmethylated and methylated analogues, as previous studies^69^ suggest that the interaction between the phenol and the receptor is crucial. Additionally, the rotational restriction present in the N-methylated analogue **6b** could promote a conformation that favors receptor binding. Alongside the NMR experiments, we performed molecular dynamics simulations of **6a** and **6b** in DMSO. These predict that the key amide bond maintains a strict *trans*-geometry in both molecules but that the neighboring bond connecting to the phenolic ring (torsion angle highlighted in Figure 11) behaves very differently in **6b** compared to **6a**. In **6a**, we observe that the torsion angle distribution (Figure 11A) shows a bifurcated maximum ∼20° either side of 180°, with a low barrier between the two states that is crossed rapidly and repeatedly over the 10 ns simulation (Figure 11C). Throughout this time, a hydrogen bond between the amide oxygen and phenolic OH is highly conserved (Figure 11E). In contrast, for **6b**, we observe that the torsion angle distribution (Figure 11B) shows a bimodal distribution with maxima at 80° and 120°. Transitions between the two states are rapid and frequent (Figure 11D) and correlate with the formation (120°) and breakage (80°) of the hydrogen bond with the phenolic OH (Figure 11F). There is a symmetry-related conformation when the torsion angle lies in the −80° to −120° range. This can be generated by the application of torsion angle restraints to the simulation to drive it through the planar state, but the increased steric hinderance provided by the *N*-methyl group means that, in contrast to **6a**, we observe no spontaneous transitions, at least on the 10 ns time scale (results not shown).

The simulations thus are in full agreement with the NMR analysis, supporting the hypothesis that a considerable portion of the enhanced activity of N-methylated analogues could come from the effects that this modification has on the structure of the free ligand: methylation favors a less planar conformation and a weaker intramolecular hydrogen bond, both of which would be expected to contribute to a more favorable binding free energy.

## Conclusions

Intracellular allosteric modulators are novel alternatives for the selective targeting of chemokine receptors, which are important drug targets across multiple immunological diseases, respiratory disorders, and cancers, but present challenges for developing traditional orthosteric directed small molecules. Nonetheless, our understanding of how these NAMs bind and modulate receptor pharmacology could be improved significantly by tools that enable direct interrogation of the binding site at the receptor–effector interface. Here, we demonstrate structure guided design and synthesis of fluorescent ligands (**11a**, **11c**–**f**) based on the CXCR2-selective NAM *R*-navarixin (**2**) and show their application to the study of CXCR2 and CXCR1 NAM binding and function, through both fluorescence imaging and real-time resonance energy transfer assays applicable to medium throughput drug discovery.

Pharmacological assessment of the suite of synthesized fluorescent ligands in this study highlighted the importance of linker composition for activity and CXCR1/CXCR2 selectivity, as documented elsewhere in the synthesis of other GPCR fluorescent ligands.^52,70^ For example, β-alanyl incorporation on to the *N*-(2-aminoethyl)amide linker improved CXCR2 affinity compared to glycyl incorporation or an unextended *N*-(2-aminoethyl)amide linker. The presence of an *N*-methyl-*N*-(2-aminoethyl)amide also improved affinity resulting in a less planar conformation and weaker intramolecular hydrogen bonding. Notably, the availability of probes with a range of affinities is beneficial in terms of fluorescent ligand assay development to determine GPCR ligand binding. For example, the availability of fluorescent probes with fast kinetics (and lower affinity) extends the range and accuracy of the determination of unlabeled ligand kinetic parameters in competition kinetic assay development using BRET assays.^71^

Given the intracellular nature of the CXCR2 modulator binding site, membrane permeability of the designed fluorescent probes is also a key consideration since this allows the use of in cell target engagement binding assays, with the receptors in a native context, in addition to cell free membrane systems. We demonstrated that compounds **11a** and **11d**–**f** were indeed able to cross the cell membrane and reach their intracellular binding site, showing similar orders of affinity to previous data, displaying their potential utility in future cell-based assays. An influence of membrane permeability and reduced intracellular concentration, together with the whole cell receptor context, may account for the 4–6-fold reduced probe binding affinity observed in cells compared to membranes.

CXCR1 and CXCR2 possess high sequence homology, and it is known that the congener of our fluorescent probes, *R*-navarixin (**2**), binds both receptors, showing 34-fold lower potency at CXCR1. Within the SAR, we observed that our fluorescent ligands retained CXCR2 selectivity but to differing degrees. For example, **11d** bound CXCR1 with suitably high affinity (*K*_D_ ∼ 100 nM) to be considered as a fluorescent tracer in binding assays exploring NAM receptor pharmacology for this receptor subtype as well as CXCR2.

Finally, we demonstrated direct determination of the affinities of a range of structurally distinct CXCR2 NAMs using **11a** as the fluorescent tracer in a membrane-based NanoBRET competition assay, using a real-time homogeneous format. These data correlate well with both published data^13,35,60,65,66^ and our own assessments of the functional activity of these NAMs on CXCR2 signaling. Moreover, we demonstrate that these ligands can be used in ligand binding kinetic studies and could be employed in future competition kinetic studies to determine the properties of unlabeled CXCR2 NAMs. In conclusion, our synthesized fluorescent ligands constitute a novel toolbox for elucidation of the pharmacology of current and novel small molecule NAMs at the CXCR2 and CXCR1 receptors.

## Experimental Section

### General Chemistry

Chemicals and solvents were purchased from standard suppliers and used without further purification. BODIPY 630/650-X NHS was purchased from Lumiprobe (Hunt Valley, MD). Compounds **2** (*R*-navarixin), **12** (*S*-navarixin), **13**, and **14** were synthesized according to the previously reported procedure from Dwyer et al.,^60^ and all the NMR data obtained were in accordance with reported literature data. Unless otherwise stated, reactions were carried out at ambient temperature and monitored by thin layer chromatography on commercially available precoated aluminum-backed plates (Merck Kieselgel TLC Silica gel 60 Å F_254_). Visualization was by examination under UV light (254 and 366 nm) followed by staining with ninhydrin. Organic solvents were evaporated under reduced pressure at ≤40 °C (water bath temperature). Flash column chromatography was carried out using technical-grade silica gel from Aldrich, pore size 60 Å, 230–400 mesh particles size and particle size 40–63 μm. Preparative layer chromatography (PTLC) was performed using precoated glass plates (Analtech uniplate silica gel GF, 20 × 20 cm, 2000 μm). Analytical RP-HPLC was performed using YMC-Pack C8 column (150 mm × 4.6 mm × 5 μm) at a flow rate of 1.0 mL/min over a 30 min period (gradient method of 10%–90% solvent B; solvent A = 0.01% formic acid in H_2_O, solvent B = 0.01% formic acid in CH_3_CN), and UV detection at 254 nm and spectra were analyzed using Millennium 32 software. ^1^H NMR and ^13^C NMR spectra were recorded on a Bruker-AV 400, respectively, at 400.13 MHz and at 101.62 MHz. Chemical shifts (δ) are quoted in parts per million (ppm) with calibrated to the residual undeuterated solvent signal. Solvents used for NMR analysis were CDCl_3_ supplied by Cambridge Isotope Laboratories Inc., (δ_H_ = 7.26 ppm, δ_C_ = 77.16 ppm), DMSO-*d*_6_ supplied by Sigma Aldrich (δ_H_ = 2.50 ppm, δ_C_ = 39.52 ppm) and CD_3_OD supplied by Sigma Aldrich (δ_H_ = 3.31 ppm, δ_C_ = 49.00 ppm). The spectra were analyzed using NMR software MestReNova. Coupling constants (*J*) are recorded in Hz and the significant multiplicities described by singlet (s), doublet (d), triplet (t), quadruplet (q), broad (br), multiplet (m), and doublet of doublets (dd). LC/MS was carried out using a Phenomenex Gemini-NX C18 110 Å column (50 mm × 2 mm x 3 μm) at a flow rate 0.5 mL/min over a 5 min period (gradient method of 5%–95% solvent B; solvent A = 0.01% formic acid in H_2_O, solvent B = 0.01% formic acid in CH_3_CN). LC/MS spectra were recorded on a Shimadzu UFLCXR system combined with an Applied Biosystems API2000 electrospray ionization mass spectrometer and visualized at 254 nm (channel 1) and 220 nm (channel 2). High-resolution mass spectra (HRMS) were recorded on a Bruker microTOF mass spectrometer using electrospray ionization (ESI-TOF) operating in positive or negative ion mode. All pharmacologically tested compounds are >95% pure by HPLC analysis. Chromatographic purity traces and HRMS spectra are available in Figures S2–S19. Optical rotations were measured using a ADP200 polarimeter (Bellingham + Stanley Ltd).

### Molecular Biology, Cell Culture, and Membrane Preparation

CXCR1 (GenBank: NM_000634.3) and CXCR2 (GenBank NM_001557.3) receptor cDNA sequences were amplified via a polymerase chain reaction and cloned downstream of a SNAP-tag (New England Biolabs, Hitchin, UK) cDNA sequence, between EcoRI and XhoI sites, in the previously generated pcDNA3.1neo(+)-SNAP mammalian expression vector (Invitrogen, Paisley, UK)^72^ containing a Kozak sequence (GCCACC) and the 5-HT3 receptor signal sequence (amino acids MRLCIPQVLLALFLSMLTGPGEGSRK) upstream to facilitate membrane integration. Addition of either a LgBiT fragment^62^ or thermostable Nanoluciferase (tsNanoLuc)^73^ at the *C* terminus was achieved through cloning of corresponding sequences, in frame with receptor *C* termini, between XhoI/XbarI restriction sites, generating p3.1neo-SNAP-CXCR1/2-LgBiT and p3.1neo-SNAP-CXCR1/2-tsNanoLuc constructs. Constructs were used to generate HEK 293 stable cell lines through transfection using Lipofectamine 3000 (Invitrogen, US) in Opti-MEM media (Sigma Aldrich) with pcDNA3.1 SNAP-CXCR2-LgBit, and pcDNA3.1zeo-β-arrestin2-SmBit (GenBank NC_000017.1)^74^ for NanoBiT complementation or pcDNA3.1neo-SNAP-CXCR1/CXCR2-tsNanoLuc (HEK 293 SNAP-CXCR2-NanoLuc or SNAP-CXCR1-NanoLuc) for NanoBRET binding and imaging assays. Mixed population cell lines were selected through G418 (0.8 mg^–1^) and Zeocine (200 μg ml^–1^) resistance and maintained in DMEM supplemented with 10% fetal bovine serum (FBS; Sigma Aldrich).

For NanoBRET assays, cells were allowed to grow to 90% confluency in T175cm^2^ flasks prior to membrane preparation. Cells were washed twice with phosphate-buffered saline (PBS, Sigma-Aldrich, Pool, UK) to remove growth medium and removed from the flask by scraping in 10 mL of PBS. Cells were pelleted by centrifugation (10 min, 2000 rpm) prior to freezing at −80 °C. For membrane homogenization (all steps at 4 °C), 20 mL of wash buffer (10 mM HEPES, 10 mM EDTA, pH: 7.4) was added to the pellet before disruption (8 bursts) with an Ultra-Turrax homogenizer (Ika-Werk GmbH & Co. KG, Staufen, Germany) and centrifugation at 48000*g* at 4 °C. The supernatant was removed, and the pellet was resuspended in 20 mL of wash buffer and centrifuged again as above. The final pellet was suspended in cold 10 mM HEPES with 0.1 mM EDTA (pH 7.4). Protein concentration was determined using the bicinchoninic acid assay kit (Sigma-Aldrich, Pool, UK) using bovine serum albumin as standard, and aliquots were maintained at −80 °C until required.

### NanoBiT Complementation Assays

Stable transfected HEK 293 cells co-expressing SNAP-CXCR2-LgBit and β-arrestin2-SmBit were seeded on white, clear bottom, poly-d-lysine-coated 96-well plates (Greiner 655098) at a density of 32,000 cells/well and allowed to grow overnight. NanoBiT assay buffer consisted of Hepes balanced salt solution (147 mM NaCl, 24 mM KCl, 1.3 mM CaCl_2_, 1 mM MgSO_4_, 1 mM Na pyruvate, 1 mM NaHCO_3_, 10 mM HEPES, pH 7.4) with 0.1% Bovine serum albumin and 10 mM d-Glucose. Cells were washed with assay buffer to remove growth media prior to incubation with respective ligand concentrations (or *R*-navarixin (**2**) control) for 1 h. Chosen ligands were first diluted in DMSO to 10 mM and stored at −20 °C prior to use. Ligands were diluted in assay buffer to required concentrations (final assay concentration range: 10 μM–0.1 nM) prior to addition to an assay plate. Post-incubation, a furimazine substrate (1/660 dilution in assay buffer from supplier stocks) was added to cells and allowed to equilibrate for 5 min at 37 °C. Upon equilibration, initial baseline luminescence readings were taken prior to the addition of 10 nM CXCL8 (aa28–99) (Stratech Scientific, Ely, UK). Luminescence readings were continually monitored over a 60 min time course every 15 min at 37 °C post-agonist addition using a BMG PHERAstar FS (BMG Labtech). Ligand IC_50_ values were obtained using a four-parameter logistic equation.

### NanoBRET Fluorescent Ligand Binding Assay

NanoBRET assays were carried out in OptiPlate-384 white well microplates (product number: 6007290, PerkinElmer LAS Ltd., UK) and used 25 mM HEPES, 1% DMSO, 0.1 mg/mL Saponin, 0.2 mg Pluronic acid F_127_, 1 mM MgCl_2_ and 0.1% BSA (pH 7.4) assay buffer. Both saturation and competition assays employed 1 μg/well HEK 293 SNAP-CXCR2-NanoLuc or SNAP-CXCR1-NanoLuc cell membranes for characterization of fluorescent ligand binding and employed 100 nM or 10 μM *R*-navarixin (**2**) to define non-specific binding (NSB). For saturation binding experiments, used to determine fluorescent ligand affinity, membranes were incubated with increasing concentrations of fluorescent ligand (8–1000 nM dilution range in assay buffer) and either assay buffer or NSB, with or without 10 nM CXCL8 28–99 (final assay volume, 40 μL). Membranes were incubated with furimazine at a 1/660 dilution for 5 min prior to addition to the assay plate, allowing for equilibration of luminescence output. NanoBRET was monitored every 15 s for 60 min at 37 °C measuring Nanoluciferase output (450 nm) and BODIPY 630–650 output (630 nm [610-LP filter]), generating a BRET ratio (630 nm/450 nm), using a BMG PHERAstar FS (BMG Labtech). Collected data was converted to specific binding measurements through subtraction of NSB data and analyzed by endpoint saturation analysis, allowing determination of ligand dissociation constant (*K*_D_) through
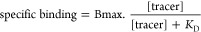
1

Additionally, specific binding traces for **11a** (defined as total binding – NSB) were fitted to a one site association model. Global fitting of this model across multiple fluorescent ligand concentrations from the same experiment enabled estimation of ligand association (*k*_on_) and dissociation rate constants (*k*_off_), together with the kinetically derived *K*_D_ (= *k*_off_/*k*_on_) using the equations:
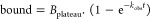
2where *B*_plateau_ is the equilibrium level of tracer binding, and the observed association rate constant *k*_obs_ is related to the binding rate constants for tracer in a single site model by
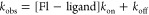
3

For competition binding assays, HEK 293 SNAP-CXCR1/CXCR2-tsNanoLuc cell membranes were incubated with 100 nM fluorescent ligand, ranging concentrations of unlabeled ligands or NSB/vehicle controls, and 1/660 furimazine (final assay volume, 30 μL). Membranes were added to the assay plate, after 5 min incubation with furimazine, by online injection using the BMG PHERAstar FS injector. NanoBRET measurements were taken over 3 h at 37 °C. Data was normalized using NSB to define 0% and vehicle controls to define 100% binding and was fit to a three-parameter logistic equation to determine unlabeled ligand IC_50_ estimates using
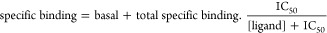
4

IC_50_ values were further converted to competing ligand dissociation constants (*K*_i_) values using the Cheng–Prusoff correction:
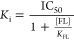
5where *K*_FL_ and [FL] represent the fluorescent ligand dissociation constant and concentration, respectively.

Cellular based binding assays were carried out in white, clear bottom, 96-well Greiner plates (655098, Greiner Bio-One, Stonehouse, UK). HEK 293 SNAP-CXCR2-NanoLuc cells were seeded at 32,000 cells/well, and assay buffer was Hepes balanced salt solution (147 mM NaCl, 24 mM KCl, 1.3 mM CaCl_2_, 1 mM MgSO_4_, 1 mM Na pyruvate, 1 mM NaHCO_3_, 10 mM HEPES, pH 7.4) with 0.1% BSA and 10 mM d-Glucose. Growth medium was removed 24 h after seeding, and cells were washed with assay buffer prior to the addition of 20 μL of assay buffer per well. Where appropriate, 10 μL vehicle or 10 μM (*R*)-navarixin (**2**) (in assay buffer) was added to wells, defining total and NSB, and cells were incubated for 30 min at 37 °C to ensure sufficient binding of the NSB ligand. Chosen fluorescent ligands were diluted in assay buffer to required concentrations (78 nM–10 μM) and 10 μL was added to the assay plate after 30 min incubation. The plate was then incubated at 37 °C for 1 h before addition of 1/240 furimazine solution (10 μL per well). The luciferase substrate was allowed to equilibrate for 5 min before measurement of NanoBRET signal using BMG PHERAstar (as per previously described membrane binding assays) with measurements taken every hour over a 3 h period. Fluorescent ligand affinity was derived as previously described for membrane binding experiments. All binding and functional data were analyzed using PRISM 9.0 (GraphPad Software, San Diego).

### Imaging of Fluorescent Ligand Binding in the HEK 293 SNAP-CXCR2-tsNanoLuc Cell Line

HEK 293 cells expressing SNAP-CXCR2-tsNanoLuc were seeded on black, clear bottom, poly-d-lysine-coated 96-well plates (Greiner 655097) at a density of 30,000 cells/well and allowed to grow overnight. Post-incubation, growth media were replaced with Hepes balances salt solution supplemented with 0.1% BSA (HBSS +0.1% BSA), 0.1 μM SNAP-surface AF488, 2 μg/mL Hoechst 33342 nuclear stain (H33342), and either HBSS +0.1% BSA or 10 μM (*R*)-navarixin (**2**). The assay plate was incubated for 30 min at 37 °C prior to addition of 1 μM compound **11a** to each well and allowed to equilibrate for 10 min before imaging. A Zeiss Celldiscoverer 7 microscope was employed to image cells, imaging one site per well using a 20×/0.95 objective and corresponding laser excitatory wavelengths (SNAP-surface AF488: 493 nm, BODIPY-630/650: 646 nm, H33342: 348 nm).

### Modeling

Docking was carried out using tools from Schrödinger software suite release 2023-1. The structures of the ligands were imported in Maestro in a MOL file format generated from ChemDraw (PerkinElmer Informatics release 19.1) and prepared with LigPrep retaining their specific chirality. The crystal structures were imported and prepared with Maestro’s protein preparation wizard, including water removal, H-bonding optimization using PROPKA at pH = 7, and energy minimization using OPLS3 force field. Grids were produced using Glide selecting Ala249^6.33^ or Glu249^6.33^ and Lys320^8.49^ as the centroid of the grid and Lys320^8.49^ hydrogen bonding as a constraint. The docking was performed both without constraints and with the selected constraint. For each ligand, 100 poses were minimized post docking and a maximum of 20 poses per output. Default settings were used unless otherwise stated. For all the ligands, the highest docking scoring pose was selected. Images were generated using PyMol (The PyMOL Molecular Graphics System, Version 2.0 Schrödinger, LLC). Molecular dynamics simulations were performed using AMBER 20.^75^ Molecular models for **6a** and **6b** were built and parameterized using antechamber (gaff2 forcefield) and immersed in a c 43 Å^3^ box of DMSO.^76^ After energy minimization, molecular dynamics simulations were run for a total of 10.3 ns in the NPT ensemble (Langevin dynamics with a collision frequency of 5 ps-1, temperature regulated to 300 K, pressure regulated with a Berendsen barostat, relaxation time 2 ps, SHAKE applied to all bonds, long-range electrostatic interactions evaluated using the PME method with a real-space cutoff of 8 Å). Discarding the first 300 ps as equilibration, analysis of the torsion angle and H-bonding data confirmed that the simulations were well converged. Simulation analysis was performed in Jupyter notebooks using tools from the MDTraj Python packagem.^77^

## Associated Content

### General Procedure 1: Conversion of Mixed Squarates to Chiral Squaramides **7a**,**b**, **10a**–**f**

To a solution of the required squaric acid monoamide monoesters compound (**6a**,**b**, **9a**–**f**) in 1.5 mL EtOH were added (*R*)-1-5-methylfuran-2-yl-propan-1-amine hydrochloride (1.1 equiv) and Et_3_N (1.1 equiv). The mixture was stirred at rt for 144 h, concentrated under reduced pressure, and purified by PTLC (Si).

### General Procedure 2: Amide Coupling for **9c**–**f**

To a solution of the Fmoc-protected amino acid (1 equiv) in anhydrous CH_2_Cl_2_ at 0 °C were added EDCI (1.2 equiv) and HOBt (1.1 equiv). The solution was stirred for 30 min prior to the addition of the required amine (**8a**,**8b**) (1.1 equiv) and DIPEA (2.1 equiv). The mixture was stirred at rt for 72 h, evaporated to dryness, and purified by PTLC (Si).

### General Procedure 3: Fluorophore Ligation via Amide Bond Formation for **11a**, **11c**–**f**

The desired Fmoc-protected amine congener (**10a**, **10c**–**f**) (1 equiv) was dissolved in DMF and treated with 20% piperidine in DMF. The solution was stirred at rt for 3 h and concentrated under reduced pressure to generate the desired amine congener. The compound was then dissolved in DMF (1 mL) and treated with BODIPY 630/650-X NHS ester (0.9 equiv). The solution was stirred at rt for 18 h in the dark and concentrated under reduced pressure. The reaction mixture was purified using PTLC (Si, MeOH/CH_2_Cl_2_, 5:95).

### *tert*-Butyl-(2-(2-hydroxy-3-nitrobenzamido)ethyl)carbamate (**4a**)

To a solution of 3-nitrosalicylic acid (**3**) (2.0 g, 10.9 mmol) in anhydrous CH_2_Cl_2_ (25 mL) at 0 °C were added oxalyl chloride (2.8 mL, 32.7 mmol) and DMF (2 drops) under a N_2_ atmosphere. The mixture was stirred for 1.5 h at rt and then concentrated under reduced pressure to give a yellow solid. To the solid were added anhydrous CH_2_Cl_2_ (25 mL) under a N_2_ atmosphere and *tert*-butyl (2-aminoethyl)carbamate (5.1 mL, 32.7 mmol) dropwise. The mixture was stirred at rt for 24 h and concentrated under reduced pressure to give a yellow solid. The solid was dissolved in CH_2_Cl_2_, and the organic layer was washed with KHSO_4_ and H_2_O and concentrated under reduced pressure. Purification by flash column chromatography (EtOAc/CH_2_Cl_2_, 50:50) gave 2.1 g (58%) of the title compound as a yellow solid. Mp 103–105 °C; ^1^H NMR (400 MHz, CDCl_3_) δ 12.95 (s, 1H), 8.33–8.11 (m, 3H), 7.05 (t, *J* = 8.1 Hz, 1H), 5.01 (s, 1H), 3.58 (q, *J* = 5.3 Hz, 2H), 3.42 (s, 2H), 1.43 (s, 9H); ^13^C NMR (101 MHz, CDCl_3_) δ 166.1, 157.3, 154.5, 136.4, 136.1, 129.1, 120.6, 118.9, 80.1, 41.7, 39.9, 28.3; LC/MS *m*/*z* calculated for C_14_H_20_N_3_O_6_ [M + H]^+^: 326.1, found 326.2, *t*_R_ = 3.02 min.

### *tert*-Butyl (2-(2-hydroxy-*N*-methyl-3-nitrobenzamido)ethyl)carbamate (**4b**)

To a solution of 3-nitrosalicylic acid (**3**) (1.5 g, 8.2 mmol) in anhydrous CH_2_Cl_2_ (20 mL) at 0 °C were added oxalyl chloride (2.1 mL, 24.6 mmol) and DMF (2 drops) under a N_2_ atmosphere. The mixture was stirred for 1.5 h at rt and then concentrated under reduced pressure to give a yellow solid. To the solid were added anhydrous CH_2_Cl_2_ (20 mL) under a N_2_ atmosphere and *tert*-butyl-(2-(methylamino)ethyl)carbamate (4.2 mL, 24.6 mmol) dropwise. The mixture was stirred at rt for 24 h and concentrated under reduced pressure to give a yellow solid. The solid was dissolved in CH_2_Cl_2_, and the organic layer was washed with KHSO_4_ and H_2_O and concentrated under reduced pressure Purification by flash column chromatography (EtOAc/CH_2_Cl_2_, 50:50) gave 1.9 g (70%) of the title compound as a yellow oil. ^1^H NMR (400 MHz, CDCl_3_) δ 10.85 (s, 1H), 8.14 (d, *J* = 8.5 Hz, 1H), 7.66–7.50 (m, 1H), 7.05 (t, *J* = 7.9 Hz, 1H), 5.03 (s, 1H), 3.79–2.82 (m, 7H), 1.49–1.31 (m, 9H); ^13^C NMR (101 MHz, CDCl_3_) δ 167.2, 156.2, 150.9, 136.0, 133.8, 128.5, 126.0, 120.4, 79.4, 47.1, 38.1, 36.8, 28.4; LC/MS *m*/*z* calculated for C_15_H_22_N_3_O_6_ [M + H]^+^: 340.1, found 340.1, *t*_R_ = 2.79 min.

### *tert*-Butyl-(2-(3-amino-2-hydroxybenzamido)ethyl)carbamate (**5a**)

To *tert*-butyl (2-(2-hydroxy-3-nitrobenzamido)ethyl)carbamate (**4a**) (2.0 g, 6.1 mmol) in EtOH (50 mL) at rt was added 10% Pd/C (200 mg). The mixture was stirred under a H_2_ atmosphere at rt for 2.5 h. The mixture was filtered through a pad of Celite and concentrated under reduced pressure to give 1.8 g (99%) of a brown viscous solid. ^1^H NMR (400 MHz, CDCl_3_) δ 12.66 (s, 1H), 7.65 (s, 1H), 6.87 (dd, *J* = 8.0, 1.4 Hz, 1H), 6.79 (dd, *J* = 7.7, 1.4 Hz, 1H), 6.64 (t, *J* = 7.9 Hz, 1H), 5.12 (d, *J* = 7.2 Hz, 1H), 3.57–3.26 (m, 6H), 1.42 (s, 9H); ^13^C NMR (101 MHz, CDCl_3_) δ 171.0, 157.8, 149.7, 136.4, 118.5, 118.0, 114.9, 113.3, 80.2, 42.0, 39.5, 28.3; LC/MS *m*/*z* calculated for C_14_H_22_N_3_O_4_ [M + H]^+^: 296.1, found 296.3 , *t*_R_ = 2.68 min.

### *tert*-Butyl-(2-(3-amino-2-hydroxy-*N*-methylbenzamido)ethyl) Carbamate (**5b**)

To *tert*-butyl-(2-(2-hydroxy-*N*-methyl-3-nitrobenzamido)ethyl)carbamate (**4b**) (1.8 g, 5.3 mmol) in EtOH (30 mL) at rt was added 10% Pd/C (180 mg). The mixture was stirred under a H_2_ atmosphere at rt for 3.5 h. The mixture was filtered through a pad of Celite and concentrated under reduced pressure to give 1.6 g (81%) of a brown viscous solid. ^1^H NMR (400 MHz, CDCl_3_) δ 6.80–6.63 (m, 3H), 5.00 (s, 1H), 3.64 (t, *J* = 6.1 Hz, 2H), 3.40 (q, *J* = 6.2 Hz, 2H), 3.18 (s, 3H), 1.41 (s, 9H) (OH and aniline NH_2_ protons not visible); ^13^C NMR (101 MHz, CDCl_3_) δ 172.5, 156.2, 146.2, 136.0, 118.7, 118.0, 117.5, 79.6, 58.4, 50.7, 38.1, 28.3; LC/MS *m*/*z* calculated for C_15_H_24_N_3_O_4_ [M + H]^+^: 310.1, found 310.2 , *t*_R_ = 2.37 min.

### *tert-*Butyl-(2-(3-((2-ethoxy-3,4-dioxocyclobut-1-en-1-yl)amino)-2-hydroxybenzamido)ethyl) Carbamate (**6a**)

To a solution of *tert*-butyl (2-(3-amino-2-hydroxybenzamido)ethyl)carbamate (**5a**) (1.7 g, 5.7 mmol) in EtOH (30 mL) was added diethoxysquarate (0.89 mL, 6.1 mmol) dropwise. The mixture was stirred at rt for 24 h and concentrated under reduced pressure. Purification by flash column chromatography (EtOAc/CH_2_Cl_2_, 30:70) gave 1.7 g (71%) of the title compound as a pink solid. Mp 88–90 °C; ^1^H NMR (500 MHz, DMSO-*d*_6_) δ 13.55 (s, 1H), 10.24 (s, 1H), 8.99 (t, *J* = 5.7 Hz, 1H), 7.70 (dd, *J* = 8.2, 1.5 Hz, 1H), 7.38 (dd, *J* = 7.8, 1.4 Hz, 1H), 6.95 (t, *J* = 6.0 Hz, 1H), 6.88 (t, *J* = 8.0 Hz, 1H), 4.67 (q, *J* = 7.0 Hz, 2H), 3.37–3.29 (m, 2H + H_2_O), 3.13 (q, *J* = 6.2 Hz, 2H), 1.26–1.40 (m, 12H); ^13^C NMR (101 MHz, DMSO-*d*_6_) δ 188.0, 184.1, 178.1, 170.9, 169.8, 155.7, 154.4, 128.1, 125.9, 124.3, 117.4, 114.6, 77.7, 69.1, 39.1, 38.8, 28.2, 15.5; LC/MS *m*/*z* calculated for C_20_H_26_N_3_O_7_ [M + H]^+^: 420.1, found 420.1 , *t*_R_ = 2.90 min.

### *tert*-Butyl-(2-(3-((2-ethoxy-3,4-dioxocyclobut-1-en-1-yl)amino)-2-hydroxy-*N*-methylbenzamido)ethyl) Carbamate (**6b**)

To a solution of *tert*-butyl (2-(3-amino-2-hydroxy-*N*-methylbenzamido)ethyl)carbamate (**5b**) (1.3 g, 4.2 mmol) in EtOH (15 mL) was added diethoxysquarate (0.65 mL, 4.4 mmol) dropwise. The mixture was stirred at rt for 40 h and concentrated under reduced pressure. Purification by flash column chromatography (MeOH/CH_2_Cl_2_, 4:96) gave 0.7 g (39%) of the title compound as a pink solid. Mp 75–77 °C; ^1^H NMR (500 MHz, DMSO-*d*_6_) δ 10.11 (s, 1H), 9.49 (s, 1H), 7.19 (dd, *J* = 7.8, 1.7 Hz, 1H), 7.02 (s, 1H), 6.87 (t, *J* = 7.7 Hz, 2H), 4.65 (q, *J* = 7.1 Hz, 2H), 3.43 (t, *J* = 7.0 Hz, 2H + H_2_O), 3.17 (s, 2H), 2.89 (s, 3H), 1.30–1.42 (m, 12H); ^13^C NMR (126 MHz, DMSO-*d*_6_) δ 188.2, 184.1, 178.0, 171.2, 168.2, 155.6, 146.7, 126.0, 125.9, 125.6, 125.2, 119.3, 77.7, 69.1, 46.8, 37.5, 36.8, 28.2, 15.6; LC/MS *m*/*z* calculated for C_21_H_28_N_3_O_7_ [M + H]^+^: 434.1, found 434.2, *t*_R_ = 2.56 min.

### *tert*-Butyl-(*R*)-(2-(2-hydroxy-3-((2-((1-(5-methylfuran-2-yl)propyl)amino)-3,4-dioxocyclobut-1-en-1-yl)amino)benzamido)ethyl)carbamate (**7a**)

The title compound was synthesized following general procedure 1 using *tert*-butyl-(2-(3-((2-ethoxy-3,4-dioxocyclobut-1-en-1-yl)amino)-2-hydroxybenzamido)ethyl)carbamate (**6a**) (600 mg, 1.43 mmol). Purification by flash column chromatography (MeOH/CH_2_Cl_2_, 3:97) gave the title compound (410 mg, 56%) as a pink solid. Mp 128–130 °C; ^1^H NMR (400 MHz, DMSO-*d*_6_) δ 13.96 (s, 1H), 9.34 (s, 1H), 9.00 (t, *J* = 5.7 Hz, 1H), 8.70 (d, *J* = 9.0 Hz, 1H), 7.99 (d, *J* = 8.0 Hz, 1H), 7.52 (d, *J* = 8.1 Hz, 1H), 7.02–6.80 (m, 2H), 6.26 (d, *J* = 3.1 Hz, 1H), 6.05 (dd, *J* = 3.1, 1.2 Hz, 1H), 5.13 (q, *J* = 7.8 Hz, 1H), 3.33 (q, *J* = 6.5 Hz, 2H), 3.13 (q, *J* = 6.2 Hz, 2H), 2.26 (d, *J* = 1.0 Hz, 3H), 2.03–1.77 (m, 2H), 1.36 (s, 9H), 0.92 (t, *J* = 7.3 Hz, 3H); ^13^C NMR (101 MHz, DMSO-*d*_6_) δ 184.5, 180.6, 170.6, 169.0, 163.6, 156.2, 152.5, 151.8, 151.4, 128.4, 123.5, 121.2, 118.4, 114.3, 108.0, 106.9, 78.2, 55.3, 53.3, 28.6, 27.7, 13.8, 10.7; LC/MS *m*/*z* calculated for C_26_H_33_N_4_O_7_ [M + H]^+^: 513.2, found 513.2 , *t*_R_ = 6.36 min; HRMS (TOF ES^+^) calculated for C_26_H_33_N_4_O_7_ [M + H]^+^: 513.2344, found 513.2315; [α]^24.5 °C^_D_ = +72 (0.5; MeOH).

### *tert-*Butyl-(*R*)-(2-(2-hydroxy-*N*-methyl-3-((2-((1-(5-methylfuran-2-yl)propyl)amino)-3,4-dioxocyclobut-1-en-1-yl)amino)benzamido)ethyl)carbamate (**7b**)

The title compound was synthesized following general procedure 1 using *tert*-butyl-(2-(3-((2-ethoxy-3,4-dioxocyclobut-1-en-1-yl)amino)-2-hydroxy-*N*-methylbenzamido)ethyl) carbamate (**6b**) (10 mg, 0.02 mmol). Purification by flash column chromatography (MeOH/CH_2_Cl_2_, 3:97) gave the title compound (5 mg, 50%) as a pink oil. ^1^H NMR (400 MHz, DMSO-*d*_6_) δ 10.07–8.75 (m, 2H), 8.49 (s, 1H), 7.79 (dd, *J* = 7.6, 1.9 Hz, 1H), 6.97–6.79 (m, 2H), 6.62 (s, 1H), 6.24 (d, *J* = 3.1 Hz, 1H), 6.04 (d, *J* = 3.1 Hz, 1H), 5.17 (s, 1H), 3.42 (t, *J* = 6.4 Hz, 2H), 3.17 (t, *J* = 6.2 Hz, 2H), 2.98 (s, 3H), 2.28 (s, 3H), 2.05–1.83 (m, 2H), 1.38 (s, 9H), 0.96 (t, *J* = 7.3 Hz, 3H); ^13^C NMR (101 MHz, DMSO-*d*_6_) δ184.3, 180.6, 169.0, 168.4, 163.9, 156.1, 152.5, 151.8, 132.9, 129.2, 122.7, 116.3, 108.0, 106.9, 78.2, 75.6, 53.2, 28.7, 27.6, 13.8, 10.7; LC/MS *m*/*z* calculated for C_27_H_35_N_4_O_7_ [M + H]^+^: 527.2, found: 527.2, *t*_R_ = 2.74 min; HRMS (TOF ES^+^) calculated for C_27_H_35_N_4_O_7_ [M + H]^+^: 527.2500, found 527.2485; [α]^24.5 °C^_D_ = +200 (0.1; MeOH).

### *N*-(2-Aminoethyl)-3-((2-ethoxy-3,4-dioxocyclobut-1-en-1-yl)amino)-2-hydroxybenzamide (**8a**)

*tert*-Butyl-(2-(3-((2-ethoxy-3,4-dioxocyclobut-1-en-1-yl)amino)-2-hydroxybenzamido)ethyl) carbamate (**6a**) (100 mg, 0.2 mmol) was dissolved in 1,4-dioxane (2 mL) and it was treated with a solution of HCl in 1,4-dioxane (4 M, 2 mL). The solution was stirred at rt for 18 h and concentrated under reduced pressure to afford 70 mg (100%) of the hydrochloride salt of the title compound as a white gum that was used without further purification. ^1^H NMR (400 MHz, DMSO-*d*_6_) δ 13.28 (s, 1H), 10.28 (s, 1H), 9.25 (t, *J* = 5.6 Hz, 1H), 7.99 (s, 3H), 7.81 (dd, *J* = 8.2, 1.5 Hz, 1H), 7.40 (dd, *J* = 7.9, 1.4 Hz, 1H), 6.91 (td, *J* = 8.0, 3.0 Hz, 1H), 4.67 (q, *J* = 7.0 Hz, 2H), 3.62−3.48 (m, 2H), 3.04 (dq, *J* = 11.9, 5.9 Hz, 2H), 1.34 (t, *J* = 7.1 Hz, 3H); ^13^C NMR (101 MHz, DMSO-*d*_6_) δ 184.6, 178.6, 171.4, 170.7, 154.8, 128.9, 126.4, 125.3, 118.0, 115.1, 69.6, 66.8, 38.7, 37.3, 16.0; LC/MS *m*/*z* calculated for C_15_H_18_N_3_O_5_ [M + H]^+^: 320.1, found 320.1, *t*_R_ = 2.13 min.

### *N*-(2-Aminoethyl)-3-((2-ethoxy-3,4-dioxocyclobut-1-en-1-yl)amino)-2-hydroxy-*N*-methylbenzamide (**8b**)

.To a solution of *tert*-butyl-(2-(3-((2-ethoxy-3,4-dioxocyclobut-1-en-1-yl)amino)-2-hydroxy-*N*-methylbenzamido)ethyl) carbamate (**6b**) (50 mg, 0.1 mmol) in CH_2_Cl_2_ (2 mL) was added TFA (0.6 mL, 0.008 mmol). The solution was stirred at rt for 18 h and concentrated under reduced pressure to afford 40 mg (100%) of the TFA salt of the title compound as a white gum that was used without further purification. ^1^H NMR (400 MHz, DMSO-*d*_6_) δ 9.82 (s, 1H), 7.80 (s, 3H), 7.28 (d, *J* = 7.8 Hz, 1H), 7.11 (d, *J* = 7.5 Hz, 1H), 6.92 (t, *J* = 7.7 Hz, 1H), 4.69 (q, *J* = 7.0 Hz, 2H), 3.63 (t, *J* = 6.6 Hz, 2H), 3.07 (t, *J* = 6.5 Hz, 2H), 2.91 (s, 3H), 1.38 (t, *J* = 7.0 Hz, 3H); ^13^C NMR (101 MHz, DMSO-*d*_6_) δ 184.5, 178.5, 169.6, 161.0, 158.8, 126.5, 118.2, 115.3, 69.6, 55.3, 37.1, 16.0. LC/MS *m*/*z* calculated for C_16_H_20_N_3_O_5_ [M + H]^+^: 334.1, found 334.2, *t*_R_ = 0.96 min.

### (9*H*-Fluoren-9-yl)methyl(2-(3-((2-ethoxy-3,4-dioxocyclobut-1-en-1-yl)amino)-2-hydroxybenzamido)ethyl)carbamate (**9a**)

To a solution of *N*-(2-aminoethyl)-3-((2-ethoxy-3,4-dioxocyclobut-1-en-1-yl)amino)-2-hydroxybenzamide (**8a**) (30 mg, 0.1 mmol) in DMF (1.5 mL) were added DIPEA (37 μL, 0.21 mmol) and Fmoc-OSU (37 mg, 0.11 mmol). The solution was stirred for 72 h at rt, and it was concentrated under reduced pressure. Purification (PTLC, MeOH/CH_2_Cl_2_, 1:99) gave the title compound (22 mg, 39%) as a yellow oil. ^1^H NMR (400 MHz, DMSO-*d*_6_) δ 13.53 (s, 1H), 10.24 (s, 1H), 9.06 (s, 1H), 7.88 (d, *J* = 7.5 Hz, 2H), 7.69 (dd, *J* = 11.5, 8.0 Hz, 3H), 7.50–7.26 (m, 6H), 6.87 (t, *J* = 7.9 Hz, 1H), 4.67 (q, *J* = 7.1 Hz, 2H), 4.38–4.18 (m, 3H), 3.37 (q, *J* = 6.4 Hz, 3H), 3.21 (t, *J* = 6.1 Hz, 2H), 1.34 (t, *J* = 7.1 Hz, 3H); ^13^C NMR (101 MHz, DMSO-*d*_6_) δ 184.5, 178.6, 171.2, 170.3, 162.7, 156.7, 144.3, 141.2, 128.0, 127.5, 126.5, 125.6, 124.7, 120.5, 115.1, 69.6, 55.3, 49.0, 36.2, 31.2, 16.0; LC/MS *m*/*z* calculated for C_30_H_28_N_3_O_7_ [M + H]^+^: 542.1, found 542.2, *t*_R_ = 3.03 min.

### (9*H*-Fluoren-9-yl)methyl (2-(3-((2-ethoxy-3,4-dioxocyclobut-1-en-1-yl)amino)-2-hydroxy-*N*-methylbenzamido)ethyl)carbamate (**9b**)

To a solution of *N*-(2-aminoethyl)-3-((2-ethoxy-3,4-dioxocyclobut-1-en-1-yl)amino)-2-hydroxy-*N*-methylbenzamide (**8b**) (25 mg, 0.075 mmol) in DMF (1.5 mL) were added DIPEA (28 μL, 0.16 mmol) and Fmoc-OSU (28 mg, 0.083 mmol). The solution was stirred for 96 h at rt, and it was concentrated under reduced pressure. Purification (PTLC, MeOH/CH_2_Cl_2_, 3:97) gave the title compound (24 mg, 58%) as a yellow oil. ^1^H NMR (400 MHz, DMSO-*d*_6_) δ 7.89 (d, *J* = 7.5 Hz, 2H), 7.69 (d, *J* = 7.5 Hz, 2H), 7.46–7.26 (m, 4H), 7.19 (s, 1H), 7.08–6.55 (m, 2H), 4.63 (q, *J* = 7.0 Hz, 2H), 4.41–4.08 (m, 3H), 3.57–2.74 (m, 7H + H_2_0), 1.31 (t, *J* = 7.1 Hz, 3H); ^13^C NMR (101 MHz, DMSO-*d*_6_) δ 188.8, 184.4, 178.5, 171.5, 168.7, 144.3, 141.2, 128.0, 127.5, 126.5, 125.9, 125.6, 125.2, 120.5, 119.4, 69.5, 65.8, 49.6, 47.1, 29.4, 16.0; LC/MS *m*/*z* calculated for C_31_H_30_N_3_O_7_ [M + H]^+^: 556.2, found 556.1, *t*_R_ = 2.82 min.

### (9*H*-Fluoren-9-yl)methyl(2-((2-(3-((2-ethoxy-3,4-dioxocyclobut-1-en-1-yl)amino)-2-hydroxybenzamido)ethyl)amino)-2-oxoethyl)carbamate (**9c**)

The title compound was synthesized following general procedure 2 using (((9*H*-fluoren-9-yl)methoxy)carbonyl)glycine (27 mg, 0.09 mmol) and *N*-(2-aminoethyl)-3-((2-ethoxy-3,4-dioxocyclobut-1-en-1-yl)amino)-2-hydroxybenzamide (**8a**) (30 mg, 0.1 mmol). Purification (PTLC, MeOH/CH_2_Cl_2_, 3:97 + 1% acetic acid) gave the title compound (25 mg, 42%) as a yellow oil. ^1^H NMR (400 MHz, DMSO-*d*_6_) δ 13.49 (s, 1H), 10.23 (s, 1H), 9.07 (s, 1H), 8.04 (t, *J* = 5.7 Hz, 1H), 7.89 (d, *J* = 7.5 Hz, 2H), 7.76–7.64 (m, 3H), 7.52 (q, *J* = 7.1 Hz, 1H), 7.46–7.26 (m, 5H), 6.85 (t, *J* = 7.9 Hz, 1H), 4.67 (q, *J* = 7.1 Hz, 2H), 4.35–4.01 (m, 4H), 3.59 (d, *J* = 6.0 Hz, 2H), 3.17 (s, 2H), 1.34 (t, *J* = 7.0 Hz, 3H) (1H, under H_2_O peak); ^13^C NMR (101 MHz, DMSO-*d*_6_) δ 184.5, 178.6, 172.4, 170.2, 169.9, 156.9, 144.3, 141.2, 128.1, 127.5, 126.5, 125.7, 124.7, 120.5, 115.1, 69.6, 66.1, 55.3, 47.1, 44.0, 21.5, 16.0; LC/MS *m*/*z* calculated for C_32_H_31_N_4_O_8_ [M + H]^+^: 599.2, found 599.1 , *t*_R_ = 2.81 min.

### (9*H*-Fluoren-9-yl)methyl(2-((2-(3-((2-ethoxy-3,4-dioxocyclobut-1-en-1-yl)amino)-2-hydroxy-*N*-methylbenzamido)ethyl)amino)-2-oxoethyl)carbamate (**9d**)

The title compound was synthesized following general procedure 2 using (((9*H*-fluoren-9-yl)methoxy)carbonyl)glycine (33 mg, 0.11 mmol) and *N*-(2-aminoethyl)-3-((2-ethoxy-3,4-dioxocyclobut-1-en-1-yl)amino)-2-hydroxybenzamide (**8b**) (40 mg, 0.1 mmol). Purification by flash column chromatography ((3:1, EtOAc:IMS)/cyclohexane 60:40 + 0.1% acetic acid) gave the title compound (8 mg, 11%) as a yellow oil. ^1^H NMR (400 MHz, CD_3_OD) δ 7.78 (d, *J* = 7.6 Hz, 2H), 7.64 (d, *J* = 7.5 Hz, 2H), 7.44–7.18 (m, 6H), 7.10 (d, *J* = 7.7 Hz, 1H), 6.90 (t, *J* = 7.8 Hz, 1H), 4.70 (q, *J* = 7.1 Hz, 2H), 4.34 (t, *J* = 7.6 Hz, 2H), 4.19 (t, *J* = 6.9 Hz, 1H), 3.74 (m, 6H), 3.05 (s, 3H), 2.01 (s, 1H), 1.39 (t, *J* = 7.1 Hz, 3H) (OH and NH not visible); ^13^C NMR (101 MHz, MeOD) δ 184.8, 171.1, 170.5, 157.6, 146.3, 143.8, 141.1, 127.4, 126.7, 126.3, 125.1, 124.8, 124.6, 119.6, 119.5, 69.7, 66.7, 43.6, 36.8, 19.4, 14.6; LC/MS *m*/*z* calculated for C_33_H_33_N_4_O_8_[M + H]^+^: 613.2 , found 613.1, *t*_R_ = 2.73 min.

### (9*H*-Fluoren-9-yl)methyl(3-((2-(3-((2-ethoxy-3,4-dioxocyclobut-1-en-1-yl)amino)-2-hydroxybenzamido)ethyl)amino)-3-oxopropyl)carbamate (**9e**)

The title compound was synthesized following general procedure 2 using 3-((((9*H*-fluoren-9-yl)methoxy)carbonyl)amino)propanoic acid (28 mg, 0.09 mmol) and *N*-(2-aminoethyl)-3-((2-ethoxy-3,4-dioxocyclobut-1-en-1-yl)amino)-2-hydroxybenzamide (**8a**) (30 mg, 0.1 mmol). Purification by flash column chromatography (MeOH/CH_2_Cl_2_, 3:97 + 1% acetic acid) gave the title compound (25 mg, 40%) as a yellow oil. ^1^H NMR (400 MHz, DMSO-*d*_6_) δ 12.31 (s, 2H), 9.54 (s, 1H), 8.07 (t, *J* = 5.6 Hz, 1H), 7.88 (d, *J* = 7.5 Hz, 2H), 7.66 (t, *J* = 8.0 Hz, 3H), 7.49–7.18 (m, 6H), 6.72 (t, *J* = 7.9 Hz, 1H), 4.68 (q, *J* = 7.0 Hz, 2H), 4.27 (d, *J* = 6.6 Hz, 2H), 4.19 (t, *J* = 7.0 Hz, 1H), 3.40–3.16 (m, 6H), 2.27 (t, *J* = 7.2 Hz, 2H), 1.34 (t, *J* = 7.0 Hz, 3H); ^13^C NMR (101 MHz, DMSO-*d*_6_) δ 188.6, 184.2, 178.4, 172.5, 171.0, 170.6, 169.9, 156.5, 144.3, 141.1, 128.0, 127.5, 127.1, 125.6, 124.9, 120.5, 115.7, 69.6, 65.8, 47.1, 38.5, 37.5, 36.2, 29.4, 21.5, 16.0; LC/MS *m*/*z* calculated for C_33_H_33_N_4_O_8_ [M + H]^+^: 613.2, found 613.2, *t*_R_ = 1.91 min.

### (9*H*-Fluoren-9-yl)methyl(3-((2-(3-((2-ethoxy-3,4-dioxocyclobut-1-en-1-yl)amino)-2-hydroxy-*N*-methylbenzamido)ethyl)amino)-3-oxopropyl)carbamate (**9f**)

The title compound was synthesized following general procedure 2 using 3-((((9*H*-fluoren-9-yl)methoxy)carbonyl)amino)propanoic acid (34 mg, 0.11 mmol) and *N*-(2-aminoethyl)-3-((2-ethoxy-3,4-dioxocyclobut-1-en-1-yl)amino)-2-hydroxybenzamide (**8b**) (40 mg, 0.1 mmol). Purification by flash column chromatography ((3:1, EtOAc:IMS)/cyclohexane 60:40 + 0.1% acetic acid) gave the title compound (18 mg, 24%) as a yellow oil. ^1^H NMR (400 MHz, CD_3_OD) δ 7.76 (t, *J* = 5.9 Hz, 2H), 7.63–7.55 (m, 2H), 7.44–7.19 (m, 6H), 7.08 (d, *J* = 7.6 Hz, 1H), 6.91 (t, *J* = 7.8 Hz, 1H), 4.69 (q, *J* = 7.1 Hz, 2H), 4.27 (d, *J* = 6.8 Hz, 2H), 4.13 (d, *J* = 7.5 Hz, 1H), 3.71–3.34 (m, 6H), 3.03 (d, *J* = 5.8 Hz, 3H), 2.37 (s, 2H), 2.01 (s, 1H),1.38 (t, *J* = 7.1 Hz, 3H) (OH not visible); ^13^C NMR (101 MHz, MeOD) δ 184.8, 173.8, 170.4, 157.3, 146.3, 143.9, 143.8, 141.1, 127.3, 126.7, 126.3, 125.0, 124.7, 124.5, 124.1, 119.6, 119.5, 119.5, 69.9, 69.7, 66.4, 36.9, 36.6, 35.8, 19.3, 14.7; LC/MS *m*/*z* calculated for C_3__4_H_35_N_4_O_8_ [M + H]^+^: 627.2, found 627.2, *t*_R_ = 2.75 min.

### (*R*)-(Fluoren-9-yl)methyl(*R*)-(2-(2-hydroxy-3-((2-((1-(5-methylfuran-2-yl)propyl)amino)-3,4-dioxocyclobut-1-en-1-yl)amino)benzamido)ethyl)carbamate (**10a**)

The title compound was synthesized following general procedure 1 using 9*H*-fluoren-9-yl)methyl(2-(3-((2-ethoxy-3,4-dioxocyclobut-1-en-1-yl)amino)-2-hydroxybenzamido)ethyl) carbamate (**9a**) (20 mg, 0.04 mmol). Purification by flash column chromatography (MeOH/CH_2_Cl_2_, 3:97) gave the title compound (8 mg, 35%) as a yellow oil. ^1^H NMR (400 MHz, DMSO-*d*_6_) δ 13.93 (s, 1H), 9.34 (s, 1H), 9.05 (s, 1H), 8.70 (d, *J* = 9.0 Hz, 1H), 7.99 (d, *J* = 8.0 Hz, 1H), 7.88 (d, *J* = 7.5 Hz, 2H), 7.67 (d, *J* = 7.5 Hz, 2H), 7.52 (d, *J* = 8.1 Hz, 1H), 7.46 (t, *J* = 5.9 Hz, 1H), 7.44–7.36 (m, 2H), 7.31 (td, *J* = 7.5, 1.1 Hz, 2H), 6.87 (s, 1H), 6.26 (d, *J* = 3.1 Hz, 1H), 6.05 (dd, *J* = 3.2, 1.2 Hz, 1H), 5.13 (q, *J* = 7.7 Hz, 1H), 4.30 (d, *J* = 6.9 Hz, 2H), 4.20 (t, *J* = 6.7 Hz, 1H), 3.37 (q, *J* = 6.2 Hz, 2H), 3.21 (q, *J* = 6.1 Hz, 2H), 2.26 (d, *J* = 1.0 Hz, 3H), 2.01–1.80 (m, 2H), 0.92 (t, *J* = 7.3 Hz, 3H); ^13^C NMR (101 MHz, DMSO-*d*_6_) δ 184.4, 180.6, 170.6, 169.0, 163.6, 156.7, 152.5, 151.8, 144.3, 141.2, 128.0, 127.5, 125.6, 123.5, 121.2, 120.5, 114.4, 108.0, 106.9, 65.8, 53.3, 47.1, 27.6, 13.8, 10.7; LC/MS *m*/*z* calculated for C_36_H_34_N_4_NaO_7_ [M + Na]^+^: 657.2, found 657.2 , *t*_R_ = 1.84 min; HRMS (TOF ES^+^) calculated for C_36_H_34_N_4_NaO_7_ [M + Na]^+^: 657.2320 , found 657.2286; [α]^24.5 °C^_D_ = + 64(0.3; MeOH).

### (9*H*-Fluoren-9-yl)methyl(*R*)-(2-(2-hydroxy-*N*-methyl-3-((2-((1-(5-methylfuran-2-yl)propyl)amino)-3,4-dioxocyclobut-1-en-1-yl)amino)benzamido)ethyl)carbamate (**10b**)

The title compound was synthesized following general procedure 1 using (9*H*-Fluoren-9-yl)methyl (2-(3-((2-ethoxy-3,4-dioxocyclobut-1-en-1-yl)amino)-2-hydroxy-*N*-methylbenzamido)ethyl)carbamate (**9b**) (23 mg, 0,04 mmol). Purification (PTLC, MeOH/CH_2_Cl_2_, 10:90) gave the title compound (12 mg, 48%) as a yellow oil. ^1^H NMR (400 MHz, DMSO-*d*_6_) δ 9.75 (s, 1H), 9.28 (s, 1H), 8.65 (d, *J* = 9.0 Hz, 1H), 7.88 (d, *J* = 7.5 Hz, 2H), 7.77 (d, *J* = 7.0 Hz, 1H), 7.67 (d, *J* = 7.5 Hz, 2H), 7.45–7.25 (m, 4H), 6.82 (s, 2H),6.24 (d, *J* = 3.1 Hz, 1H), 6.04 (d, *J* = 3.1 Hz, 1H), 5.13 (q, *J* = 7.7 Hz, 1H), 4.42–4.12 (m, 3H), 3.33 (s, 4H + H_2_O), 2.89 (s, 3H), 2.25 (s, 3H), 2.04–1.76 (m, 2H), 0.91 (t, *J* = 7.3 Hz, 3H) (OH not visible);^13^C NMR (101 MHz, DMSO-*d*_6_) δ 183.5, 181.1, 170.7, 168.7, 163.7, 152.9, 151.6, 143.0, 139.8, 137.9, 130.6, 129.4, 127.7, 124.6, 123.4, 121.8, 120.5, 115.7, 110.2, 107.7, 106.8, 53.1, 50.2, 36.7, 34.0, 29.5, 27.5, 13.8, 10.7; LC/MS *m*/*z* calculated for C_37_H_36_N_4_NaO_7_ [M + Na]^+^: 671.2, found 671.2, *t*_R_ = 3.06 min; [α]^24.5 °C^_D_ = + 266 (0.3; MeOH).

### (9*H*-Fluoren-9-yl)methyl(*R*)-(2-((2-(2-hydroxy-3-((2-((1-(5-methylfuran-2-yl)propyl)amino)-3,4-dioxocyclobut-1-en-1-yl)amino)benzamido)ethyl)amino)-2-oxoethyl)carbamate (**10c**)

The title compound was synthesized following general procedure 1 using (9*H*-fluoren-9-yl)methyl (2-((2-(3-((2-ethoxy-3,4-dioxocyclobut-1-en-1-yl)amino)-2-hydroxybenzamido)ethyl)amino)-2-oxoethyl)carbamate (**9c**) (24 mg, 0.04 mmol). Purification (PTLC, MeOH/CH_2_Cl_2_, 4:96) gave the title compound (11 mg, 40%) as a yellow oil. ^1^H NMR (400 MHz, DMSO-*d*_6_) δ 13.88 (s, 1H), 9.35 (s, 1H), 9.04 (s, 1H), 8.70 (d, *J* = 9.0 Hz, 1H), 8.04 (t, *J* = 5.7 Hz, 1H), 7.98 (d, *J* = 8.0 Hz, 1H), 7.89 (d, *J* = 7.5 Hz, 2H), 7.70 (d, *J* = 7.5 Hz, 2H), 7.59–7.46 (m, 2H), 7.41 (t, *J* = 7.4 Hz, 2H), 7.32 (td, *J* = 7.4, 1.2 Hz, 2H), 6.84 (s, 1H), 6.26 (d, *J* = 3.1 Hz, 1H), 6.05 (dd, *J* = 3.1, 1.2 Hz, 1H), 5.13 (q, *J* = 7.8 Hz, 1H), 4.29–4.14 (m, 3H), 3.60 (d, *J* = 6.0 Hz, 2H), 3.36 (t, *J* = 5.9 Hz, 3H), 3.29 (d, *J* = 6.4 Hz, 2H), 2.26 (d, *J* = 1.0 Hz, 3H), 2.01–1.79 (m, 2H), 0.92 (t, *J* = 7.3 Hz, 3H); ^13^C NMR (101 MHz, DMSO-*d*_6_) δ 180.6, 170.5, 169.9, 169.0, 163.6, 156.9, 152.5, 151.8, 144.3, 141.1, 128.1, 127.5, 125.7, 120.5, 108.0, 106.9, 66.1, 53.3, 47.0, 44.0, 38.4, 27.6, 13.8, 10.7; LC/MS *m*/*z* calculated for C_38_H_37_N_5_NaO_8_ [M + Na]^+^: 714.2, found 714.2 , *t*_R_ = 1.94 min; HRMS (TOF ES^+^) calculated for C_38_H_37_N_5_NaO_8_ [M + Na]^+^: 714.2534, found 714.2519; [α]^24.5 °C^_D_ = +100 (0.2; MeOH).

### (9*H*-Fluoren-9-yl)methyl(*R*)-(2-((2-(2-hydroxy-*N*-methyl-3-((2-((1-(5-methylfuran-2-yl)propyl)amino)-3,4-dioxocyclobut-1-en-1-yl)amino)benzamido)ethyl)amino)-2-oxoethyl)carbamate (**10d**)

The title compound was synthesized following general procedure 1 using (9*H*-fluoren-9-yl)methyl(2-((2-(3-((2-ethoxy-3,4-dioxocyclobut-1-en-1-yl)amino)-2-hydroxy-*N*-methylbenzamido)ethyl)amino)-2-oxoethyl)carbamate (**9d**) (7 mg, 0.01 mmol). Purification by flash column chromatography (MeOH/CH_2_Cl_2_, 3:97) gave the title compound (7 mg, 90%) as a yellow oil (purity 95%). ^1^H NMR (400 MHz, DMSO-*d*_6_)δ 9.74 (s, 1H), 9.29 (s, 1H), 8.67 (d, *J* = 9.0 Hz, 1H), 7.89 (d, *J* = 7.5 Hz, 2H), 7.78 (d, *J* = 7.9 Hz, 1H), 7.70 (d, *J* = 7.5 Hz, 2H), 7.61–7.47 (m, 1H), 7.41 (t, *J* = 7.4 Hz, 2H), 7.36–7.25 (m, 2H), 6.84 (s, 2H), 6.24 (d, *J* = 3.1 Hz, 1H), 6.03 (d, *J* = 3.3 Hz, 1H), 5.13 (q, *J* = 7.7 Hz, 1H), 4.27 (d, *J* = 7.0 Hz, 2H), 4.24–4.15 (m, 1H), 3.56 (s, 2H), 2.92 (s, 2H), 2.25 (s, 3H), 1.99–1.77 (m, 2H), 1.23 (s, 3H), 0.90 (q, *J* = 8.2 Hz, 3H) (2H, under H_2_O peak, OH not visible); ^13^C NMR (101 MHz, DMSO-*d*_6_) δ ^13^C NMR (101 MHz, DMSO) δ 180.5, 177.5, 169.0, 163.9, 161.3, 156.9, 152.6, 151.8, 144.3, 141.2, 128.1, 127.5, 125.7, 120.6, 108.0, 106.9, 66.1, 56.3, 53.3, 47.1, 29.5, 27.7, 13.8, 10.8; LC/MS *m*/*z* calculated for C_39_H_39_N_5_NaO_8_ [M + Na]^+^: 728.2, found 728.1, *t*_R_ = 2.90 min; [α]^24.5 °C^_D_ = +266 (0.03; MeOH).

### (9*H*-Fluoren-9-yl)methyl(*R*)-(3-((2-(2-hydroxy-3-((2-((1-(5-methylfuran-2-yl)propyl)amino)-3,4-dioxocyclobut-1-en-1-yl)amino)benzamido)ethyl)amino)-3-oxopropyl)carbamate (**10e**)

The title compound was synthesized following general procedure 1 using (9*H*-fluoren-9-yl)methyl (3-((2-(3-((2-ethoxy-3,4-dioxocyclobut-1-en-1-yl)amino)-2-hydroxybenzamido)ethyl)amino)-3-oxopropyl)carbamate (**9e**) (40 mg, 0,065 mmol). Purification by flash column chromatography (MeOH/CH_2_Cl_2_, 3:97) gave the title compound (11 mg, 24%) as a yellow oil (purity 95%). ^1^H NMR (400 MHz, DMSO-*d*_6_) δ 13.91 (s, 1H), 9.34 (s, 1H), 9.05 (t, *J* = 5.5 Hz, 1H), 8.69 (d, *J* = 9.0 Hz, 1H), 8.06 (t, *J* = 5.8 Hz, 1H), 8.02–7.94 (m, 1H), 7.88 (d, *J* = 7.5 Hz, 2H), 7.67 (d, *J* = 7.7 Hz, 2H), 7.54–7.47 (m, 2H), 7.43–7.36 (m, 2H), 7.35–7.26 (m, 2H), 6.87 (t, *J* = 8.0 Hz, 1H), 6.26 (d, *J* = 3.1 Hz, 1H), 6.05 (dd, *J* = 3.1, 1.2 Hz, 1H), 5.13 (q, *J* = 7.9 Hz, 1H), 4.36–4.07 (m, 3H), 3.46–3.12 (m, 6H + H_2_O), 2.26 (m, 5H), 2.03–1.75 (m, 2H), 0.92 (t, *J* = 7.3 Hz, 3H);^13^C NMR (101 MHz, DMSO-*d*_6_) δ 184.5, 180.6, 171.0, 170.5, 169.0, 163.6, 156.5, 152.5, 151.8, 151.4, 144.3, 141.1, 128.4, 128.0, 127.5, 125.6, 124.8, 123.5, 120.5, 118.5, 114.3, 110.1, 108.0, 106.9, 65.8, 53.3, 47.1, 38.3, 37.5, 36.2, 27.6, 13.8, 10.7; LC/MS *m*/*z* calculated for C_39_H_39_N_5_NaO_8_ [M + Na]^+^: 728.2, found 728.2, *t*_R_ = 2.94 min; [α]^24.5 °C^_D_ = +300 (0.03; MeOH).

### (9*H*-Fluoren-9-yl)methyl(*R*)-(3-((2-(2-hydroxy-*N*-methyl-3-((2-((1-(5-methylfuran-2-yl)propyl)amino)-3,4-dioxocyclobut-1-en-1-yl)amino)benzamido)ethyl)amino)-3-oxopropyl)carbamate (**10f**)

The title compound was synthesized following general procedure 1 using (9*H*-fluoren-9-yl)methyl(3-((2-(3-((2-ethoxy-3,4-dioxocyclobut-1-en-1-yl)amino)-2-hydroxy-*N*-methylbenzamido)ethyl)amino)-3-oxopropyl)carbamate (**9f**) (17 mg, 0,027 mmol). Purification by flash column chromatography (MeOH/CH_2_Cl_2_, 3:97) gave the title compound (5 mg, 26%) as a yellow oil (purity 95%). ^1^H NMR (400 MHz, DMSO-*d*_6_)δ 9.76 (s, 1H), 9.29 (s, 1H), 8.67 (d, *J* = 9.0 Hz, 1H), 7.88 (d, *J* = 7.5 Hz, 2H), 7.79 (d, *J* = 8.4 Hz, 1H), 7.66 (d, *J* = 7.5 Hz, 2H), 7.40 (td, *J* = 7.5, 1.1 Hz, 2H), 7.35–7.23 (m, 3H), 6.84 (s, 2H), 6.25 (d, *J* = 3.1 Hz, 1H), 6.13–5.92 (m, 1H), 5.14 (q, *J* = 7.7 Hz, 1H), 4.32–4.12 (m, 3H), 3.17 (br s, 3H), 2.92 (s, 3H), 2.25 (br s, 5H), 2.02–1.77 (m, 2H), 1.23 (s, 1H), 0.91 (t, *J* = 7.3 Hz, 3H) (1H, under H_2_O, OH not visible); ^13^C NMR (101 MHz, DMSO-*d*_6_) δ 184.3, 180.7, 169.0, 163.9, 156.4, 152.5, 151.8, 144.3, 141.1, 129.2, 128.0, 127.5, 125.6, 122.6, 120.5, 117.7, 108.0, 106.9, 104.8, 65.8, 53.2, 47.1, 37.4, 27.6, 13.8, 10.7; LC/MS *m*/*z* calculated for C_40_H_41_N_5_NaO_8_ [M + Na]^+^: 742.2, found 742.1, *t*_R_ = 2.87 min; [α]^24.5 °C^_D_ = + 300 (0.04; MeOH).

### (*R*,*E*)-*N*-(2-(6-(2-(4-(2-(5,5-Difluoro-7-(thiophen-2-yl)-5*H*-4λ4,5λ4-dipyrrolo[1,2-c:2’,1’-f][1,3,2]diazaborinin-3-yl)vinyl)phenoxy)acetamido)hexanamido)ethyl)-2-hydroxy-3-((2-((1-(5-methylfuran-2-yl)propyl)amino)-3,4-dioxocyclobut-1-en-1-yl)amino)benzamide (**11a**)

The title compound was synthesized following general procedure 3 using (9*H*-fluoren-9-yl)methyl (*R*)-(2-(2-hydroxy-3-((2-((1-(5-methylfuran-2-yl)propyl)amino)-3,4-dioxocyclobut-1-en-1-yl)amino)benzamido)ethyl)carbamate (**10a**) (2 mg, 0.0031 mmol). Purification (PTLC, Si, MeOH/CH_2_Cl_2_ 5:95) gave the title compound (2 mg, 69%). HRMS (TOF ES^+^) calculated for C_50_H_50_BF_2_N_7_NaO_8_S [M + Na]^+^: 980.3394, found 980.3260.

### (*R*,*E*)-*N*-(2-(2-(6-(2-(4-(2-(5,5-Difluoro-7-(thiophen-2-yl)-5*H*-4λ4,5λ4-dipyrrolo[1,2-c:2′,1′-f][1,3,2]diazaborinin-3-yl)vinyl)phenoxy)acetamido)hexanamido)acetamido)ethyl)-2-hydroxy-3-((2-((1-(5-methylfuran-2-yl)propyl)amino)-3,4-dioxocyclobut-1-en-1-yl)amino)benzamide (**11c**)

The title compound was synthesized following general procedure 3 using (9*H*-fluoren-9-yl)methyl (*R*)-(2-((2-(2-hydroxy-3-((2-((1-(5-methylfuran-2-yl)propyl)amino)-3,4-dioxocyclobut-1-en-1-yl)amino)benzamido)ethyl)amino)-2-oxoethyl)carbamate (**10c**) (2 mg, 0.0029 mmol). Purification (PTLC, Si, MeOH/5:95) gave the title compound (2 mg, 69%). HRMS (TOF ES^+^) calculated for C_52_H_53_BF_2_N_8_NaO_9_S [M + Na]^+^: 1037.3609, found 1037.3273.

### (*R*,*E*)*-N*-(2-(2-(6-(2-(4-(2-(5,5-Difluoro-7-(thiophen-2-yl)-5*H*-4λ4,5λ4-dipyrrolo[1,2-c:2′,1′-f][1,3,2]diazaborinin-3-yl)vinyl)phenoxy)acetamido)hexanamido)acetamido)ethyl)-2-hydroxy-*N*-methyl-3-((2-((1-(5-methylfuran-2-yl)propyl)amino)-3,4-dioxocyclobut-1-en-1-yl)amino)benzamide (**11d**)

The title compound was synthesized following general procedure 3 using (9*H*-fluoren-9-yl)methyl (*R*)-(2-((2-(2-hydroxy-*N*-methyl-3-((2-((1-(5-methylfuran-2-yl)propyl)amino)-3,4-dioxocyclobut-1-en-1-yl)amino)benzamido)ethyl)amino)-2-oxoethyl)carbamate (**10d**) (1.1 mg, 0.0015 mmol). Purification (PTLC, Si, MeOH/CH_2_Cl_2_ 5:95) gave the title compound (0.5 mg, 34%). HRMS (TOF ES^+^) calculated for C_53_H_55_BF_2_N_8_NaO_9_S [M + Na]^+^: 1051.3766, found 1051.3648.

### (*R*,*E*)*-N*-(2-(3-(6-(2-(4-(2-(5,5-Difluoro-7-(thiophen-2-yl)-5*H*-4λ4,5λ4-dipyrrolo[1,2-c:2′,1′-f][1,3,2]diazaborinin-3-yl)vinyl)phenoxy)acetamido)hexanamido)propanamido)ethyl)-2-hydroxy-3-((2-((1-(5-methylfuran-2-yl)propyl)amino)-3,4-dioxocyclobut-1-en-1-yl)amino)benzamide (**11e**)

The title compound was synthesized following general procedure 3 using (9*H*-fluoren-9-yl)methyl (*R*)-(3-((2-(2-hydroxy-3-((2-((1-(5-methylfuran-2-yl)propyl)amino)-3,4-dioxocyclobut-1-en-1-yl)amino)benzamido)ethyl)amino)-3-oxopropyl)carbamate (**10e**) (1 mg, 0.0014 mmol). Purification (PTLC, Si, MeOH/CH_2_Cl_2_ 5:95) gave the title compound (0.9 mg, 63%). HRMS (TOF ES^+^) calculated for C_53_H_55_BF_2_N_8_NaO_9_S [M + Na]^+^: 1051.3766, found 1051.3630.

### (*R*,*E*)*-N*-(2-(3-(6-(2-(4-(2-(5,5-Difluoro-7-(thiophen-2-yl)-5*H*-4λ4,5λ4-dipyrrolo[1,2-c:2′,1′-f][1,3,2]diazaborinin-3-yl)vinyl)phenoxy)acetamido)hexanamido)propanamido)ethyl)-2-hydroxy-*N*-methyl-3-((2-((1-(5-methylfuran-2-yl)propyl)amino)-3,4-dioxocyclobut-1-en-1-yl)amino)benzamide (**11f**)

The title compound was synthesized following general procedure 3 using (9*H*-fluoren-9-yl)methyl (*R*)-(3-((2-(2-hydroxy-*N*-methyl-3-((2-((1-(5-methylfuran-2-yl)propyl)amino)-3,4-dioxocyclobut-1-en-1-yl)amino)benzamido)ethyl)amino)-3-oxopropyl)carbamate (**10f**) (1 mg, 0.0013 mmol). Purification (PTLC, Si, MeOH/CH_2_Cl_2_ 5:95) gave the title compound (0.5 mg, 39%). HRMS (TOF ES^+^) calculated for C_54_H_57_BF_2_N_8_NaO_9_S [M + Na]^+^: 1065.3922, found 1065.3811.

## Supporting Information Available

Molecular formula strings of tested compounds (CSV)
PDB coordinate files for **1** and **2** docked into CXCR2 crystal structure (6LFL) (PDB, PDB)
NanoBRET competition binding curves for **2** and **12** in CXCR1 membranes, LCMS or analytical reverse phase HPLC chromatograms and HRMS spectra for **7a**,**b**, **10a**,**c**, **11a**,**c**–**f**, ROESY spectra of **6a**,**b** (PDF)

## Supplementary Material

jm3c00849_si_001.csv

jm3c00849_si_002.pdb

jm3c00849_si_003.pdb

## ABBREVIATIONS

Boc: *tert*-butyloxycarbonyl
BODIPY: 4,4-difluoro-4-bora-3*a*,4*a*-diaza-*s*-indacene
BRET: bioluminescence resonance energy transfer
BSA: bovine serum albumin
cAMP: cyclic adenosine monophosphate
CCR: CC chemokine receptor
COPD: chronic obstructive pulmonary disease
CX_3_CR: CX_3_C chemokine receptor
CXCR: CXC chemokine receptor
CXCR2: CXC chemokine receptor 2
DAG: diacylglycerol
DIPEA: *N*,*N-*diisopropylethylamine
DMEM: Dulbecco’s modified Eagle’s medium
DMF: *N*,*N-*dimethylformamide
EDC: 1-ethyl-3-(3-dimethylaminopropyl)carbodiimide
FCS: fetal calf bovine serum
Fmoc: fluorenylmethoxycarbonyl protecting group
GDP: guanosine diphosphate
GPCR: G-protein-coupled receptor
GTP: guanosine trisphosphate
HBS: HEPES-buffered saline
HEK: human embryonic kidney
HOBt: hydroxybenzotriazole
HRMS: high-resolution mass spectrometry
IL8: interleukin 8
IP_3_: inositol trisphosphate
LC–MS: liquid chromatography-mass spectrometry
LgBiT: large BiT
Mp: melting point
MPLC: medium pressure liquid chromatography
NAM: negative allosteric modulator
NanoLuc: nanoluciferase
NMR: nuclear magnetic resonance
PDB: protein data bank
PI3K: phospatidylinositol-3-kinase
PKC: protein kinase C
PLC: phospholipase C
Prep: preparative
rt.: room temperature
SmBiT: small BiT
TLC: thin layer chromatography
TM: transmembrane
UV: ultraviolet
