# Supplementary material for: Design, Synthesis, and Application of Fluorescent Ligands Targeting the Intracellular Allosteric Binding Site of the CXC Chemokine Receptor 2
Source: J Med Chem. 2023 Jul 31;66(18):12911–30. doi: 10.1021/acs.jmedchem.3c00849 (PMC10544029; doi:10.1021/acs.jmedchem.3c00849)
Supplement: Supplementary file 4 — jm3c00849_si_004.pdf [file jm3c00849_si_004.pdf]

Supporting Information

Design, synthesis and application of fluorescent ligands targeting the intracellular allosteric binding site of the CXC Chemokine Receptor 2

Bianca Maria Casella[1], James P. Farmer[2], Desislava N. Nesheva[2], Huw E. L. Williams[3], Steven J. Charlton[2], Nicholas D. Holliday[,2], Charles A. Laughton[*,1], Shailesh N. Mistry[*,1]*

1. Division of Biomolecular Sciences and Medicinal Chemistry, School of Pharmacy, University of Nottingham Biodiscovery Institute, Nottingham NG7 2RD UK.

2. Cell signalling, School of Life Sciences, Queen's Medical Centre, University of Nottingham, Nottingham NG7 2UH UK.

3. School of Chemistry, University of Nottingham Biodiscovery Institute, Nottingham NG7 2RD UK

Corresponding Author

*Shailesh N. Mistry Tel: +44-115-8467983. Email shailesh.mistry@nottingham.ac.uk

*Charles A. Laughton Tel: +44-115-9513405. Email charles.laughton@nottingham.ac.uk

*Nicholas D. Holliday Tel:_+44-115-8230081. Email nicholas.holliday@nottingham.ac.uk

Table of contents

Figure S1. NanoBRET competition binding studies in CXCR1 membranes **S3**

Figure S2. LCMS long method chromatogram for compound **7a** **S4**

Figure S3. HRMS for compound **7a** **S5**

Figure S4. Analytical RP-HPLC chromatogram for compound **7b** **S6**

Figure S5. HRMS for compound **7b** **S6**

Figure S6. Analytical RP-HPLC chromatogram for compound **10a** **S7**

Figure S7. HRMS for compound **10a** **S7**

Figure S8. Analytical RP-HPLC chromatogram for compound **10c** **S8**

Figure S9. HRMS for compound **10c** **S8**

Figure S10. Analytical RP-HPLC chromatogram for compound **11a** **S9**

Figure S11. HRMS for compound **11a** **S9**

Figure S12. Analytical RP-HPLC chromatogram for compound **11c** **S10**

Figure S13. HRMS for compound **11c** **S10**

Figure S14. Analytical RP-HPLC chromatogram for compound **11d** **S11**

Figure S15. HRMS for compound **11d** **S11**

Figure S16. Analytical RP-HPLC chromatogram for compound **11e** **S12**

Figure S17. HRMS for compound **11e** **S12**

Figure S18. Analytical RP-HPLC chromatogram for compound **11f** **S13**

Figure S19. HRMS for compound **11f** **S13**

Figure S20. ROESY spectra compound **6a** **S14**

Figure S21. ROESY spectra compound **6b** **S15**

Figure S22. Excitation-emission spectra for compounds **11a**, **11c-f** **S16**

Figure S23. Snap-CXCR2 expression within HEK293 stable cell line **S17**

Figure S24. Saturation binding curves in CXCR2 membranes and whole cells and CXCR1 membranes for fluorescent ligands **11a, 11c-f** **S18**

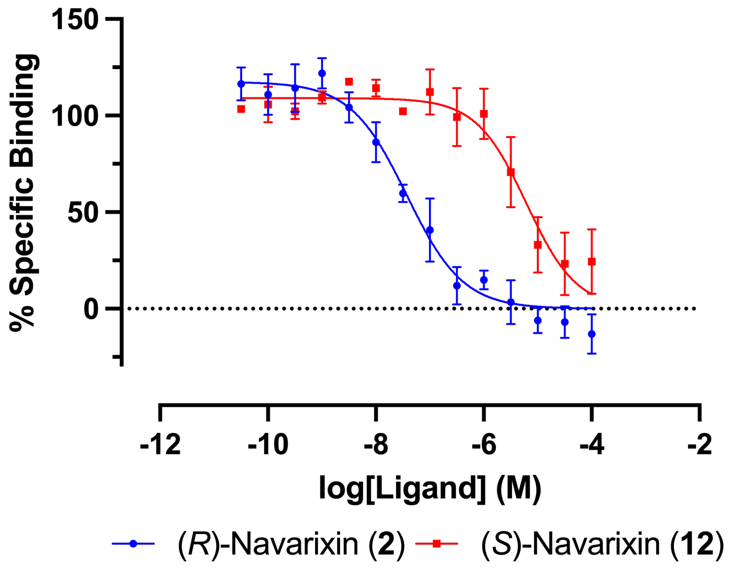

Figure S1. NanoBRET competition binding studies CXCR1 allosteric modulators in CXCR1 membranes. Membranes were incubated with 100 nM **11d** and increasing concentrations of unlabelled ligands for 1-hours at 37°C. The data shown represent the combined mean ± SEM of n = 3 experiments where each experiment was performed in duplicates. (*R*-navarixin (**2**), pK_i = 7.66 ± 0.15; *S*-navarixin (**12**), pK_i = 5.59 ± 0.18).

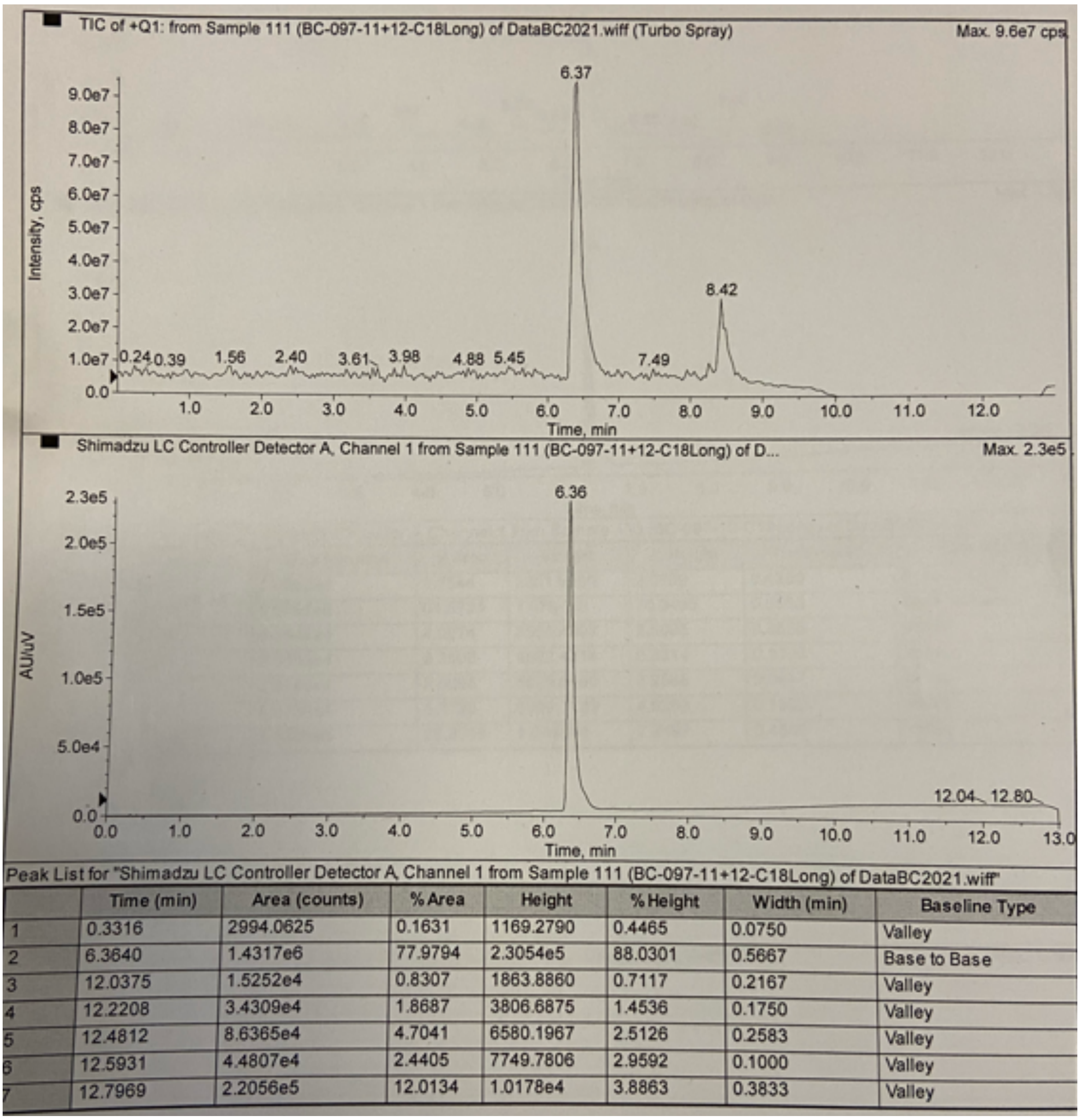

Peak List for "Shimadzu LC Controller Detector A, Channel 1 from Sample 111 (BC-097-11+12-C18Long) of DataBC2021.wiff"

	Time (min)	Area (counts)	% Area	Height	% Height	Width (min)	Baseline Type
1	0.3316	2994.0625	0.1631	1169.2790	0.4465	0.0750	Valley
2	6.3640	1.4317e6	77.9794	2.3054e5	88.0301	0.5667	Base to Base
3	12.0375	1.5252e4	0.8307	1863.8860	0.7117	0.2167	Valley
4	12.2208	3.4309e4	1.8687	3806.6875	1.4536	0.1750	Valley
5	12.4812	8.6365e4	4.7041	6580.1967	2.5126	0.2583	Valley
6	12.5931	4.4807e4	2.4405	7749.7806	2.9592	0.1000	Valley
7	12.7969	2.2056e5	12.0134	1.0178e4	3.8863	0.3833	Valley

Figure S2. LCMS long method chromatogram for compound **7a.**

Sample-ID	paxbc1_BC097	Lab	C13
Submitter	Bianca Casella (paxbc1)	Supervisor	
Analysis Name	paxbc1_BC097_619286_14_01_103908.d	Acquisition Date	8/2/2021 1:33:46 PM
Ionisation Mode	ESI Positive	Instrument	Bruker MicroTOF

+MS, 0.7-0.9min #43-54

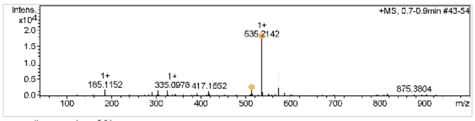

#	m/z	I %
1	185.1152	10.4
2	282.2817	2.9
3	291.1093	6.0
4	304.2611	9.0
5	325.1614	9.4
6	335.0978	10.2
7	391.1603	5.2
8	417.1652	7.5
9	419.1655	2.8
10	513.2315	9.8
11	514.2377	3.0
12	535.2142	100.0
13	536.2190	31.3
14	537.2190	6.6
15	551.1954	2.9
16	557.1962	3.6
17	572.2967	40.9
18	573.3017	13.1
19	574.3042	3.0
20	586.3210	4.7

Generate Molecular Formula Parameters

Charge	Tolerance	sigma limit	H/C Ratio	Electron Conf.	Nitrogen Rule	Chrom.BackGround	Calibration
+1	6 ppm	0.08	3 - 0	both	false	false	TRUE

Expected Formula C26 H32 N4 O7 **Adduct(s):** H, Na, NH4, C3H5N2, radical

#	meas. m/z	theo. m/z	|Err|[ppm]	Sigma	Formula	Adduct	Adduct Mass
1	513.2315	513.2344	5.70	0.0096	C26H33N4O7	M+H	1.0078
1	535.2142	535.2163	4.00	0.0065	C26H32N4NaO7	M+Na	22.9898

Note: Sigma fits < 0.05 indicates high probability of correct MF

Analysis Name	paxbc1_BC097_619286_14_01_103908.d	The School of Chemistry
Bruker Compass DataAnalysis 4.2	printed: 8/2/2021 1:35:58 PM	Page 1 of 1

Sample-ID	paxbc1_BC097	Lab	C13
Submitter	Bianca Casella (paxbc1)	Supervisor	
Analysis Name	paxbc1_BC097_619286_14_01_103907.d	Acquisition Date	8/2/2021 1:28:01 PM
Ionisation Mode	ESI Negative	Instrument	Bruker MicroTOF

-MS, 0.7-0.9min #59-75

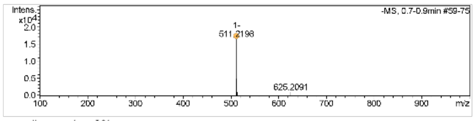

#	m/z	I %
1	511.2198	100.0
2	512.2218	30.3
3	513.2248	6.5
4	625.2091	6.0

Generate Molecular Formula Parameters

Charge	Tolerance	sigma limit	H/C Ratio	Electron Conf.	Nitrogen Rule	Chrom.BackGround	Calibration
-1	6 ppm	0.08	3 - 0	both	false	false	TRUE

Expected Formula C26 H32 N4 O7 **Adduct(s):** H, radical

#	meas. m/z	theo. m/z	|Err|[ppm]	Sigma	Formula	Adduct	Adduct Mass
1	511.2198	511.2198	0.00	0.0054	C26H31N4O7	M- H	1.0078

Note: Sigma fits < 0.05 indicates high probability of correct MF

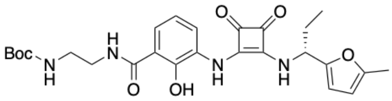

7a

Analysis Name	paxbc1_BC097_619286_14_01_103907.d	The School of Chemistry
Bruker Compass DataAnalysis 4.2	printed: 8/2/2021 1:30:14 PM	Page 1 of 1

Figure S3. HRMS for compound **7a**

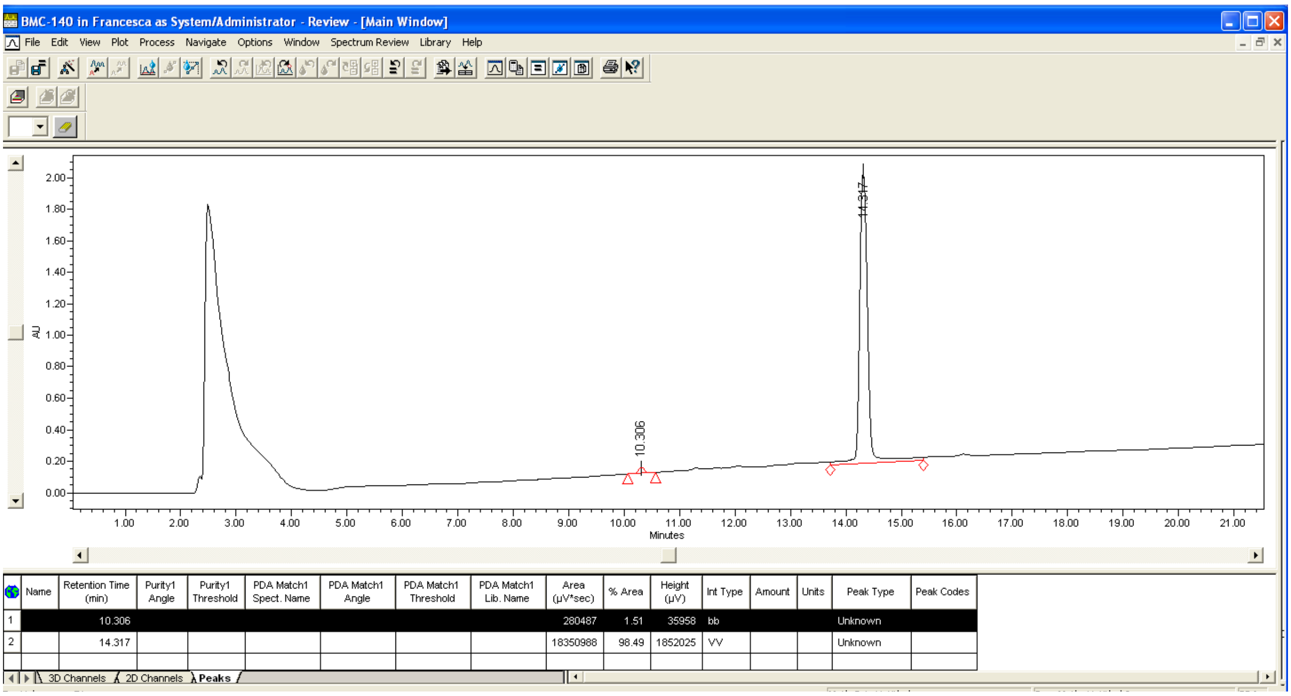

	Name	Retention Time (min)	Purity1 Angle	Purity1 Threshold	PDA Match1 Spect. Name	PDA Match1 Angle	PDA Match1 Threshold	PDA Match1 Lib. Name	Area (µV*sec)	% Area	Height (µV)	Int Type	Amount	Units	Peak Type	Peak Codes
1		10.306							280487	1.51	35958	bb			Unknown	
2		14.317							18350988	98.49	1852025	VV			Unknown	

Figure S4. Analytical RP-HPLC chromatogram for compound **7b**

Sample-ID	paxbc1_BC140	Lab	C13
Submitter	Bianca Casella (paxbc1)	Supervisor	
Analysis Name	paxbc1_BC140_625761_33_01_108960.d	Acquisition Date	1/18/2022 5:03:04 PM
Ionisation Mode	ESI Positive	Instrument	Bruker MicroTOF

+MS, 0.7-0.9min #42-53

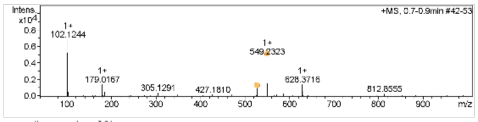

#	m/z	I %
1	100.9999	77.1
2	102.0048	2.6
3	102.1244	100.0
4	102.9967	4.6
5	103.1275	7.3
6	179.0167	20.6
7	185.1168	7.0
8	239.2264	2.9
9	305.1291	5.4
10	427.1810	2.3
11	527.2485	13.6
12	528.2499	4.7
13	549.2323	69.9
14	550.2342	22.3
15	551.2403	5.2
16	586.3225	4.6
17	628.3716	20.8
18	629.3762	8.4
19	812.3646	3.4
20	812.8555	3.6

Generate Molecular Formula Parameters

Charge	Tolerance	sigma limit	H/C Ratio	Electron Conf.	Nitrogen Rule	Chrom.BackGround	Calibration
+1	6 ppm	0.08	3 - 0	both	false	false	TRUE

Expected Formula C27 H34 N4 O7 **Adduct(s):** H, Na, NH4, C3H5N2, radical

#	meas. m/z	theo. m/z	|Err|[ppm]	Sigma	Formula	Adduct	Adduct Mass
1	527.2485	527.2500	2.90	0.0182	C27H35N4O7	M+H	1.0078
1	549.2323	549.2320	0.60	0.0083	C27H34N4NaO7	M+Na	22.9898

Note: Sigma fits < 0.05 indicates high probability of correct MF

Analysis Name paxbc1_BC140_625761_33_01_108960.d The School of Chemistry
Bruker Compass DataAnalysis 4.2 printed: 1/18/2022 5:05:16 PM Page 1 of 1

Sample-ID	paxbc1_BC140	Lab	C13
Submitter	Bianca Casella (paxbc1)	Supervisor	
Analysis Name	paxbc1_BC140_625761_33_01_108961.d	Acquisition Date	1/18/2022 5:08:44 PM
Ionisation Mode	ESI Negative	Instrument	Bruker MicroTOF

-MS, 0.7-0.9min #43-54

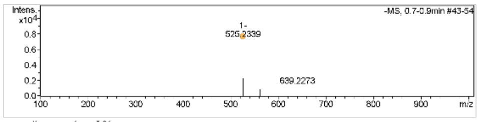

#	m/z	I %
1	525.2339	100.0
2	526.2382	32.2
3	561.2112	12.3
4	639.2273	18.3

Generate Molecular Formula Parameters

Charge	Tolerance	sigma limit	H/C Ratio	Electron Conf.	Nitrogen Rule	Chrom.BackGround	Calibration
-1	6 ppm	0.08	3 - 0	both	false	false	TRUE

Expected Formula C27 H34 N4 O7 **Adduct(s):** H, radical

#	meas. m/z	theo. m/z	|Err|[ppm]	Sigma	Formula	Adduct	Adduct Mass
1	525.2339	525.2355	2.90	0.0315	C27H33N4O7	M- H	1.0078

Note: Sigma fits < 0.05 indicates high probability of correct MF

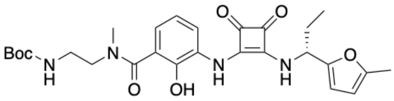

7b

Analysis Name paxbc1_BC140_625761_33_01_108961.d The School of Chemistry
Bruker Compass DataAnalysis 4.2 printed: 1/18/2022 5:10:54 PM Page 1 of 1

Figure S5. HRMS for compound **7b**

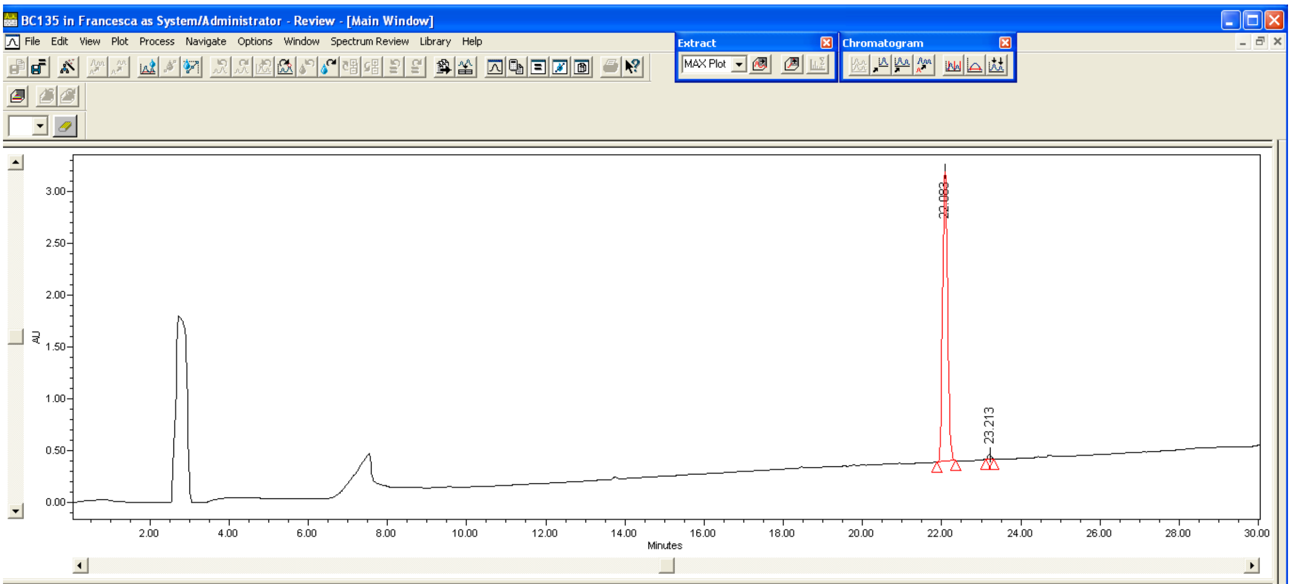

	Name	Retention Time (min)	Purity1 Angle	Purity1 Threshold	PDA Match1 Spect. Name	PDA Match1 Angle	PDA Match1 Threshold	PDA Match1 Lib. Name	Area (µV*sec)	% Area	Height (µV)	Int Type	Amount	Units	Peak Type	Peak Codes
1		22.083	4.626	7.665					25059137	99.00	2812865	BB			Unknown	
2		23.213							253028	1.00	43551	BB			Unknown	P02

Figure S6. Analytical RP-HPLC chromatogram for compound **10a**

Sample-ID	paxbc1_BC135	Lab	C13
Submitter	Bianca Casella (paxbc1)	Supervisor	
Analysis Name	paxbc1_BC135_619287_13_01_103904.d	Acquisition Date	8/2/2021 1:17:26 PM
Ionisation Mode	ESI Positive	Instrument	Bruker MicroTOF

+MS, 0.7-0.9min #43-54

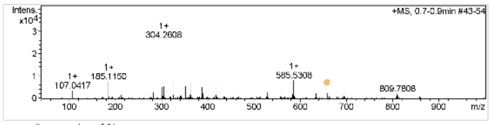

#	m/z	I %
1	107.0417	13.7
2	185.1150	29.4
3	282.2795	10.2
4	302.2452	20.0
5	304.2608	100.0
6	305.2644	21.4
7	325.1609	31.4
8	352.2469	21.8
9	363.1558	27.2
10	388.3121	20.2
11	389.3145	8.1
12	417.1652	26.1
13	419.1602	9.1
14	528.5101	10.7
15	585.5308	30.8
16	586.5361	12.7
17	633.5154	9.2
18	657.2286	22.3
19	658.2351	9.7
20	809.7808	7.5

Generate Molecular Formula Parameters

Charge	Tolerance	sigma limit	H/C Ratio	Electron Conf.	Nitrogen Rule	Chrom.BackGround	Calibration
+1	6 ppm	0.08	3 - 0	both	false	false	TRUE

Expected Formula C36 H34 N4 O7 **Adduct(s):** H, Na, NH4, C3H5N2, radical

#	meas. m/z	theo. m/z	|Err|[ppm]	Sigma	Formula	Adduct	Adduct Mass
1	657.2286	657.2320	5.20	0.0157	C36H34N4NaO7	M+Na	22.9898

Note: Sigma fits < 0.05 indicates high probability of correct MF

Sample-ID	paxbc1_BC135	Lab	C13
Submitter	Bianca Casella (paxbc1)	Supervisor	
Analysis Name	paxbc1_BC135_619287_13_01_103906.d	Acquisition Date	8/2/2021 1:25:35 PM
Ionisation Mode	ESI Negative	Instrument	Bruker MicroTOF

-MS, 0.7-0.9min #59-75

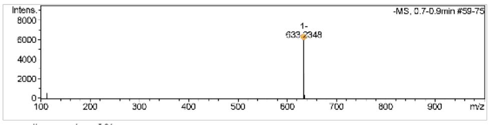

#	m/z	I %
1	112.9853	9.5
2	633.2348	100.0
3	634.2356	43.4
4	635.2363	5.6

Generate Molecular Formula Parameters

Charge	Tolerance	sigma limit	H/C Ratio	Electron Conf.	Nitrogen Rule	Chrom.BackGround	Calibration
-1	6 ppm	0.08	3 - 0	both	false	false	TRUE

Expected Formula C36 H34 N4 O7 **Adduct(s):** H, radical

#	meas. m/z	theo. m/z	|Err|[ppm]	Sigma	Formula	Adduct	Adduct Mass
1	633.2348	633.2355	1.10	0.0249	C36H33N4O7	M- H	1.0078

Note: Sigma fits < 0.05 indicates high probability of correct MF

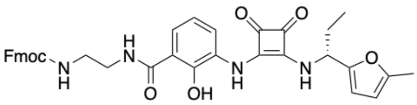

10a

Analysis Name paxbc1_BC135_619287_13_01_103904.d The School of Chemistry
Bruker Compass DataAnalysis 4.2 printed: 8/2/2021 1:19:38 PM Page 1 of 1

Analysis Name paxbc1_BC135_619287_13_01_103906.d The School of Chemistry
Bruker Compass DataAnalysis 4.2 printed: 8/2/2021 1:27:47 PM Page 1 of 1

Figure S7. HRMS for compound **10a**

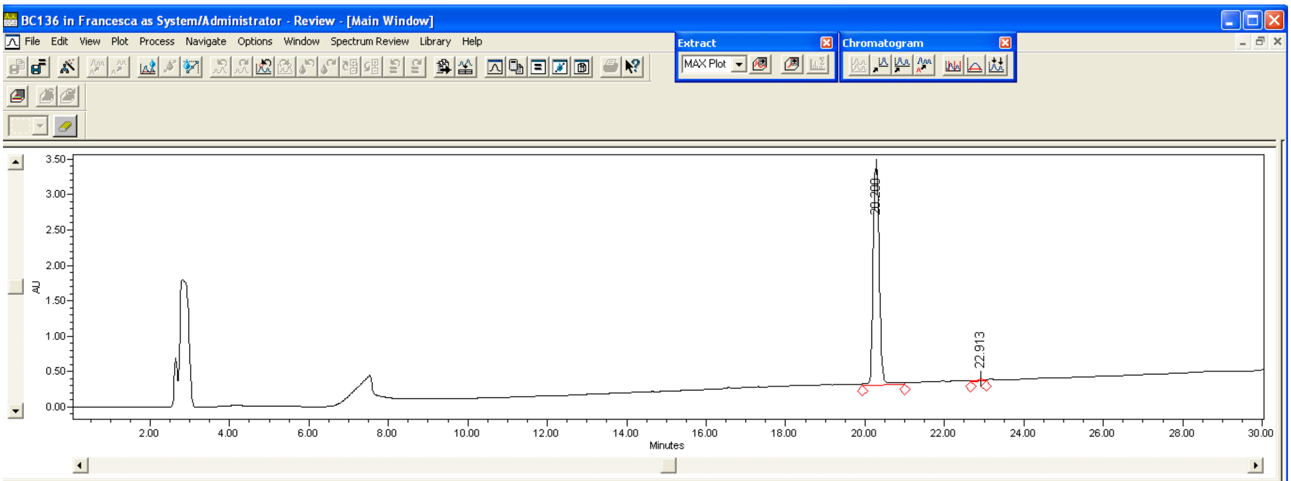

	Name	Retention Time (min)	Purity1 Angle	Purity1 Threshold	PDA Match1 Spect. Name	PDA Match1 Angle	PDA Match1 Threshold	PDA Match1 Lib. Name	Area (µV*sec)	% Area	Height (µV)	Int Type	Amount	Units	Peak Type	Peak Codes
1		20.280							34172685	98.79	3083070	VV			Unknown	
2		22.913							420156	1.21	37514	VV			Unknown	

3D Channels / 2D Channels / Peaks

Figure S8. Analytical RP-HPLC chromatogram for compound **10c**

Sample-ID	paxbc1_BC136	Lab	C13
Submitter	Bianca Casella (paxbc1)	Supervisor	
Analysis Name	paxbc1_BC136_619290_12_01_103903.d	Acquisition Date	8/2/2021 1:14:57 PM
Ionisation Mode	ESI Positive	Instrument	Bruker MicroTOF

+MS, 0.7-0.9min #43-54

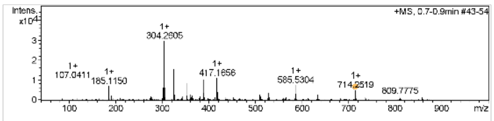

#	m/z	I %
1	107.0411	33.7
2	185.1150	23.7
3	302.2449	20.8
4	304.2605	100.0
5	305.2649	20.8
6	325.1613	51.8
7	326.1663	9.2
8	352.2462	28.3
9	361.1424	9.8
10	363.1553	31.6
11	388.3121	34.8
12	389.3147	11.5
13	417.1656	37.6
14	419.1623	13.2
15	509.1683	8.8
16	528.5106	11.8
17	585.5304	25.6
18	586.5342	10.3
19	633.5164	9.1
20	714.2519	17.2

Generate Molecular Formula Parameters

Charge	Tolerance	sigma limit	H/C Ratio	Electron Conf.	Nitrogen Rule	Chrom.BackGround	Calibration
+1	6 ppm	0.08	3 - 0	both	false	false	TRUE

Expected Formula C38 H37 N5 O8 **Adduct(s):** H, Na, NH4, C3H5N2, radical

#	meas. m/z	theo. m/z	|Err|[ppm]	Sigma	Formula	Adduct	Adduct Mass
1	714.2519	714.2534	2.10	0.0153	C38H37N5NaO8	M+Na	22.9898

Note: Sigma fits < 0.05 indicates high probability of correct MF

Analysis Name	paxbc1_BC136_619290_12_01_103903.d		The School of Chemistry
Bruker Compass DataAnalysis 4.2	printed: 8/2/2021 1:17:09 PM		Page 1 of 1

Sample-ID	paxbc1_BC136	Lab	C13
Submitter	Bianca Casella (paxbc1)	Supervisor	
Analysis Name	paxbc1_BC136_619290_12_01_103905.d	Acquisition Date	8/2/2021 1:23:07 PM
Ionisation Mode	ESI Negative	Instrument	Bruker MicroTOF

-MS, 0.7-0.9min #59-75

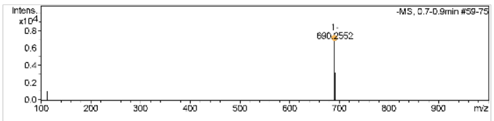

#	m/z	I %
1	112.9852	15.8
2	690.2552	100.0
3	691.2587	47.2
4	692.2628	11.8

Generate Molecular Formula Parameters

Charge	Tolerance	sigma limit	H/C Ratio	Electron Conf.	Nitrogen Rule	Chrom.BackGround	Calibration
-1	6 ppm	0.08	3 - 0	both	false	false	TRUE

Expected Formula C38 H37 N5 O8 **Adduct(s):** H, radical

#	meas. m/z	theo. m/z	|Err|[ppm]	Sigma	Formula	Adduct	Adduct Mass
1	690.2552	690.2569	2.60	0.0208	C38H36N5O8	M- H	1.0078

Note: Sigma fits < 0.05 indicates high probability of correct MF

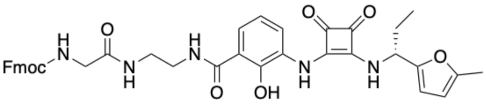

10c

Analysis Name	paxbc1_BC136_619290_12_01_103905.d		The School of Chemistry
Bruker Compass DataAnalysis 4.2	printed: 8/2/2021 1:25:22 PM		Page 1 of 1

Figure S9. HRMS for compound **10c**

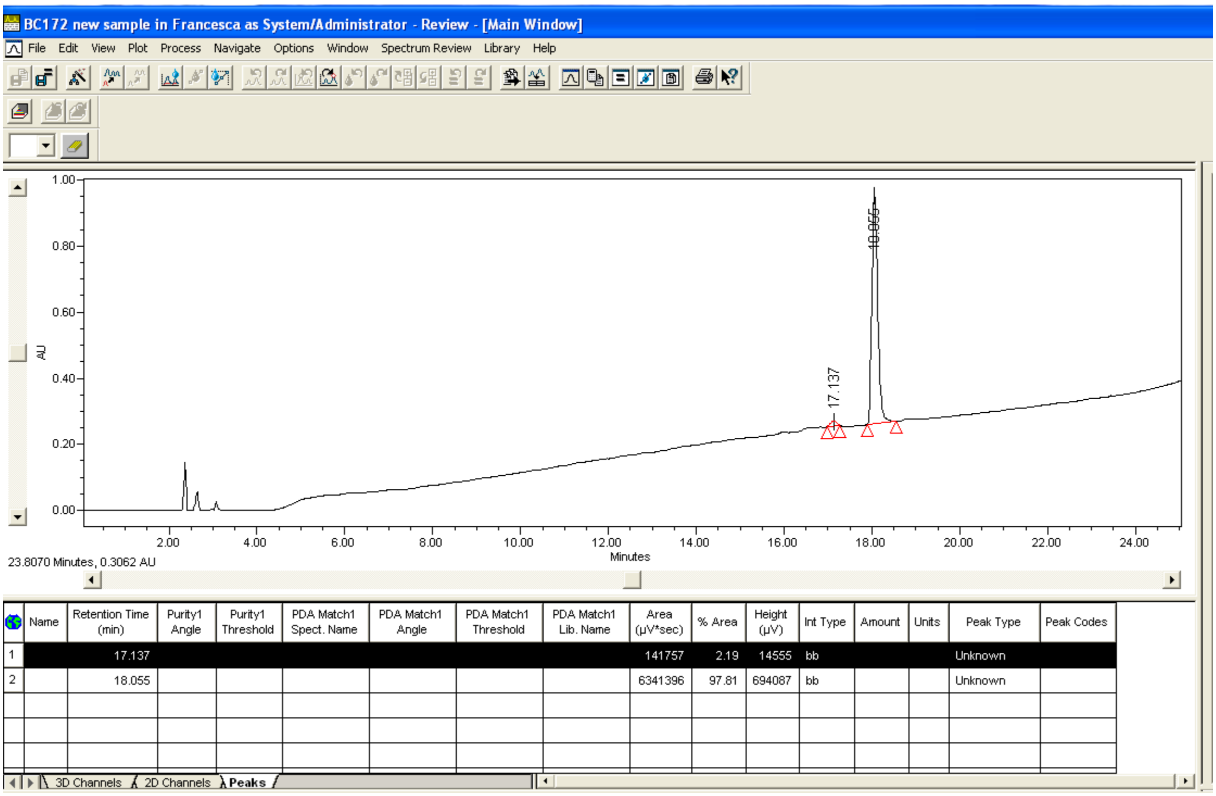

	Name	Retention Time (min)	Purity1 Angle	Purity1 Threshold	PDA Match1 Spect. Name	PDA Match1 Angle	PDA Match1 Threshold	PDA Match1 Lib. Name	Area (µV*sec)	% Area	Height (µV)	Int Type	Amount	Units	Peak Type	Peak Codes
1		17.137							141757	2.19	14555	bb			Unknown	
2		18.055							6341396	97.81	694087	bb			Unknown	

Figure S10. Analytical RP-HPLC chromatogram for compound **11a**

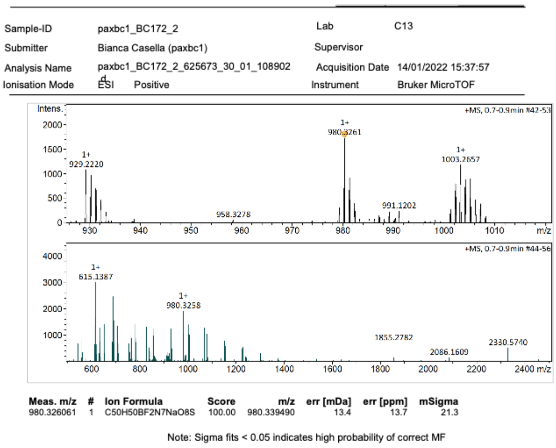

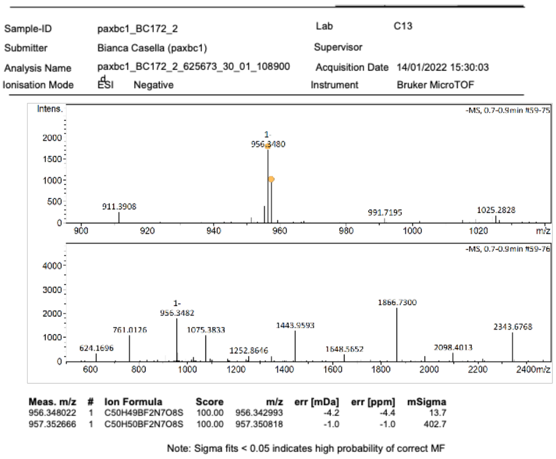

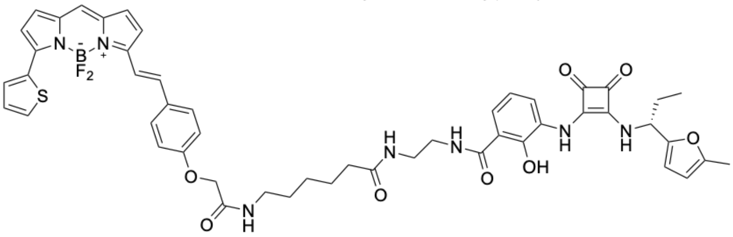

11a

Analysis Name paxbc1_BC172_2_625673_30_01_108902.d The School of Chemistry
Bruker Compass DataAnalysis 4.2 printed: 17/02/2022 17:14:20 Page 1 of 1

Analysis Name paxbc1_BC172_2_625673_30_01_108900.d The School of Chemistry
Bruker Compass DataAnalysis 4.2 printed: 17/02/2022 17:23:18 Page 1 of 1

Figure S11. HRMS for compound **11a**

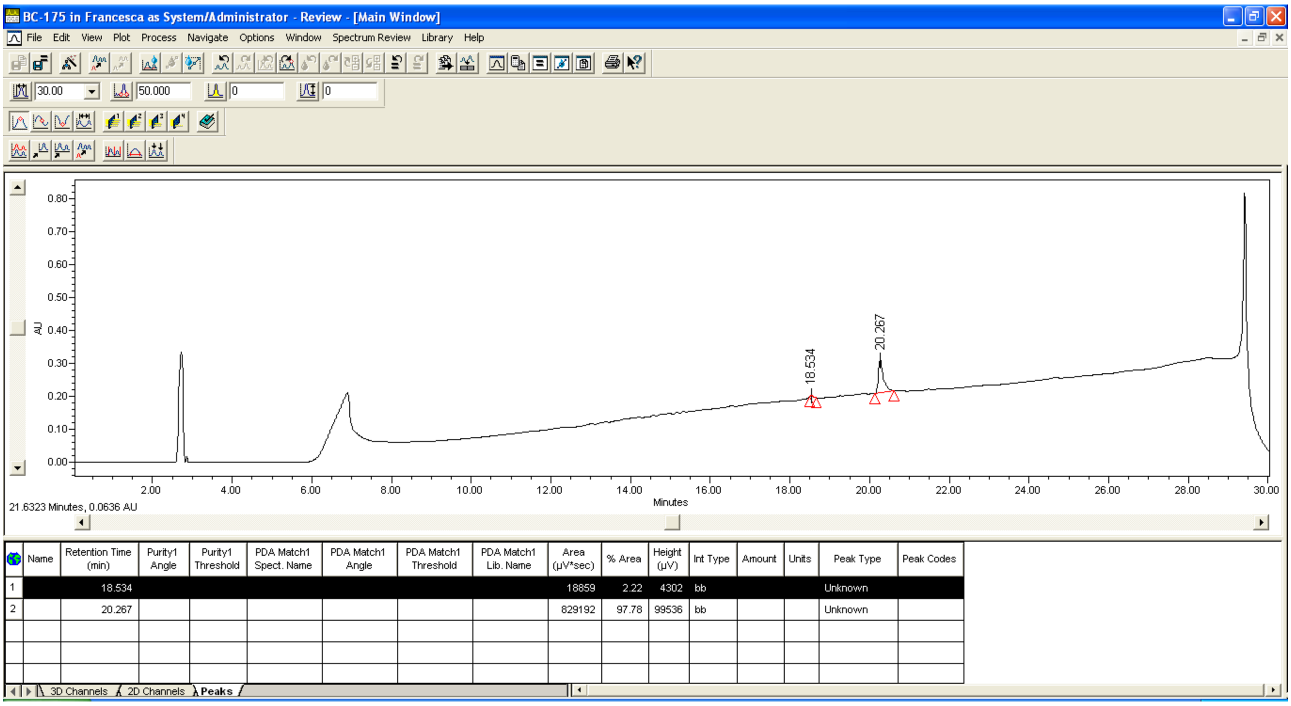

	Name	Retention Time (min)	Purity1 Angle	Purity1 Threshold	PDA Match1 Spect. Name	PDA Match1 Angle	PDA Match1 Threshold	PDA Match1 Lib. Name	Area (µV*sec)	% Area	Height (µV)	Int Type	Amount	Units	Peak Type	Peak Codes
1		18.534							18859	2.22	4302	bb			Unknown	
2		20.267							829192	97.78	99536	bb			Unknown	

Figure S12. Analytical RP-HPLC chromatogram for compound **11c**

Sample-ID	paxbc1_BC175	Lab	C13
Submitter	Bianca Casella (paxbc1)	Supervisor	
Analysis Name	paxbc1_BC175_626974_75_01_109991.d	Acquisition Date	14/02/2022 17:41:10
Ionisation Mode	ESI Positive	Instrument	Bruker MicroTOF

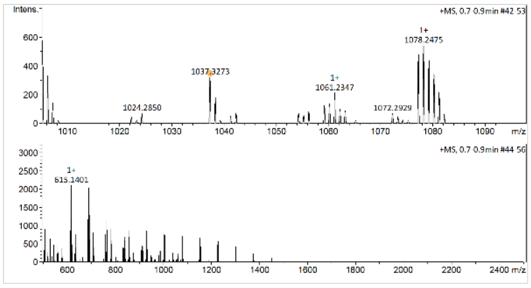

Meas. m/z	#	Ion Formula	Score	m/z	err [mDa]	err [ppm]	mSigma
1037.327302	1	C52H53BF2N8NaO9S	100.00	1037.360954	34.5	33.3	129.5

Note: Sigma fits < 0.05 indicates high probability of correct MF

Sample-ID	paxbc1_BC175	Lab	C13
Submitter	Bianca Casella (paxbc1)	Supervisor	
Analysis Name	paxbc1_BC175_626974_75_01_109994.d	Acquisition Date	14/02/2022 17:51:48
Ionisation Mode	ESI Negative	Instrument	Bruker MicroTOF

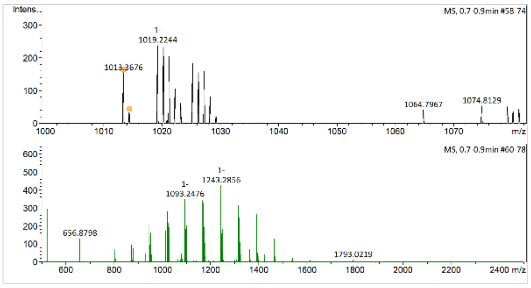

Meas. m/z	#	Ion Formula	Score	m/z	err [mDa]	err [ppm]	mSigma
1013.367626	1	C52H52BF2N8O9S	100.00	1013.364457	-2.3	-2.3	197.6
1014.377712	1	C52H53BF2N8O9S	100.00	1014.372282	-4.6	-4.5	n.a.

Note: Sigma fits < 0.05 indicates high probability of correct MF

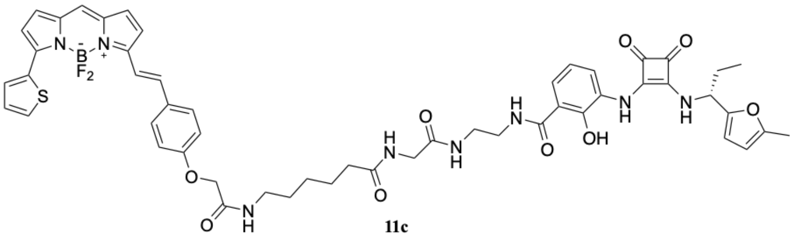

11c

Analysis Name paxbc1_BC175_626974_75_01_109991.d The School of Chemistry
Bruker Compass DataAnalysis 4.2 printed: 17/02/2022 18:03:15 Page 1 of 1

Analysis Name paxbc1_BC175_626974_75_01_109994.d The School of Chemistry
Bruker Compass DataAnalysis 4.2 printed: 17/02/2022 17:53:53 Page 1 of 1

Figure S13. HRMS for compound **11c**

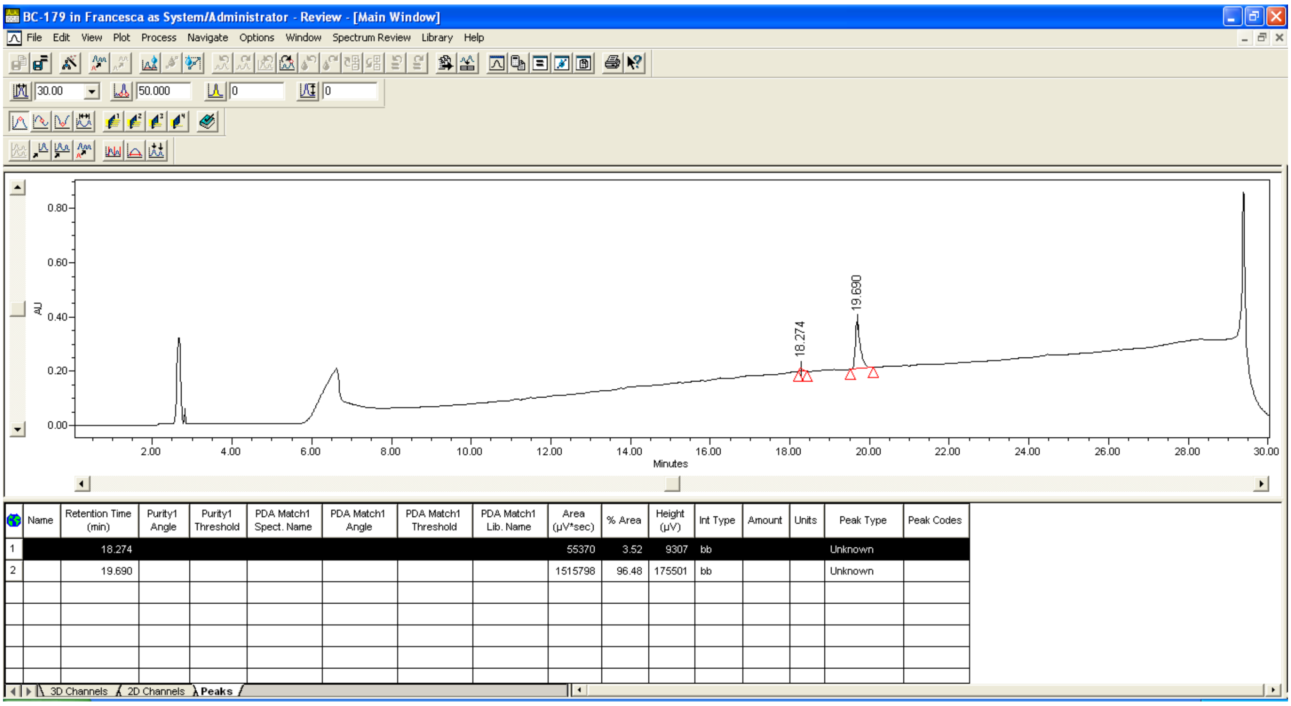

	Name	Retention Time (min)	Purity1 Angle	Purity1 Threshold	PDA Match1 Spect. Name	PDA Match1 Angle	PDA Match1 Threshold	PDA Match1 Lib. Name	Area (µV*sec)	% Area	Height (µV)	Int Type	Amount	Units	Peak Type	Peak Codes
1		18.274							55370	3.52	9307	bb			Unknown	
2		19.690							1515798	96.48	175501	bb			Unknown	

Figure S14. Analytical RP-HPLC chromatogram for compound **11d**

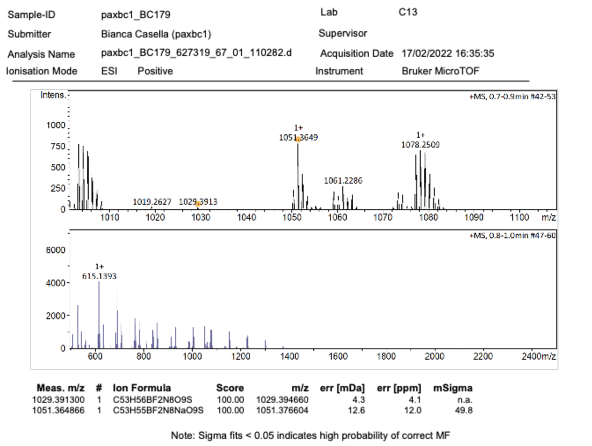

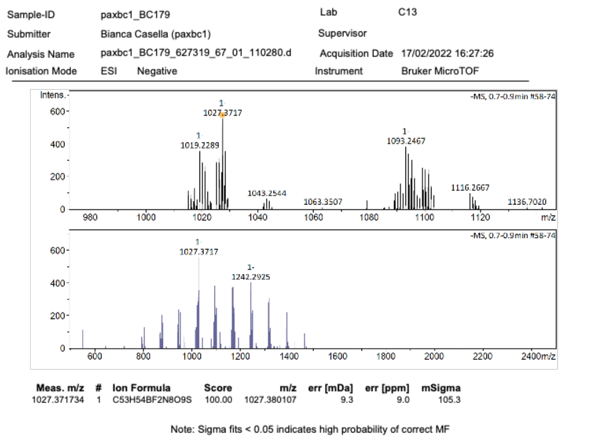

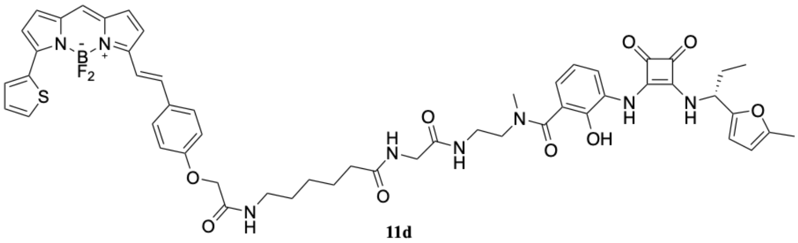

11d

Analysis Name paxbc1_BC179_627319_67_01_110282.d The School of Chemistry
Bruker Compass DataAnalysis 4.2 printed: 28/02/2022 10:25:36 Page 1 of 1

Analysis Name paxbc1_BC179_627319_67_01_110280.d The School of Chemistry
Bruker Compass DataAnalysis 4.2 printed: 28/02/2022 11:10:54 Page 1 of 1

Figure S15. HRMS for compound **11d**

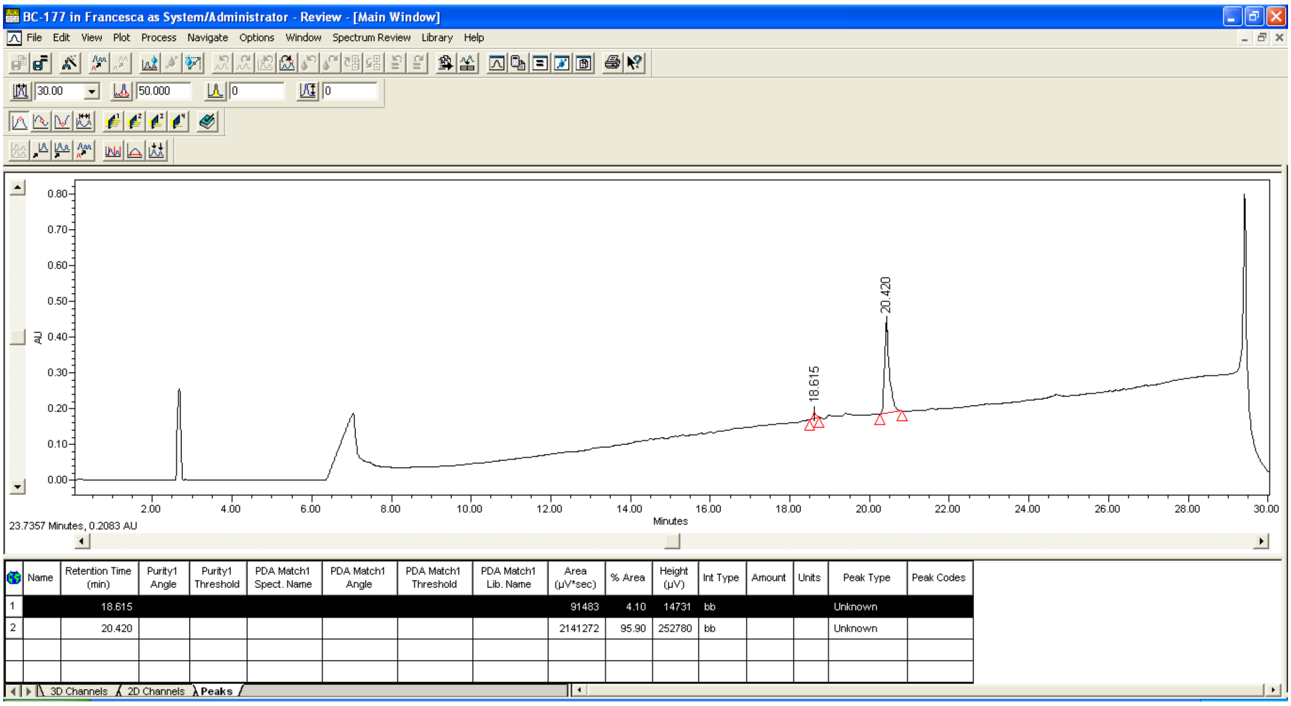

Figure S16. Analytical RP-HPLC chromatogram for compound **11e**

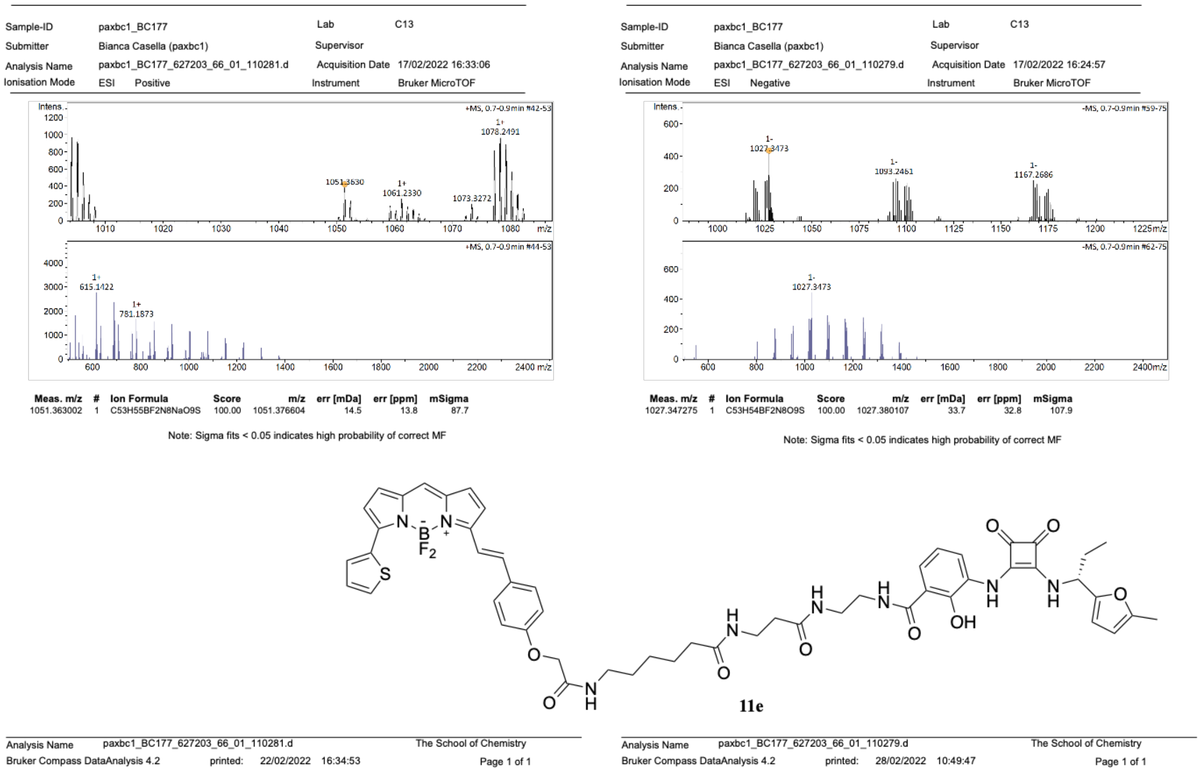

Figure S17. HRMS for compound **11e**

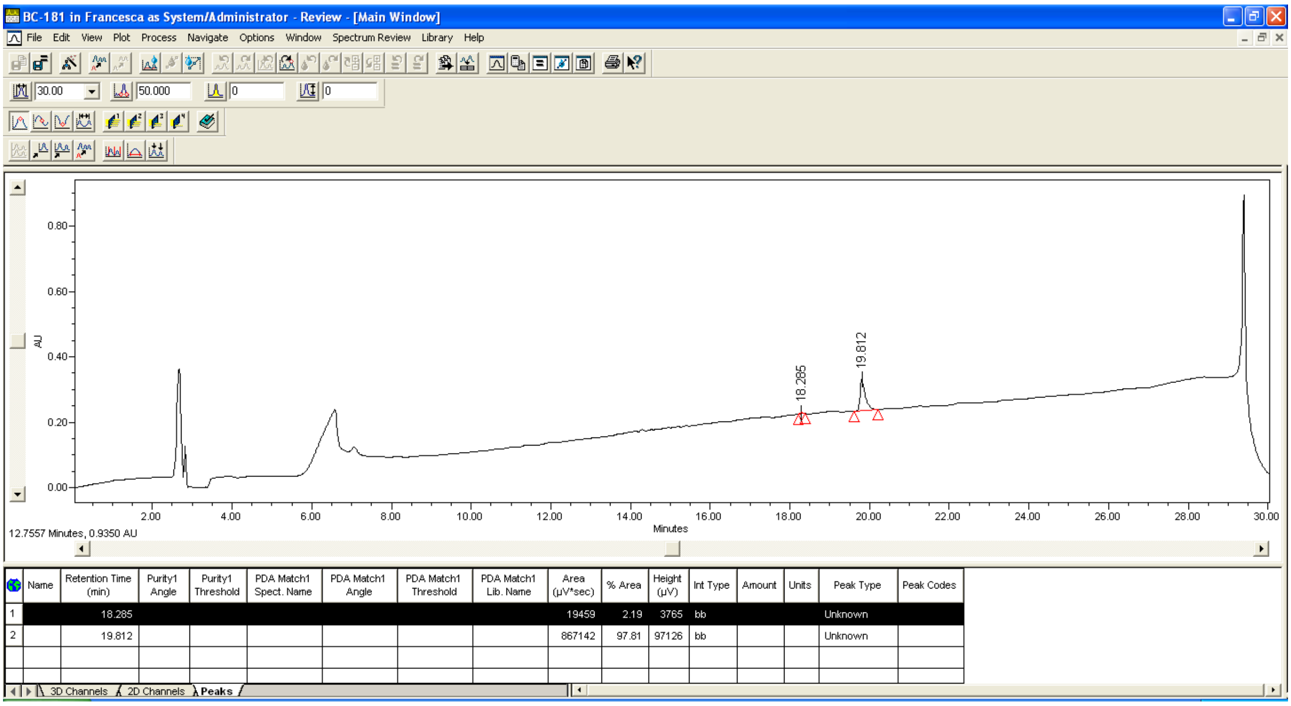

	Name	Retention Time (min)	Purity1 Angle	Purity1 Threshold	PDA Match1 Spect. Name	PDA Match1 Angle	PDA Match1 Threshold	PDA Match1 Lib. Name	Area (µV*sec)	% Area	Height (µV)	Int Type	Amount	Units	Peak Type	Peak Codes
1		18.285							19459	2.19	3765	bb			Unknown	
2		19.812							867142	97.81	97126	bb			Unknown	

Figure S18. Analytical RP-HPLC chromatogram for compound **11f**

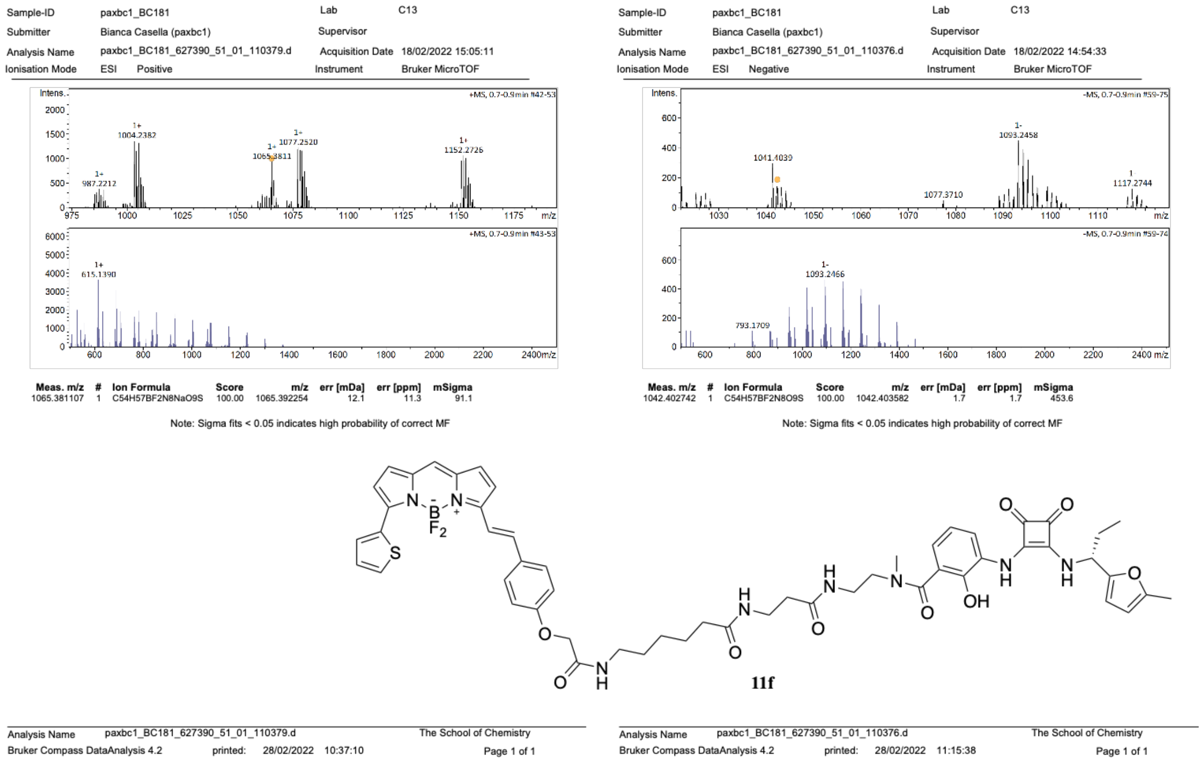

Figure S19. HRMS for compound **11f**

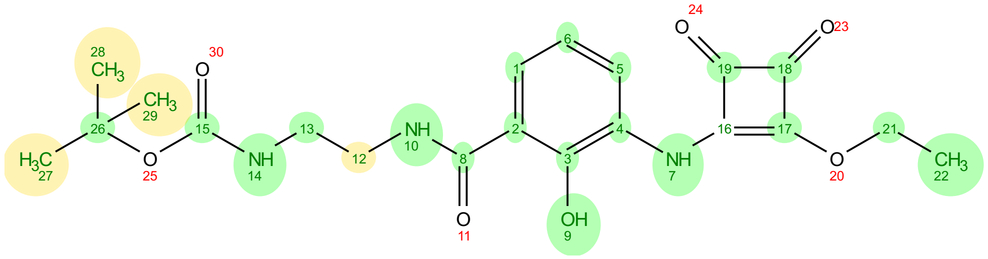

***tert*-Butyl(2-(3-((2-ethoxy-3,4-dioxocyclobut-1-en-1-yl)amino)-2-hydroxybenzamido)ethyl) carbamate (6a).** ^{1}H NMR (500 MHz, DMSO-d_6) δ 13.55 (s, 1H, -O**H**), 10.24 (s, 1H, -N**H-7**), 8.99 (t, J = 5.7 Hz, 1H, -N**H-**10), 7.70 (dd, J = 8.2, 1.5 Hz, 1H, **H**-Ar-1), 7.38 (dd, J = 7.8, 1.4 Hz, 1H, **H**-Ar-5), 6.95 (t, J = 6.0 Hz, 1H, -N**H**-14), 6.88 (t, J = 8.0 Hz, 1H, **H**-Ar-6), 4.67 (q, J = 7.0 Hz, 2H, ethyl-C**H$_2$**), 3.37 – 3.29 (m, 2H, -C**H$_2$**-12, + H_2O), 3.13 (q, J = 6.2 Hz, 2H, -C**H$_2$**-13), 1.26-1.40 (m, 12H, ethyl-C**H$_3$** and 3C**H$_3$** Boc); ^{13}C NMR (101 MHz, DMSO-d_6) δ 188.0 (**C19**), 184.1 (**C18**), 178.1 (**C17**), 170.9 (**C16**), 169.8 (**C8**), 155.7 (**C15**), 154.4 (**C3**), 128.1 (**C5**), 125.9 (**C4**), 124.3 (**C1**), 117.4 (**C6**), 114.6 (**C2**), 77.7 (**C26**), 69.1 (**C21**), 39.1 (**C12**), 38.8 (**C13**), 28.2 (**C27,28,29**), 15.5 (**C22**).

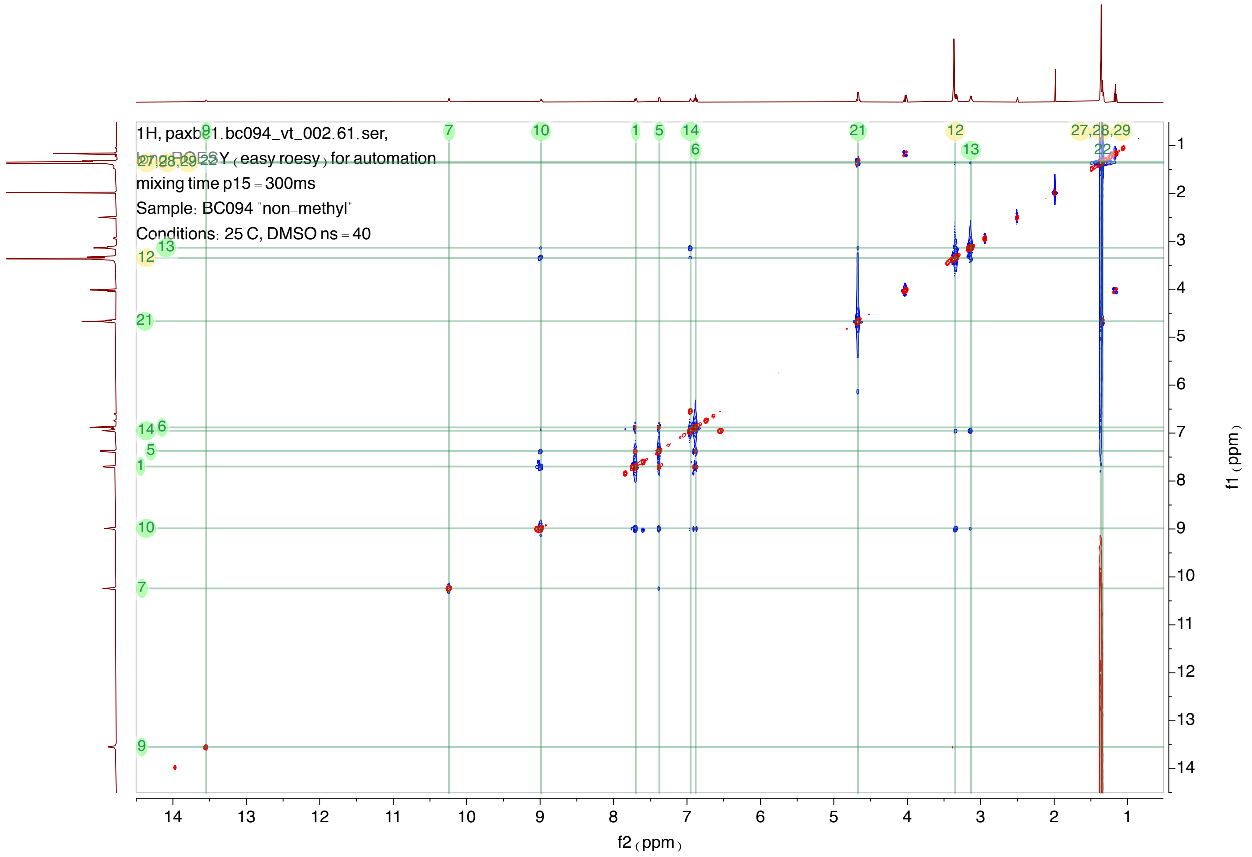

Figure S20. ROESY spectrum of compound **6a**.

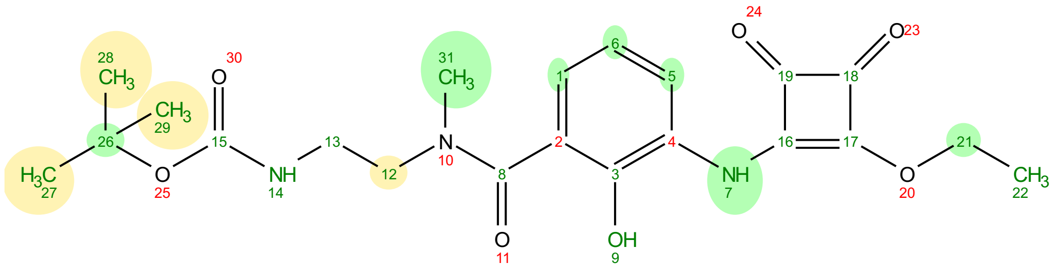

tert-Butyl(2-(3-((2-ethoxy-3,4-dioxocyclobut-1-en-1-yl)amino)-2-hydroxy-_N_-methylbenzamido)ethyl) carbamate (6b). ^{1}H NMR (500 MHz, DMSO-d_6) δ 10.11 (s, 1H, -N**H**7), 9.49 (s, 1H, -O**H**), 7.19 (dd, *J* = 7.8, 1.7 Hz, 1H, **H**-Ar-5), 7.02 (s, 1H, **H**-Ar-1), 6.87 (t, *J* = 7.7 Hz, 2H, **H**-Ar-6 + -N**H**14), 4.65 (q, *J* = 7.1 Hz, 2H, ethyl-C**H$_2$**), 3.43 (t, *J* = 7.0 Hz, 2H, -C**H$_2$**-12 + H_2O), 3.17 (s, 2H, -C**H$_2$**-13), 2.89 (s, 3H, -C**H$_3$**-31), 1.30-1.42 (m, 12H, ethyl-C**H$_3$** and 3C**H$_3$** Boc); ^{13}C NMR (126 MHz, DMSO-d_6) δ 188.2 (**C19**), 184.1 (**C18**), 178.0 (**C17**), 171.2 (**C16**), 168.2 (**C8**), 155.6 (**C15**), 146.7 (**C3**), 126.0 (**C4**), 125.9 (**C2**), 125.6 (**C1**), 125.2 (**C5**), 119.3 (**C6**), 77.7 (**C26**), 69.1 (**C21**), 46.8 (**C12**), 37.5 (**C13**), 36.8 (**C31**), 28.2 (**C27,28,29**), 15.6 (**C22**).

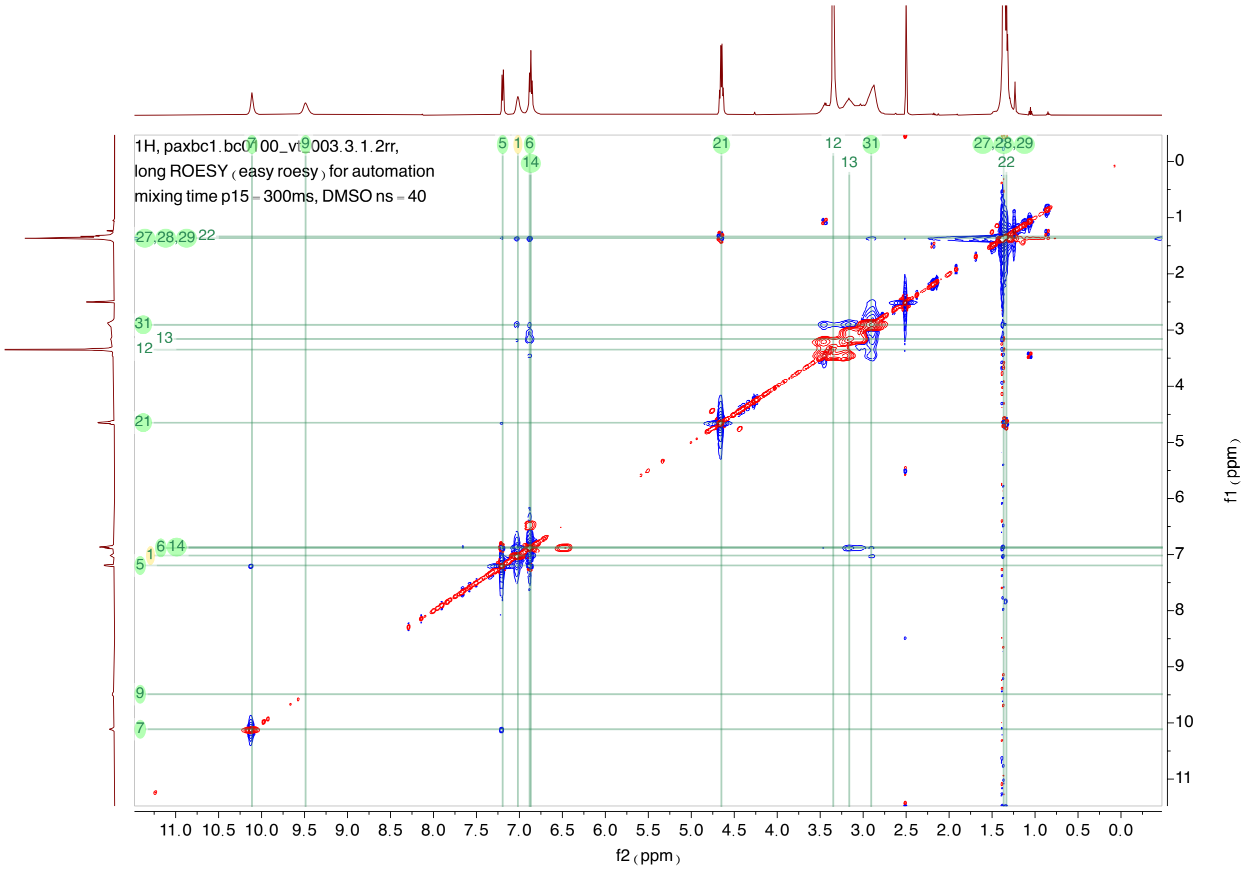

Figure S21. ROESY spectrum of compound **6b**.

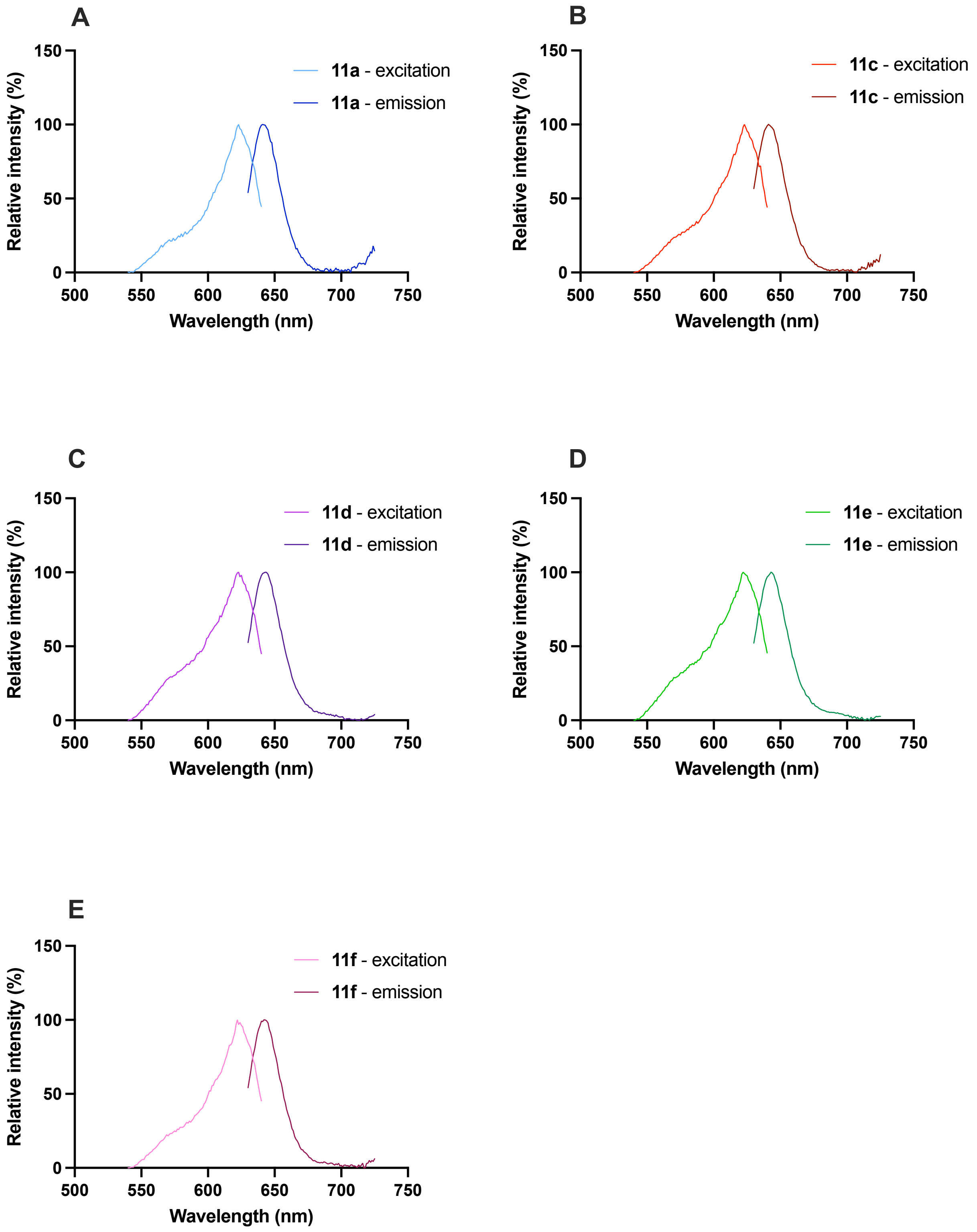

Figure S22: Excitation-emission spectra for 1 μM compounds **11a** (A), **11c** (B), **11d** (C), **11e** (D) and **11f** (E) in MeOH. For excitation analysis, compounds were excited across wavelengths 540-640 nm and emission was detected at 668 nm. For emission scanning, compounds were excited at wavelength 602 nm and emission was detected between 630-725 nm.

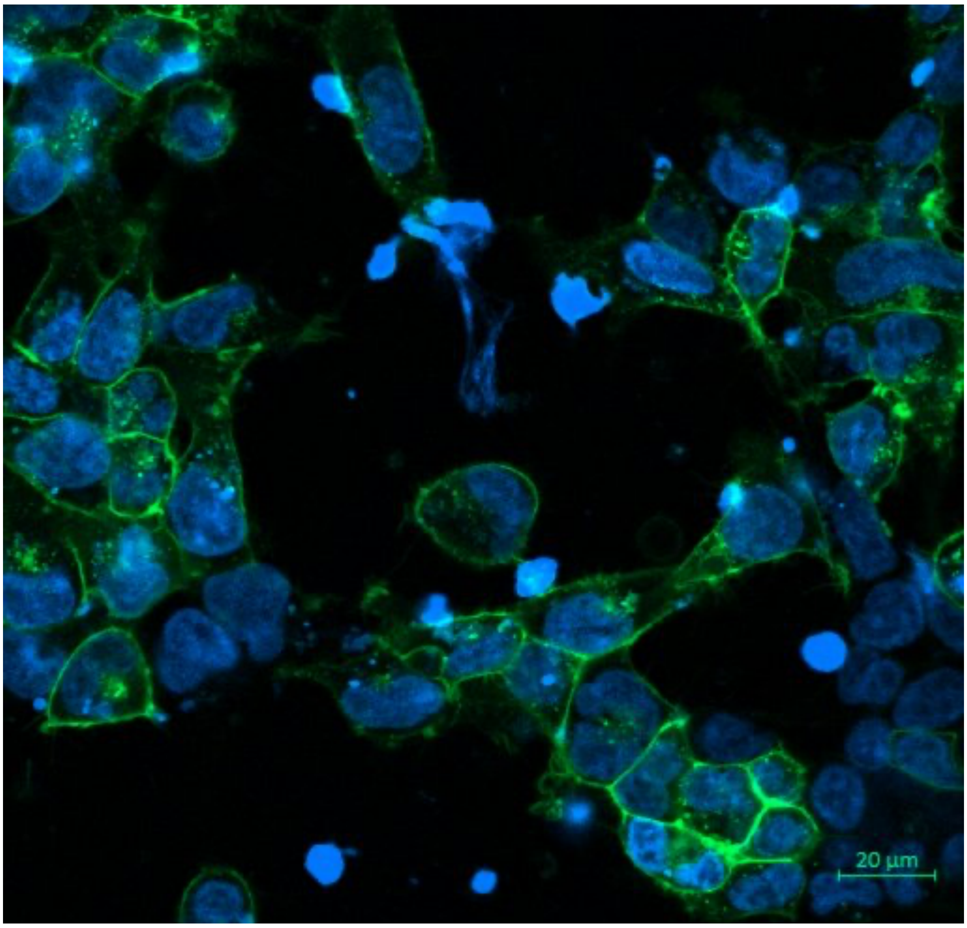

Figure S23: Snap-CXCR2 expression within a HEK293 cell stable line. Cells were identified by bisbenzamide Hoechst 33342 nuclei staining (Blue) and expression of Snap-CXCR2-tsNanoLuc is identified through labelling with SNAPsurface-AF488 (Green).

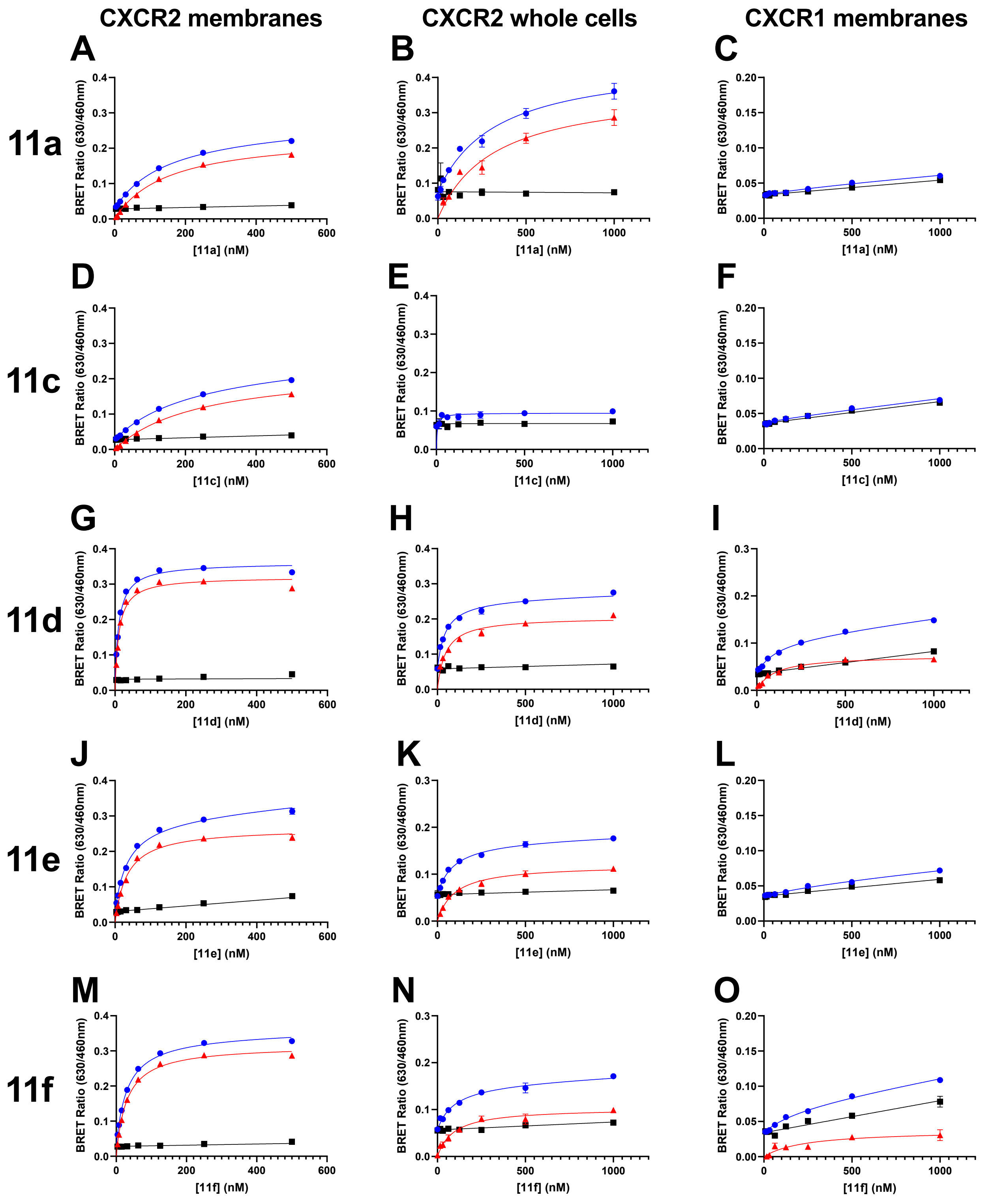

Figure S24. Fluorescent ligands (**11a**, **11c-f**) NanoBRET saturation binding studies in CXCR2 membranes (left column, A, D, G, J, M), CXCR2 whole cells (central column, B, E, H, K, N) and CXCR1 membranes (right column, C, F, I, L, O) in the absence (blue) or presence (black) of 100 nM or 10 μM (*R*)-navarixin (**2**) used to measure non-specific binding (NSB). Specific binding (red) is plotted as the difference between total binding and NSB. Data are representative experiments from n = 5 individual experiments performed in duplicates.
